# Neuropeptide System Regulation of Prefrontal Cortex Circuitry: Implications for Neuropsychiatric Disorders

**DOI:** 10.3389/fncir.2022.796443

**Published:** 2022-06-21

**Authors:** Sanne M. Casello, Rodolfo J. Flores, Hector E. Yarur, Huikun Wang, Monique Awanyai, Miguel A. Arenivar, Rosario B. Jaime-Lara, Hector Bravo-Rivera, Hugo A. Tejeda

**Affiliations:** ^1^Unit on Neuromodulation and Synaptic Integration, National Institute of Mental Health, National Institutes of Health, Bethesda, MD, United States; ^2^National Institute on Alcohol Abuse and Alcoholism, National Institutes of Health, Bethesda, MD, United States

**Keywords:** prefrontal cortex, dynorphin, enkephalin, corticotropin-releasing factor, cholecystokinin, somatostatin, neuropeptide Y, vasoactive intestinal peptide

## Abstract

Neuropeptides, a diverse class of signaling molecules in the nervous system, modulate various biological effects including membrane excitability, synaptic transmission and synaptogenesis, gene expression, and glial cell architecture and function. To date, most of what is known about neuropeptide action is limited to subcortical brain structures and tissue outside of the central nervous system. Thus, there is a knowledge gap in our understanding of neuropeptide function within cortical circuits. In this review, we provide a comprehensive overview of various families of neuropeptides and their cognate receptors that are expressed in the prefrontal cortex (PFC). Specifically, we highlight dynorphin, enkephalin, corticotropin-releasing factor, cholecystokinin, somatostatin, neuropeptide Y, and vasoactive intestinal peptide. Further, we review the implication of neuropeptide signaling in prefrontal cortical circuit function and use as potential therapeutic targets. Together, this review summarizes established knowledge and highlights unknowns of neuropeptide modulation of neural function underlying various biological effects while offering insights for future research. An increased emphasis in this area of study is necessary to elucidate basic principles of the diverse signaling molecules used in cortical circuits beyond fast excitatory and inhibitory transmitters as well as consider components of neuropeptide action in the PFC as a potential therapeutic target for neurological disorders. Therefore, this review not only sheds light on the importance of cortical neuropeptide studies, but also provides a comprehensive overview of neuropeptide action in the PFC to serve as a roadmap for future studies in this field.

## Introduction

Neuropeptides are widely distributed in the central nervous system (CNS) where they regulate various biological effects (Herbert, 1993; van den Pol, 2012; Kash et al., 2015; Nusbaum et al., 2017; Castro and Bruchas, 2019; Brockway and Crowley, 2020; Eiden et al., 2020). Currently, most neuropeptide studies are constrained to subcortical brain structures and tissue outside of the CNS. With the increase in investigations centered on cortical and limbic circuit function underlying higher-order cognition processes, the gap in knowledge of neuropeptide function in cortical circuits has become increasingly apparent. Thus, an increased emphasis on research examining neuropeptide modulation of cortical circuits is needed, particularly as we seek to identify potential therapeutic targets for psychiatric disorders (Salio et al., 2006; Nassel, 2009; Hoyer and Bartfai, 2012; Crowley and Kash, 2015; Smith et al., 2019). In this review, we highlight established knowledge and unknowns of dynorphin, enkephalin, corticotropin-releasing hormone, cholecystokinin, somatostatin, neuropeptide Y (NPY), and vasoactive intestinal peptide (VIP) action in the prefrontal cortex (PFC). This particular set of neuropeptides was chosen to investigate because of their abundance in discrete cells and critical role in motivational and cognitive behaviors that are relevant to a plethora of psychiatric disorders. In turn, we discuss the role of these peptides in regulating executive function, affective behavior, and the role of cortical neuropeptide dysregulation in neuropsychiatric disorders. Changes in neuropeptide expression and action could play a direct role in cortical dysfunction underlying neuropsychiatric disorders as well as enact indirect and widespread effects that have profound implications for systems dysregulation. Leveraging knowledge of neuropeptide action in cortical circuits with insights to cortical circuit dysfunction can help develop more focused and effective treatments that lead to improved therapy (Poyner et al., 2000; Roth, 2019), irrespective if a specific neuropeptide is key to the etiology and/or maintenance of the disorder. Ultimately, we provide a comprehensive overview of neuropeptide action in the PFC by establishing knowns and unknowns, discussing the potential role of neuropeptides in neuropsychiatric disorders and animal models, and providing insights for future research.

### Neuropeptides

Neuropeptides are small proteins, composed of 3–100 amino acid residues, encoded by over 70 genes (Snyder and Innis, 1979; Herbert, 1993; Salio et al., 2006; Burbach, 2011; van den Pol, 2012; Kash et al., 2015; Nusbaum et al., 2017; Castro and Bruchas, 2019; Brockway and Crowley, 2020; Eiden et al., 2020; Fricker et al., 2020). Like amino acid and monoamine neurotransmitters, neuropeptides are signaling molecules used by nerve cells to communicate with other neurons, glial cells, and peripheral cells (Gozes et al., 2001). Relative to other signaling molecules, the neuropeptide class is diverse, and is hypothesized to mediate communication across longer time scales and larger volumes leading to broad, long-lasting modulation of neural processes (Kim et al., 2017). Despite this diversity, all neuropeptides share certain key characteristics that govern their signaling: expression and biosynthesis in neurons and peripheral cells, regulated release, the ability to regulate neural function via actions with receptors and/or ion channels, and processing/degradation upon release to terminate and/or modify signaling (Burbach, 2011). In addition to expression in peripheral cells, gene expression and biosynthesis of neuropeptides is associated with neurons (Baraban and Tallent, 2004). Most excitatory and inhibitory neurons in the cortex show expression of neuropeptides or a neuropeptide binding G-protein coupled receptor (GPCR; Tasic et al., 2018; Smith et al., 2019). A recent single-cell RNA-seq study reporting neuropeptide and neuropeptide-selective GPCR expression patterns in mouse neocortical neurons suggest that neuropeptidergic networks may exist within cortical circuits (Smith et al., 2019). These, and the evolving number of publicly available single-cell RNA-seq and *in situ* hybridization data sets, such as those from the Allen Brain Institute, serve as valuable resources to identify putative peptide-expressing brain regions and/or cell types.

Upon translation, neuropeptide precursor proteins undergo proteolytic processing, an activity dependent process controlled by intracellular calcium, that produces active neuropeptides (Hallberg, 2015; Hook et al., 2018; Fricker et al., 2020; Lee and Fields, 2021). Precursor proteins can yield a single neuropeptide, multiple distinct neuropeptides, and/or multiple copies of a single neuropeptide. A single precursor peptide giving rise to multiple copies of the same or related peptide is energetically advantageous and a means for signal amplification (Salio et al., 2006; Li and Kim, 2008; Hook et al., 2018; Fricker et al., 2020). This translational step results in unique families of related neuropeptides with similar physiological function (Hook et al., 2018) and is the first step toward the regulated secretory pathway, another key characteristic that neuropeptides share.

Neuropeptides are stored and released from dense core vesicles (DCVs) which are larger than the small, clear synaptic vesicles (SVs) and small to intermediate-sized vesicles that store and release amino acid and monoaminergic neurotransmitters, respectively (Martin, 1994; Kim et al., 2006; Russo, 2017). DCVs and SVs are differentially sensitive to stimuli that trigger exocytosis. Specifically, they may recruit different Ca^2+^ sensors, allowing for independent regulation of exocytosis in a temporally- and activity-dependent manner (Zhang et al., 2011; Sudhof, 2012; Kim et al., 2017). Neuropeptide release from DCVs is triggered by small elevations in the Ca^2+^ concentration in the cytoplasm, whereas secretion of amino acids from SVs requires higher elevations, as produced in the vicinity of Ca^2+^ channels near the active zone at synapses (Hokfelt et al., 2000; Tallent, 2008; Nusbaum et al., 2017). These variations in DCV and SV sensitivity to exocytosis triggering stimuli may be important for dictating where neuropeptides are capable of being released. Specifically, SVs aggregate near regions on the presynaptic membrane containing release sites, where DCVs are more randomly distributed throughout the cell (Bean et al., 1994). However, nuanced organization of DCV clustering may exist that has yet to be revealed as studies have shown DCVs containing neuropeptides near dopamine receptors (Svingos et al., 1999), in close proximity to release sites (Song et al., 2020), or away from the active zone (Liguz-Lecznar et al., 2016). This indicates not only that DCVs can be released at multiple sites, but also that various combinations of expression and release patterns exist (Chini et al., 2017). Relative to fast neurotransmitters that bind to their cognate receptors with low affinity, neuropeptides bind with high affinity to GPCRs (Tallent, 2008; Burbach, 2011). It is important to note that following release, regulatory proteases can act on neuropeptides to modify their bioactivity. These modifications can cause inactivation, decrease or increase affinity for GPCR targets, or even confer selectivity for GPCR or ion channels beyond the initial GPCR targets (Mentlein, 2004; Hook et al., 2018). In summary, both the increase in sensitivity of release and variation in location of DCVs leads to neuropeptide signaling properties that shape circuit activity in a spatially- and temporally- distinct manner relative to fast transmitters.

### G-Protein Coupled Receptors

When released from their DCVs, neuropeptides bind to their cognate GPCRs to initiate signaling. These receptors constitute the largest family of transmembrane proteins and mediate cellular responses to hormones, neurotransmitters, and neuropeptides through interaction with their extracellular loop binding pockets (Rosenbaum et al., 2009; Heldin et al., 2016). Some neuropeptides exhibit a high degree of promiscuity across GPCRs and can bind several receptor subtypes (Devi, 2001; Hoyer and Bartfai, 2012; Hook et al., 2018). Importantly, GPCRs are the therapeutic target of 34% of FDA-approved medications, highlighting the importance of neuromodulatory systems (Roth, 2019). Although the GPCRs we discuss in this review bind to endogenous neuropeptides that have been identified and characterized, it is important to note many GPCRs have not yet been linked to endogenous ligands and are designated as orphan GPCRs (Tang et al., 2012). Conversely, many neuropeptides have been described and it is unclear what the repertoire of GPCR activation is for these peptides [i.e., cocaine and amphetamine related transcript peptide (CART)]. Neuropeptides interact with proximal G proteins and other GPCR-associated signaling molecules, except in instances where neuropeptide release sites and receptor location are mismatched or not proximally located (Herkenham, 1987). Nevertheless, neuropeptides can bind distal G-protein and receptor sites via volume transmission (van den Pol, 2012). G proteins are heterotrimeric, specialized proteins comprised of an alpha, beta, and gamma subunit (Weis and Kobilka, 2018; Wootten et al., 2018). Upon agonist-induced activation of the GPCR, GDP is exchanged with GTP on the alpha subunit to initiate G-protein-mediated signaling and the separation of the alpha subunit and beta-gamma dimer (Bruchas and Chavkin, 2010; Burbach, 2011; Weis and Kobilka, 2018; Wootten et al., 2018; Lemos Duarte and Devi, 2020; Zastrow and Sorkin, 2021). Subsequently, each subunit interacts with secondary transducers that amplify signaling cascades or impact the function of regulators of intrinsic excitability or synaptic transmission, such as voltage-gated or ligand-gated ion channels. GPCR function and signaling is not limited to the plasma membrane, however, even after internalization GPCRs can continue to signal from endosomes (Zastrow and Sorkin, 2021). There are four subcategories of GPCRs determined by their alpha-subunit: G_*i/o*_, G_*q/*11_, G_*s*_, G_12/13_, and G_*olf*_ (Rosenbaum et al., 2009). Each subtype of G protein alpha subunit has different modulatory effects and enacts different signaling cascades. A simple but useful oversimplification is that G_*q*_, G_*s*_, and G_12/13_ are stimulatory where G_*i/o*_ is inhibitory. G_*q*_ activates the phospholipase C (PLC) pathway enacting critical second messengers that mediate calcium signaling. G_*s*_ activates the formation of cyclic adenosine monophosphate (cAMP) and subsequently activation of the protein kinase A pathway. G_*i/o*_-coupled GPCRs inhibit cAMP formation and recruit G-protein coupled inwardly rectifying channels (GIRKs) or inhibit voltage-gated Ca^2+^ channels to decrease intrinsic excitability. G_*i/o*_ and G_*s*_ (Cahill et al., 2014; Gangarossa et al., 2019; Jiang et al., 2021) coupled GPCRs also evoke MAPK/ERK dimers through GRK/β-arrestin accessory proteins. MAPK and ERK both regulate targets in the cytosol and translocate to the nucleus where they phosphorylate a variety of transcription factors that regulate gene expression. The resulting signal transduction mechanism through each of the four G-protein subtypes may differ depending on receptor ligand, a phenomenon called functional selectivity or biased agonism (Weis and Kobilka, 2018; Wootten et al., 2018; Faouzi et al., 2020; Wingler and Lefkowitz, 2020). Biased agonists activate an isolated portion of a receptor’s potential signaling pathways and either have no effect on or inhibit the other signaling cascades. For example, a G protein-biased agonist may only activate the G-protein dependent signaling but result in minimal to no activation of β-arrestin-dependent cascades. Conversely, GRK/β-arrestin biased agonists preferentially activate MAP kinase signaling cascades while sparing or minimally impacting G-protein signaling. Thus, modulatory effects of neuropeptides through GPCR signaling are dependent on the pathways in which they enact.

### The Prefrontal Cortex

A detailed description of prefrontocortical function and architecture is beyond the scope of this review. Therefore, we invite the reader to the following review articles for a more in depth look at cortical architecture and circuit function (Carlen, 2017; Fishell and Kepecs, 2020; Ibrahim et al., 2020; Le Merre et al., 2021; Murray and Fellows, 2021; Tejeda et al., 2021). In this review, we focus on aspects of cortical circuits directly relevant to understanding the role of the neuropeptides in the PFC.

Studies using rodents have established the PFC is subdivided into three categories including the dorsomedial PFC (dmPFC), ventromedial PFC (vmPFC), and ventrolateral PFC (vlPFC) (Le Merre et al., 2021). Each subdivision contains distinct cortical subregions that share connectivity and anatomical features (Carlen, 2017). These subregions include the anterior cingulate and dorsal prelimbic (of the dmPFC), infralimbic and ventral prelimbic PFC (of the vmPFC), and orbital frontal cortex (of the vmPFC and vlPFC). Cortical neurons are diverse and can be categorized according to different characteristics, including morphology, patterns of local and long-range connectivity, intrinsic physiology, type of fast neurotransmitter released, and in some cases the neuropeptides they express. The PFC is the brain region with the most connections to other brain regions (Carlen, 2017; Le Merre et al., 2021). Cortical excitatory projection neurons send efferents to a wide array of target brain regions, including limbic structures such as the amygdala, thalamus, multiple nodes of the basal ganglia including the striatum, hypothalamus, monoaminergic centers of the midbrain, and the periaqueductal gray area. Inputs to the PFC arise from various associative and primary sensory cortices, thalamus, hypothalamus, and monoaminergic centers of the midbrain, hindbrain, as well as limbic regions, including the amygdala and ventral hippocampus. Prefrontal cortical circuits also contain a plethora of inhibitory neurons, which serve to limit the activity of principal excitatory neuron and/or disinhibit them (Ferguson and Gao, 2018; Fishell and Kepecs, 2020; Wang et al., 2020). Classes of cortical neurons are differentially localized across cortical layers and extensively interconnected (Harris and Shepherd, 2015; Carlen, 2017; D’Souza and Burkhalter, 2017; Adesnik and Naka, 2018). Cortical circuits integrate these intracortical connections with subcortical connections and local circuit motifs embedded in the microcircuits process them (Harris and Shepherd, 2015). The laminar structure of the PFC is elaborate and neuron location within layers underlies function (D’Souza and Burkhalter, 2017). For instance, excitatory outputs of cortical circuits tend to have layer specificity (Adesnik and Naka, 2018). These circuit motifs regulate cortical processes in various ways including, signal amplification via recurrent excitatory connections, lateral inhibition via poly-synaptic inhibition, generation and/or maintenance of circuit-wide oscillatory activity, and mechanisms for signal convergence and divergence. Despite decades of research and significant advances in knowledge of nuanced synaptic connectivity of cortical networks and microcircuits therein, there are still many unknowns regarding how information is processed beyond fast excitatory and inhibitory connections. Therefore, uncovering more about how the cortex is organized is necessary to understand the extent to which neuropeptides modulate circuit function and behavior.

### Neuropeptides in the Prefrontal Cortex

**FIGURE 1:**
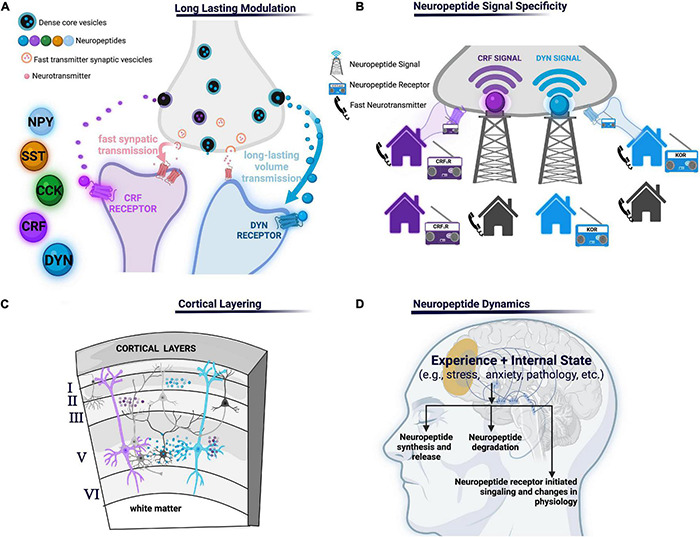
**(A)** Long-lasting modulation: neuropeptides may mediate communication across longer time scales and larger volumes leading to long-lasting modulation of neural processes relative to shorter volumes and shorter duration of other molecules such as fast amino acid neurotransmitters. **(B)** Neuropeptide signal specificity: neuropeptide transmitters can only be detected if the appropriate neuropeptide receptor is present (like a radio signal the neuropeptide signal can only be received if the radio is tuned to the corresponding radio station, unlike a phone which receives point-to-point calls). **(C)** Cortical layering: different cortical layers may have different peptides or receptors and/or concentrations due to various factors including, but not limited to, differential arborization of PFC neuropeptide- and receptor-expressing cells, concentrations of degrading enzymes, or afferent inputs containing presynaptic receptors. **(D)** Various aspects of neuropeptides transmissions are subject to change, including neuropeptide production and release, degradation by peptidases, or signaling depending on the experience or internal state of an organism. NPY, neuropeptide Y; SST, somatostatin, CCK, cholecystokinin; CRF, corticotropin-releasing factor; KOR, κ-opioid receptor, CRF_1_R, corticotropin-releasing factor type 1.

In the present review, we propose that neuropeptides, in the PFC, act as specialized modalities of communication that convey cellular and synaptic specificity via integration of neuropeptide-producing neurons, enzymes that degrade them, and cells bearing cognate receptors to their specific neuropeptide or family of neuropeptides (Figure 1A). For example, consider communicating by cell phone as compared to listening to National Public Radio (NPR): a cell phone transmits a direct signal from one phone to another whereas a radio tower broadcasts a widespread message that is only detected by radios tuned to a specific channel frequency. Radios tuned to the specific channel frequency (NPR) will receive the message, while non-tuned radios will not. In this analogy, the cell phone represents fast-neurotransmitter communication and the radio represents neuropeptide communication (Figure 1B). Virtually every neuron has both ionotropic glutamate and GABAergic receptors localized to post-synaptic densities that receive specific connections from specific neurons arising from local circuits or long-range afferents. Synaptic connections between neurons by fast transmitters are akin to direct calls made via cell phones where every person has the capacity to answer or make direct calls to their neighbors or friends and family. Conversely, neuropeptidergic transmission is like radio communication where messages are broadcast globally (neuropeptide volume transmission), but since not every neuron expresses every neuropeptide receptor (e.g., not all radios are tuned in to the correct frequency) neuropeptide transmission confers specificity in the circuit. This provides cells with specific neuropeptide expression to selectively control circuit elements endowed with complementary cognate receptor.

Other principals by which neuropeptides orchestrate PFC circuit function remain unknown. For example, temporal differences in the timescale of neuropeptide action and signaling contributes to a varied timeframe of circuitry regulation found across different neuropeptides. How this temporal difference specifically impacts the effect of neuropeptide action on the circuit has not been thoroughly investigated. Furthermore, a contributor to the varied timeframe of circuitry regulation seen across neuropeptides is the presence and action of peptidases. Specifically, peptidases limit neuropeptide diffusion and prematurely terminate its action. A small number of studies indicate peptidase regulation of neuropeptide action influences neural circuit activity (Wood and Nusbaum, 2002; Trieu et al., 2022). However, little is still known and investigation of peptidase action on circuit dynamics remains a promising area of study. Moreover, although it has been established that the PFC is reliant on its intricate, layered organization for proper function, much remains unknown regarding the integration of neuropeptides and their cognate receptors into this structure (Figure 1C). Identifying layer differences in neuropeptide, receptor, peptidase localization, and effective neuropeptide concentrations across layers will be essential to increase our understanding of where neuropeptide signals originate and how they finally impact PFC circuits. Further, it is also of importance to establish how the aforementioned nuances of neuropeptide systems integrated in PFC circuits resonate with nuanced layering of neuronal arborization location of different neuropeptide and peptide receptor-containing neurons and their physiological properties. For instance, if a neuropeptide is expressed in one layer of the PFC and the receptor in another it gives the cortex layer specific intercommunication capacities. Additionally, if the branching patterns of different neuropeptide expressing neurons differ, the layers in which their arborizations reside may also differ. In turn, this leads to variability in innervation by layer specific inputs. Finally, neuropeptide action is highly intertwined with internal states and experience influenced by context, exteroceptive and interoceptive cues, motivation, arousal, and affect (Kennedy et al., 2014; Zelikowsky et al., 2018). For example, the internal state of an organism significantly regulates neuropeptide transmission through various facets. Specifically, internal state may impact neuropeptide release, peptidase activity, cognate receptor expression and function, or downstream signaling. Additionally, neuropeptides themselves may influence the maintenance or transitions between internal state or be changed as a consequence of internal state (Figure 1D). However, further investigation of the dynamic nature of internal state and neuropeptide action is required. For example, the field has yet to fully understand the dependence of PFC neuropeptide expression and function on internal state. As a last example, if a neuropeptide receptor impacts an ion channel that is preferentially utilized in specific sub-populations, then that neuropeptide system will have a cell-selective effects even if the receptor is widely expressed. Further delineating these principals is a promising avenue to further investigate how different neuropeptides differentially fit into cortical circuits.

Neuropeptide modulation of cortical circuits modifies PFC processing of sensation, perception, decision-making, cognition, and/or affective behaviors. Dysregulation of the PFC is associated with various psychiatric disorders, including depression (Hare and Duman, 2020), anxiety (Park and Moghaddam, 2017), post-traumatic stress disorder (Koenigs and Grafman, 2009), and substance use disorders (Goldstein and Volkow, 2011). Therefore, the potent regulatory effects of neuropeptides on higher-order cognition processes in the PFC could contribute to symptomology of various psychiatric disorders. To date, there is limited information on how neuropeptides govern cortical circuits and whether there is region-specific regulation of neuropeptide release (Brockway and Crowley, 2020). In turn, throughout this review, to supplement for vast gaps in knowledge of specific neuropeptide function within the PFC, we mention research in other cortical regions or culture systems to make predictions of how neuropeptides may function in the PFC. It is imperative the field further investigations of neuropeptide action within the PFC to uncover these unknowns so ultimately potential therapeutic targets to various psychiatric disorders are revealed.

## Neuropeptide Families

### Dynorphin

Dynorphins (Dyns) are endogenous neuropeptides that play an essential role in regulating nociceptive, cognitive, and affective information. Dyns are synthesized from the precursor prodynorphin (PDyn) (Chavkin et al., 1983), which is cleaved by the enzyme proprotein convertase 2 (PC2) (Day et al., 1998). Cleavage produces three primary forms of Dyn: Dyn A, Dyn B, and big Dyn, which consists of Dyn A and B. Each form shows a high affinity for the kappa opioid receptor (KOR) (Chavkin et al., 1982; Schwarzer, 2009). The most potent activator of KORs, Dyn A 1–17, is a 17 amino-acid peptide (James et al., 1984). Note that there are less potent forms like Dyn A_(1–13)_ and Dyn A_(1–8)_ (Feuerstein and Faden, 1984). The first five amino acids of Dyn encode the peptide Leu-enkephalin which is essential for binding to KORs (Chavkin and Goldstein, 1981). Dyn B and big Dyn (Fischli et al., 1982) also activate KORs but possess lower KOR binding affinities than Dyn A. In addition to KOR, Dyn A_1–8_, a truncated form of Dyn A (Minamino et al., 1980), shows moderate affinity to both μ and δ opioid receptors (Toll et al., 1998). Dyn has also been shown to have non-opioid actions at NMDA receptors (Chen et al., 1995; Caudle and Dubner, 1998; Tang et al., 2000). Triggered by membrane depolarization, Dyn is released from DCVs localized to presynaptic or somatodendritic terminals (Molineaux and Cox, 1982; Chavkin et al., 1983; Whitnall et al., 1983; Svingos et al., 1999). Once released, Dyns mainly target KORs (Wagner et al., 1991), which are inhibitory GPCRs that activate G_*i/o*_ signaling pathways (Taussig et al., 1993). Dyn is hypothesized to mediate inter-cellular communication through volume transmission where the peptide diffuses to binding sites up to 50–100 μm from their release site as measured in the hippocampus (Drake et al., 1994; Castillo et al., 1996; Chavkin, 2000). Although not observed in the PFC, this translates as it suggests that the location of KORs and localization of peptidases that degrade Dyns are essential to understanding the spatiotemporal profile of Dyn/KOR signaling. Together, Dyn signaling through KORs and subsequent downstream effects contribute to sensory and affective information regulation.

Roughly 8% of cortical neurons express PDyn, and 3% express KORs (Smith et al., 2019). Specifically, PDyn is heavily expressed in somatostatin interneurons (Sohn et al., 2014; Smith et al., 2019). PDyn mRNA and Dyn immunoreactivity has been observed across cortical regions in several species (Khachaturian et al., 1985; Peckys and Hurd, 2001; Yakovleva et al., 2006). Still, the relative abundance of this system is over-represented in primates relative to rodents (Hurd, 1996; Merchenthaler et al., 1997; Lin et al., 2006). Thus, the role of cortical PDyn may be more relevant in humans than in rodents and suggests the Dyn/KOR system may play a unique role in humans. In turn, warranting future research. In primary sensory cortices, most PDyn-mRNA expressing neurons are GABAergic somatostatin-containing interneurons (Sohn et al., 2014; Loh et al., 2017b; Smith et al., 2019), whereas, in the PFC, most PDyn-expressing neurons are glutamatergic (Sohn et al., 2014). A recent study using PDyn-iCre mice crossed with tdTomato mice showed strong labeling restricted to superficial layers in the insular cortex (Pina et al., 2020). Rats show temporal shifts in the patterns of cortical PDyn expression at different developmental stages (Alvarez-Bolado et al., 1990) and this dynamic expression pattern is consistent with reported maturational changes in rats’ mPFC Dyn/KOR signaling patterns (Sirohi and Walker, 2015). PDyn expression may also change as a function of activity and experience. A recent study reported a transient experience-dependent increase in the percentage of somatostatin interneurons expressing PDyn (Loh et al., 2017a). This is supported by data that show regulation of human cortex PDyn expression by epigenetic changes linked to psychiatric disorders (Taqi et al., 2011; Yuferov et al., 2011; Butelman et al., 2012; Tejeda et al., 2012; Bazov et al., 2018a). These findings suggest Dyn expression can be used as a marker that represents a dynamic functional state of somatostatin (SST) interneurons that are sensitive to developmental and environmental factors underlying psychiatric disorders. However, further research is needed to validate this model.

The abundance of KOR mRNA expression in the cortex suggests that cortical sources of Dyns should affect local circuits (Meng et al., 1993; Svingos and Colago, 2002; Bazov et al., 2018b). KOR and PDyn mRNA expression patterns suggest that different cortical regions show distinct patterns of layer specificity (DePaoli et al., 1994; Peckys and Hurd, 2001; Smith et al., 2019). For example, in humans PDyn mRNA shows a different pattern of expression in the cingulate and dorsolateral PFC. Specifically, PDyn mRNA is widely spread across layers of the cingulate whereas it is tightly confined to limited layers in the dorsolateral PFC. In contrast, KOR is expressed similarly in both regions. Considering abundant expression of KOR and PDyn mRNA in the cortex, it is currently unclear how Dyn producing cells influence incoming KOR-expressing inputs or local-circuit neurons and unknown whether Dyn is released from axon terminals to act homosynaptically or heterosynaptically. It is also not known whether Dyn can be released from PFC cells from somatodendritic compartments to influence incoming inputs to Dyn neurons in a retrograde manner. Future research is necessary to address these unknowns and dissect Dyn/KOR regulation of PFC inputs. Moreover, KORs are differentially expressed in limbic afferent inputs that project preferentially to the PFC relative to primary sensory cortices (Tejeda et al., 2021). KOR mRNA shows increased expression in layer VI pyramidal neurons projecting within the telencephalon (Tasic et al., 2018; Smith et al., 2019). Ultrastructural studies show that prefrontal KOR immunoreactivity is primarily observed in presynaptic terminals with varicosities and presynaptic terminals of symmetric and asymmetric synapses, indicative of excitatory and inhibitory synapses, respectively (Svingos et al., 1999). Consistent with KOR localization on varicosities, KORs are expressed presynaptically in mesocortical dopaminergic terminals in the PFC and inhibit the release of dopamine (Tejeda et al., 2013). However, the expression of KOR in mesocortical neurons is not limited to presynaptic terminals, as KOR activation directly inhibits the activity of PFC-projecting ventral tegmental neurons (Margolis et al., 2006). KORs also regulate excitatory basolateral amygdala, but not ventral hippocampus, inputs to PFC (Tejeda et al., 2015), suggesting that the Dyn/KOR system may filter information coming into the PFC in a pathway-specific manner. KORs also potently inhibit the glutamate-driven enhancement of extracellular GABA levels, suggesting that Dyn may also regulate excitation/inhibition balance (Tejeda et al., 2013). Pathway-specific Dyn/KOR regulation of excitatory synapses and excitation/inhibition balance in different cell types has also been observed in the nucleus accumbens (Tejeda et al., 2017), which would imply that selective filtering of information flow may be a general principle of the Dyn/KOR system. Inhibitory actions of dynorphin on insular cortex GABA neurons have also been reported (Pina et al., 2020). Collectively, these studies suggest that Dyn/KOR signaling may regulate PFC information processing via multiple mechanisms.

Dysregulation of the Dyn/KOR system has been shown to play crucial roles in drug-seeking, appetitive, and mood disorders. KOR activation promotes aversive behavior in animal models (Mucha and Herz, 1985) and psychotomimetic, anxiogenic, aversive experiences in humans (Pfeiffer et al., 1986). Direct infusion of the KOR antagonist norBNI in either prelimbic or infralimbic cortices diminishes restraint stress-induced increases in arterial pressure and heart rate without affecting stress-induced changes in tail and body temperature (Fassini et al., 2014, 2015). This suggests that KOR signaling in the PFC increases autonomic arousal and may underlie maladaptive stress-related responses, a hallmark of anxiety disorders. Consistent with this notion, mPFC infusion of a KOR antagonist had an anxiolytic effect in an open field (Wall and Messier, 2002; Tejeda et al., 2015). Moreover, systemic or direct injection of KOR agonist in mPFC produces conditioned place aversion (CPA) (Bals-Kubik et al., 1993). Furthermore, CPA induced by systemic KOR agonist is blocked via mPFC infusion of KOR antagonist (Tejeda et al., 2013), indicating that KOR activity in the mPFC is required for KOR-mediated aversion. Other studies have identified a role for the Dyn/KOR system in the infralimbic cortex in reducing anxiety-like behavior. Intra-mPFC administration of the KOR agonist, U69,593, or the KOR antagonist, nor-BNI, reduces and increases anxiety-like behavior, respectively (Wall et al., 2001; Wall and Messier, 2002). Further insight regarding the mechanisms of the Dyn/KOR system’s regulation of autonomic, anxiety-like behavior, and aversive responses may benefit future treatments for patients with anxiety and mood disorders.

The Dyn/KOR system may also regulate cognition via regulation of mPFC circuits. A recent study found that KOR activation in the PFC decreased accuracy and responsiveness in a delayed non-match to sample working memory task (Abraham et al., 2021). These effects were recapitulated by optogenetic activation of PDyn expressing PFC neurons and blocked by local infusion of the KOR antagonist norBNI. Conversely, the Dyn/KOR system in the infralimbic cortex may promote working memory as KOR activation and inhibition enhances and decreases putative measures of working memory on the spontaneous alternation memory task (Wall and Messier, 2002; Wall and Messier 2000a,b). Collectively, these studies point to a potential role of KOR-PFC signaling in PFC-dependent working memory and this capacity may differ depending on PFC sub-regions.

What is currently unknown is how the many actions of Dyn/KOR signaling impacts the function of PFC circuits to influence behavior. Dopamine transmission and limbic inputs in the PFC are critical for cognitive processing, working memory, and decision-making (Goldman-Rakic et al., 2000; Floresco, 2013; Arnsten, 2015). The Dyn/KOR system is widespread across cortical circuits. However, unique innervation of the PFC by KOR-sensitive afferent inputs, such as the ventral tegmental area and basolateral amygdala, that convey motivationally charged information to promote executive control of behavior inherently confers the PFC Dyn/KOR system the capacity to regulate affective behavior and cognitive control. The inhibitory effect of KOR activity in dopamine and basolateral inputs to the PFC, imparts a specialized function for this system in differentially shaping PFC circuits versus primary and lower-order associative cortical circuits. Moreover, Dyn expression in PFC circuits is not restricted to GABAergic neurons, as is observed in primary sensory cortices (Sohn et al., 2014). This suggest that principal neurons may utilize this peptide to influence incoming afferents and dopaminergic inputs, providing a modulatory signal capable of filtering incoming information and local processing. Differential integration of the Dyn/KOR system into PFC along with unique features of the PFC may be essential contributing factors that position the Dyn/KOR system as a potent regulator of affective, defensive, arousal and executive function.

One function of the PFC Dyn/KOR system may be to regulate dendritic integration in cortical cells by virtue of the diverse actions this system has on PFC circuits. Dopamine transmission in the PFC may drive wide-spread cellular effects that culminate in increased encoding of synaptic information within dendritic compartments within specific ensembles of neurons. By directly inhibiting glutamate release via a presynaptic site of action, KOR may decrease the probability of NMDA receptor activation and subsequent recruitment of active conductances, such as voltage-gated calcium channels, to reduce responsivity of neurons to incoming inputs (Palmer et al., 2014; Augusto and Gambino, 2019). KORs localized within dendritic processes may also influence the way KOR-positive cortical cells process information. GABA_*B*_ receptors, G_*i/o*_-coupled GPCRs similar to KOR, inhibit Ca^2+^ signaling through NMDA receptors (Chalifoux and Carter, 2010), which would be predicted to inhibit Ca^2+^-regulated processes in somatodendritic compartments essential for shaping input-output transformations. Occasional co-localization of KOR and NMDA receptors in dendrites (Svingos and Colago, 2002) provides an anatomical basis for interaction between KOR and NMDA receptors similar to those with GABA_*B*_ receptors. KOR activation also influences the mTOR pathway, which modifies neurite outgrowth, spinogenesis, and synaptic plasticity in cortical neurons (Liu et al., 2019), providing another mechanism by which Dyn may regulate dendritic processing in PFC KOR-containing neurons. Lastly, Dyns can positively or negatively allosterically modulate NMDA receptor activity in a KOR-independent manner (Caudle and Dubner, 1998), influencing excitatory transmission or synaptic integration. Collectively, the concerted efforts of Dyn/KOR signaling is expected to have widespread effects on PFC cortical circuit dynamics and may be important for the selection/deselection of ensembles that encode PFC-dependent behavior.

### Enkephalin

Enkephalins (Enks) are endogenous neuropeptides and members of the opioid peptide family that play a significant role in neurotransmission and pain modulation. Enks signal through G_*i/o*_ opioid receptors, preferentially binding to DOR and MOR (Takahashi, 2016). Enk, DOR, and MOR expression is widely distributed throughout the central, peripheral, and autonomic nervous systems, multiple organ systems, as well as endocrine tissues and their target organs (Hughes et al., 1975; Tang et al., 1982; Holaday, 1983; Coffield and Miletic, 1987; Duque-Diaz et al., 2017; Smith et al., 2019). There are two structurally different Enk peptides, Met-Enk and Leu-Enk which arise via proteolytic processing from the precursor proteins proenkephalin A and proenkephalin B, referred to as PDyn. Proenkephalin is enriched throughout the brain and gives rise to Met- and Leu-Enk neurons in various regions including the cerebral cortex, basal ganglia, limbic telencephalic nuclei, hypothalamus, and thalamus (Geracioti et al., 2009). Proenkephalin A yields four Met-Enks, one Leu-Enk, one Met-Enk-Arg^6^-Phe^7^, and one Met-Enk-Arg^6^-Gly^7^-Leu^8^ (Dhawan et al., 1996). In contrast, PDyn yields one Leu-Enk. Leu- and Met-Enks have the highest binding affinity to DOR followed by the MOR (Schafer et al., 1991; Devi, 2001).

Enkephalin and cognate opioid receptor DOR and MOR expression in the cerebral cortex has been thoroughly characterized (Giraud et al., 1983; Taki et al., 2000; Smith et al., 2019). Single-cell RNA sequencing reveals roughly 40% of anterior lateral motor cortex (ALM) and primary visual cortex (VISp) cells contain the pro-enkephalin gene (PENK), 40% contain the gene encoding MOR (Oprm1), and 13% contain the gene encoding DOR (Oprd1). According to single cell sequencing, PENK is primarily expressed in VIP and parvalbumin containing GABAergic neurons, although a small subset of glutamatergic IT neurons also express the gene. Similarly, although Oprm1 is mainly expressed in VIP and somatostatin GABAergic neurons, it is also expressed in a small subset of parvalbumin and layer VI glutamatergic neurons. In contrast, Oprd1 is almost exclusively expressed in somatostatin and PV cells (Smith et al., 2019). Furthermore, immunolabeling methods show neurons expressing MOR are frequently colocalized with proenkephalin, and mainly express on small, non-pyramidal neurons expressing GABA in layers II–IV (Taki et al., 2000). Immunocytochemical detection of opioid peptides in the PFC reveal widespread expression of Enk in layers II and III and V and VI of neocortex in addition to layers II and III of the olfactory cortex (McGinty et al., 1984; McGinty, 1985). Additionally, radioimmunoassays reveal the ratio of Leu-Enk to Met-Enk-Arg^6^-Gly^7^-Leu^8^ is roughly one, which corresponds with the ratio of respective precursor proteins (Giraud et al., 1983). This contrasts with other peptides where the ratio of respective precursor proteins does not correspond with the active peptide, in turn, implying the majority of PENK is converted to active Enk peptide. In summary, Enk and its cognate receptors are expressed across layers of the cortex in both glutamatergic and GABAergic neurons.

Enkephalin impinges on cortical circuitry by regulating synaptic transmission and other cortical neuromodulators. Electrophysiological findings reveal opposing roles of MOR and DOR in synaptic transmission within thalamo-cortico-striatal circuits. Specifically, MOR agonists suppress excitatory thalamic inputs to the ACC, while DOR opioid agonists disinhibit ACC pyramidal neurons by suppressing feed-forward inhibition (Birdsong et al., 2019). Therefore, DOR activation causes hyper-excitable ACC circuits and MOR activation causes inhibition of glutamate release. This suggests Enk action on either MOR or DOR can differentially impact thalamo-cortical-striatal circuitry. Moreover, MOR modulation of cortical circuity is subregion specific. MOR agonists acting on MORs expressed on parvalbumin-interneurons inhibit GABAergic synaptic transmission in the medial orbitofrontal cortex but not lateral orbitofrontal cortex (Lau et al., 2020). MOR antagonism does not reverse this suppression of inhibition which indicates MOR enaction of long-term depression. Ultimately, these regional differences suggest location-dependent differences in MOR coupling to downstream cAMP/PKA signaling cascades or differential expression of functional MORs. Cortical MOR also regulates dopamine action. For example, MOR activation within the PFC elevates local dopamine overflow (Tejeda et al., 2013), which provides a novel mechanism by which MORs may regulate dopamine output beyond disinhibition of dopamine neuron activity within midbrain circuits (Johnson and North, 1992; Matsui and Williams, 2011). Additionally, Enk can modulate cortical circuitry by altering the action of other neuropeptides in the system. D-Ala2-D-Leu5-Enk inhibits K^+^-stimulated release of cholecystokinin in the hypothalamus but not the cortex (Micevych et al., 1985). Contrastingly, cortical D-Ala2-D-Leu5-Enk inhibits K^+^-stimulated release of VIP in a naloxone dependent manner (Micevychi et al., 1984). Together, these results indicate region-specific, elaborate modulation of cortical circuitry by Enk through MOR and DOR.

Activation of DOR and MOR and their signaling cascades by Enk has been shown to modulate information processing in cortical circuits and associated behaviors. Specifically, studies on Enk modulation of neural circuits as contributors to psychiatric disorders (e.g., addiction, anxiety, depression, and PTSD) and pain regulation are currently active areas of research. Adverse life experiences increase lifetime risk to stress-related psychopathologies, and exposure to stress downregulates endogenous Enk expression in the PFC in rats (Li et al., 2018). However, post-mortem cortical tissue from individuals suffering from substance use disorder showed upregulated PDyn and KOR mRNA, but no significant changes in expression of proenkephalin, MOR, and DOR opioid receptor mRNA was evident. This suggests KOR but not MOR signaling may underlie in part the neurocognitive dysfunctions relevant for addiction and disrupted inhibitory control (Bazov et al., 2013; Nosova et al., 2021).

Prefrontal cortex Enk and MOR signaling has been implicated in modulating reward-seeking and compulsive behaviors. MOR stimulation within the vmPFC induces feeding and hyperactivity (Mena et al., 2011, 2013; Giacomini et al., 2021). Additionally, endogenous cortical Enk transmission is both necessary and sufficient for the expression of impulsive action in a high-arousal appetitive states (Selleck et al., 2015) as well as anticipation and excessive consumption of alcohol (Morganstern et al., 2012). Opioid action in the PFC has been implicated in inhibitory-control deficits associated with addiction and binge-type eating disorders. For example, prenatal ethanol exposure led to increase in Met-Enk in the PFC which is suggested to underlie the facilitation of postnatal ethanol intake (Abate et al., 2017). Furthermore, cocaine self-administration significantly increases MOR and DOR mRNA but not proenkephalin mRNA in the PFC indicating changes in these targets may underlie cocaine-induced reward and habitual drug-seeking behavior (Sun et al., 2020). Cortical Enk may also regulate compulsive behaviors as models of autism spectrum disorder demonstrate a decrease in cortical endogenous Enk with an increase in repetitive behaviors (Augustine et al., 2020).Together, these studies suggest the role of Enk signaling through MOR and DOR in cortical circuit processing and associated behaviors underlying neurological disorders.

Changes in Enk cognate receptor availability also impacts cortical circuits which regulate reward and motivational processes that underlie psychiatric disorders. Specifically, positron emission tomography (PET) studies show a decrease in MOR opioid peptide binding potential in anterior cingulate for patients with PTSD (Liberzon et al., 2007). Additionally, cortical PET studies have revealed the binding potential of MOR and DOR ligands in patients with chronic neuropathic pain. Individuals experiencing central post-stroke pain had a decrease in [^11^C]-diprenorphine, a non-selective opioid receptor antagonist, in the insular and lateral prefrontal cortices and anterior cingulate (Willoch et al., 2004; Maarrawi et al., 2007a). Patients with peripheral neuropathic pain had symmetrical bilateral decreases in [^11^C]-diprenorphine where patients with central post-stroke neuropathic pain primarily had contralateral asymmetrical decreases. This suggests that motor cortex stimulation (MCS) for control of neuropathic pain also decreases [^11^C]-diprenorphine binding in the anterior cingulate indicating enhanced secretion of endogenous opioids during MCS (Maarrawi et al., 2007b). However, it is unclear from these results whether decrease in opioid receptor availability is due to a loss of opioid-receptor containing neurons, available receptors, and/or increased endogenous opioid peptide release. Ultimately, given that endogenous enkephalin signaling via MOR and DOR regulates excitation-inhibition balance in a circuit specific manner, alterations in opioid receptor availability following stroke may contribute to mal-adaptive behaviors and is suggested to be one of the causes of poststroke pain. In turn, upregulation and action of MOR and DOR in the cortex indicates modulation of cortical circuits underlying pain disorders.

Given the role of Enk underlying psychiatric disorders and suggested modulation of information processing in cortical and subcortical circuits, the use of Enk as a therapeutic has been highly considered. It is important to note that therapeutics pertaining to MOR and DOR are vast, and for the purpose of this review only therapeutic treatments that aim to manipulate Enk as a peptidergic transmitter will be discussed. For a more detailed look at opioid receptor specific therapeutics please consult the following articles (Broom et al., 2002; Spetea et al., 2013; Browne et al., 2020; Grim et al., 2020; Senese et al., 2020). To capitalize on the analgesic effects of endogenous Enks, research has been done to chemically modify Enks so they are more difficult to degrade and analgesic properties of endogenous Enks can be amplified while retaining their ligand specificity for MOR and DOR (Pert et al., 1976; Kropotova et al., 2020). A result of these studies is the development of dual enkephalinase inhibitors (DENKIs) that enhance the analgesic effects of endogenous Enks only at sites of release, avoiding negative side effects including tolerance, respiratory depression, and constipation that derive from widespread MOR and DOR activation (Poras et al., 2014). Despite these advantages, peptidase inhibitors act on many targets and unexpected, negative impacts of their inhibition is a caveat that must be considered. Further, the coupling of Enk to the glycosylation of biopharmaceuticals improved binding to its carbohydrate receptor, pioneering a method that increases the accuracy of therapeutic peptides (Christie et al., 2014). Together, Enk is a promising therapeutic to consider for pain and psychiatric disorders.

Despite the number of studies on the neuromodulatory effects of Enk underlying analgesia and neuropsychiatric-relevant behaviors, there are still many unknowns of Enk function in cortical circuits. For example, although Enks precursor peptides proenkephalin A and Pdyn have been well established, whether there are differential effects of Enk sources arising from proenkephalin A or Pdyn has yet to investigated. Additionally, although Birdsong et al. (2019) established that MOR and DOR regulate the thalamo-cortico-striatal circuit in opposing ways and Lau et al. (2020) determined subregional differences in MOR-coupling downstream cascades, whether MOR and DOR differential regulation of the cortex works independently alongside one another or together is unknown. Furthermore, VIP neurons disinhibit the cortex by inhibiting other cortical interneurons (Millman et al., 2020). Given the colocalization of Enk and VIP expression (Smith et al., 2019; Leroy et al., 2021) and that GABAergic interneuron output at some capacity are inhibited by Enk, Enk release by cortical VIP neurons may cooperate with VIP GABAergic transmission to disinhibit cortical circuits in a spatially- and temporally organized manner. As imbalance in excitatory and inhibitory control of PFC circuits is implicated in various neuropsychiatric disorders, it will be of importance to understand how inhibitory effects of MORs on excitatory inputs versus disinhibition by MOR/DOR may play a role in such excitation/inhibition imbalance. Ultimately, understanding the role of Enk in PFC circuits is necessary to uncover the underpinnings of various psychiatric disorders and further develop therapeutic targets and treatments.

### Corticotropin-Releasing Hormone

Corticotrophin releasing factor (CRF) plays a significant role in the integration of endocrine and behavioral responses to stress by acting on the hypothalamic-pituitary-adrenal (HPA) axis (Vale et al., 1981). However, CRF is also expressed in brain regions not associated with the HPA axis such as the cortex. Originating from a 196 amino acid prepropeptide, CRF is synthesized by prohormone convertases at dibasic amino acids (lysine or arginine) to produce a 41-amino acid mature peptide (Hook et al., 2008). CRF is a member of the CRF system comprised of CRF and three additional neuropeptides: Urocortin I (UCNI), Urocortin II (UCNII), and Urocortin III (UCNIII). As there is little known regarding Urocortins in cortical circuits, this review will only cover CRF within the CRF system. CRF has two cognate GPCR receptors, CRF type-1 (CRF1) and CRF type-2 (CRF2) (Dedic et al., 2018), which are fairly similar and share 70% amino acid identity. Despite this similarity, CRF receptors differ in their N-terminal extracellular domain (40% identity) (Dautzenberg and Hauger, 2002). CRF1 has only one functional splice variant expressed in the brain where CRF2 receptor has three functional splice variants in humans (α, β, and γ) and two in rodents (α and β) (Dedic et al., 2018). CRF2α receptor serves as the major rodent splice variant (Chalmers et al., 1996).

Corticotrophin releasing factor peptides act through CRF receptors with varying affinity (Dedic et al., 2018). Specifically, CRF has a higher affinity for the CRF1 than CRF2 receptor. Although CRF receptors couple primarily to Gs proteins (Millan et al., 1987), they can bind and activate other G proteins as well (Grammatopoulos and Chrousos, 2002; Hillhouse and Grammatopoulos, 2006). This suggests CRF receptors may couple to various signaling pathways. CRF1 activation by CRF or UCNI and CRF2 receptor activation by UCNI, UCNII, or UCNIII can activate ERK1/2 in CHO cells (Brar et al., 2004). Moreover, CRF selectively activates ERK1/2 in different regions of the brain via CRF1 *in vivo* (Refojo et al., 2005). In PFC synaptosomes, UCNIII induces activation of ERK1/2 (Yarur et al., 2020a). Together, these studies indicate CRF receptor signaling pathways are determined by specific G-protein coupling and availability of cellular signaling components in CRF-containing cells.

Corticotrophin releasing factor type-1 and CRF2 are expressed in the olfactory bulb, bed nucleus of the stria terminalis, lateral septum, paraventricular nucleus of the thalamus, dorsal raphe nucleus, and mPFC where expression of CRF1 is greater than CRF2 (Van Pett et al., 2000). In the mPFC, CRF is expressed in neurons across all layers (Yan et al., 1998). CRF is expressed in inhibitory interneurons, primarily in parvalbumin cells and rarely in calbindin or calretinin-expressing cells (Yan et al., 1998). UCNs expression in the cortex is present in sparse fibers in deeper layers (Bittencourt et al., 1999). CRF1 receptor is widely expressed in pyramidal cells of the mPFC (Uribe-Marino et al., 2016). There is also evidence of CVRF1 expression in inhibitory interneurons as the receptor is often co-expressed with other peptides found in various interneuron populations like somatostatin, VIP, and cholecystokinin (Gallopin et al., 2006). To date, there is no conclusive evidence showing the expression of CRF2 receptor within mPFC circuits (Van Pett et al., 2000). However, CRF2 receptor expression on basolateral amygdala inputs to the mPFC have been described (Yarur et al., 2020b). Collectively, these studies suggest that the CRF system is integrated into PFC circuitry and may regulate circuit dynamics.

Studies show CRF modulates PFC synaptic transmission. Iontophoretic application of CRF enhances the activity of neurons in the cortex of Sprague–Dawley rats (Eberly et al., 1983). In addition, CRF bath application increases sEPSC frequency in layer V pyramidal cells from rat PFC slices, an effect that can blocked a by a CRF1 receptor antagonist (Liu et al., 2015). These results suggest that CRF enhances excitatory drive onto PFC principal neurons. Interestingly, lesions of the basolateral amygdala reduced CRF enhancement of sEPSC frequency of ipsilateral layer V pyramidal cells in PFC (Liu et al., 2015), suggesting that basolateral amygdala synapses are a site of action for CRF. Similar results were found in layers II/III and V of adult male C57BL/6J mice (Hwa et al., 2019), where the increases in sEPSC frequency induced by fox odor were ablated by the administration of a systemic CRF1 receptor antagonist. Pretreatment with CP154526, a CRF1 antagonist, suppresses defensive burying and reduces enhanced synaptic transmission elicited by predator exposure. The increase in sEPSC by fox odor exposure occludes the increase of sEPSC induced by CRF bath application, implying that predator odor-related behaviors engages the CRF system in the PFC. Collectively, these studies are consistent with a facilitatory role of CRF on excitatory transmission in the PFC. CRF may also interact with the serotonin system within PFC circuitry. PFC 5-HT signaling has been implicated in anxiety and stress behaviors. 5-HT increases the amplitude and frequency of sIPSCs in the rat PFC (Tan et al., 2004). Co-application of CRF and 5-HT prolong the effects of 5-HT on sIPSC, an effect blocked by astressin, a CRF1 antagonist. Collectively, these studies suggest that CRF regulates synaptic transmission in the mPFC and may be recruited by stressors.

Prior studies reveal dynamic changes in cortical CRF systems in response to stress. Acute restraint stress increases CRF and CRF1 mRNA in the PFC of Sprague–Dawley rats (Meng et al., 2011). On the other hand, chronic social defeat stress decreased CRF mRNA and increased CRF1 mRNA in PFC of Wistar rats (Boutros et al., 2016). In adult males, chronic social defeat stress modified mRNA for CRF receptors only in susceptible animals, but not resilient mice (Guo et al., 2020). In comparison to control and resilient mice susceptible mice, susceptible mice show increased CRF1 mRNA and decreased CRF2 receptor mRNA expression. These results suggest condition and duration of the stressor can differentially regulate CRF to contribute to stress-coping deficits with chronic stressors.

Corticotrophin releasing factor modulation of PFC function and behavior is a rising area of study. Infusion of CRF in the PFC impairs working memory while an infusion of NBI 35965, a CRF1 antagonist, improves working memory (Hupalo and Berridge, 2016). Moreover, PFC CRF neuron activation inhibits working memory, an effect blocked by intra-mPFC CRF1 antagonism (Hupalo et al., 2019). Interestingly, mPFC CRF signaling decreased PFC, and to a lesser extent striatal, neuron task-related encoding. However, intra-mPFC administration of CRF does not modify sustained attention (Hupalo and Berridge, 2016), suggesting that CRF signaling in the PFC may differentially regulate different aspects of PFC-dependent cognition. Interestingly, chemogenetic activation of PFC CRF-expressing neurons impairs working memory, an effect blocked by systemic, but not intra-mPFC, administration of a CRF1 antagonist. The authors concluded that effects of CRF neuron activation is due to release of CRF in PFC terminal regions. This work establishes a role for PFC CRF systems in regulating cognitive function. Further, CRF-containing neurons in the PFC regulate motivated behavior under stress. Specifically, a subset of PFC CRF-containing interneurons is recruited in tail suspension test and ablation or inhibition of these neurons increase immobility time in mice (Chen P. et al., 2020). Interestingly, activation of CRF neurons promotes resilience. These results suggest CRF neurons may become engaged to promote adaptive behaviors to overcome stress rather than driving mal-adaptive behavior. In a mouse model of stress-induced depression, the ablation or antagonism of CRF1 receptors abolishes behavioral despair (Dedic et al., 2018; Deussing and Chen, 2018). Thus, the PFC CRF may control adaptive and/or mal-adaptive behaviors depending on the severity and/or duration of the stressor. Microinjection of CRF into the PFC increases anxiety-like behavior in the elevated plus-maze (EPM) in both acute and chronically stressed rats (Jaferi and Bhatnagar, 2007). CRF injection in the rat frontal cortex induces anxiogenic actions, but at high doses produces an anxiolytic-like effect (Zieba et al., 2008). Interestingly, the anxiolytic action of may be mediated by engagement of alpha-adrenergic signaling (Smialowska et al., 2021). CRF1 receptor in the PFC has been implicated in the emotional adaptation to stress (Uribe-Marino et al., 2016). Consistent with the CRF-5-HT interactions mentioned above, CRF/CRF1 transmission regulates anxiety-related behaviors through 5-HT2R signaling in the PFC (Magalhaes et al., 2010), indicating that the CRF system interacts in the PFC with other neurotransmitters such serotonin and norepinephrine to regulate anxiety-like behaviors. Collectively, these studies suggest that PFC CRF systems regulate PFC-dependent behaviors, including anxiety-like behavior and cognition.

There is a wide array of evidence that suggests that CRF is a viable target for the treatment of psychiatric disorders. Preclinical and postmortem studies show elevated CRF concentrations in patients diagnosed with major depressive disorder (Nemeroff et al., 1984; Raadsheer et al., 1995; Deussing and Chen, 2018). Furthermore, there is compelling evidence that the progression from recreational to compulsive drug use is driven by a shift in emotional and motivational homeostasis to an allostatic setpoint, resulting in a state of decreased reward function and increased stress responsivity. It is hypothesized that the CRF system plays a significant role in the negative emotional state and habitual drug-seeking in individuals with severe addiction (Silberman et al., 2009; Koob, 2010; Zorrilla et al., 2014). Based on the hypothesis that CRF system dysregulation contributes to negative affect, various clinical trials using CRF1 receptor antagonists have been completed, with conflicting results (Spierling and Zorrilla, 2017). A CRF1 receptor antagonist, NBI 30775/R121919, reduced depression and anxiety scores using patient and clinician ratings without impairing corticotropin and cortisol secretion in patients with MDD (Zobel et al., 2000). Further, a clinical trial in major depressive disorder patients reports that a non-peptidic CRF1 receptor antagonist reduces symptoms of anxiety and depression (Kehne and De Lombaert, 2002). In contrast, the CRF1 receptor antagonist CP-316,311 did not reduce depression score patients with MDD compared with placebo-treated controls (Binneman et al., 2008). The CRF1 receptor antagonist verucerfont (GSK561679) failed to reduce PTSD symptoms compared to placebo (Dunlop et al., 2014). Several factors may contribute to mixed reports, including but not limited to variation in CRF or CRF1 receptor genetic or protein expression, limited target engagement, therapeutic effects that may be only observed in certain conditions (e.g., acute stress and chronic stress), and/or biased signaling associated with different compounds that have been tested in clinical trials. These studies present a challenge to the field to evaluate molecules that modulate CRF signaling to promote therapeutic outcomes in clinical trials.

Although extensive research has been done on the CRF system in the frontal cortex, much remains unknown about its architecture and function in cortical circuits. It is unclear how CRF modulates the activity of the cortical circuitry via regulation of local circuit excitatory and inhibitory neurons and afferent inputs to the cortex. Despite extensive research on the role of the CRF system in regulating stress-related and anxiety-like behavior, little is known about the role of this system in regulating reward processing beyond the context of cognitive tasks. Given that PFC CRF neurons regulate encoding of working memory (Hupalo et al., 2019), it will be of interest to understand how CRF system shapes PFC activity to acute and chronic stressors and during reward processing. It is also unknown how CRF binding protein (CRFBP) influences the CRF system in the PFC. CRFBP was first postulated as a sequester of CRF, effectively reducing CRF concentration and receptor activity (Cortright et al., 1995). Further studies have revealed other actions of CRFBP. Specifically, CRFBP has been shown to have a facilitatory role of CRF-induced potentiation of NMDAR-mediated synaptic transmission in the ventral tegmental area (Ungless et al., 2003), function independently of the CRF receptor (Chan et al., 2000), and act as an escort protein to traffic CRF2a to the cell surface (Slater et al., 2016). CRFBP is expressed in GABAergic cells in the PFC (Ketchesin et al., 2017), whereas CRF and CRF1 receptor expression is primarily observed in glutamatergic cells. This raises the question of how CRF–CRFBP interactions may modulate activity of PFC circuitry during motivationally charged behaviors. Lastly, it is unclear whether urocortins influence prefrontal cortical circuits and behavior given that they differentially activate CRF1 and CRF2 and these receptors may differ in their anatomical location within the cortex.

### Cholecystokinin

Cholecystokinin (CCK) is a neuropeptide and gut hormone that belongs to the gastrin family and has various regulatory functions in the brain and gut. In the nervous system, CCK regulates learning and memory, nociception, homeostatic sensation, affective behavior, and drug-seeking behavior (Reisi et al., 2015). CCK peptides evoke downstream signaling pathways via CCK-A and CCK-B receptors, which signal through G_*q*_ protein to activate phospholipase Cβ and increase intracellular Ca^2+^ levels (Johnsen, 1998; Williams et al., 2002).

Radio-immune and *in situ* hybridization studies reveal CCK expression is abundant in the cerebral cortex (Beinfeld et al., 1981; Savasta et al., 1988; Ingram et al., 1989; You et al., 1993) and mainly expressed in cortical interneurons (Vanderhaeghen et al., 1981; McDonald, 1982; Hendry et al., 1983; Morino et al., 1994; Gallopin et al., 2006). Single cell reverse transcription polymerase chain reaction experiments indicate CCK mRNA is expressed in approximately 30–40% of GABAergic interneurons in the cortex (Gallopin et al., 2006). Moreover, CCK and CCK mRNA is expressed in both glutamatergic and GABAergic neurons (Radu et al., 2001). CCK-expressing interneurons predominantly display fast-spiking properties, with a smaller subset displaying non-fast-spiking properties (Nguyen et al., 2020). CCK-positive interneuron synaptic transmission is stronger onto intra-telencephalic (contralateral cortex-projecting PFC neurons) than PAG-projecting pyramidal cells (Liu et al., 2020), indicating that CCK-positive interneurons in the PFC impose inhibitory control of pyramidal output neurons based on output. CCK-positive neurons are also projection neurons. Intersectional genetic approaches reveal CCK-GABAergic cells are more predominant in higher order associative cortices, including both ventral and dorsal aspects of the mPFC, relative to PV-GABAergic cells (Whissell 2015). This suggests there is regional specialization of soma-targeting neurons that utilized CCK as a neuropeptide.

Despite advancements in understanding how CCK-expressing cells are embedded in cortical circuits and use fast inhibitory and excitatory amino acid neurotransmitters to regulate circuit function, there is less known about how these cells use CCK as a peptide transmitter. CCK-immunoreactivity is observed in inhibitory symmetric synapses in the cortex of rodents and non-human primates, suggesting release of CCK along with GABA within the cortex (Hendry et al., 1983). Furthermore, studies delineate that CCK-B receptors are extensively expressed in neocortical pyramidal neurons, and activation of these receptors by their endogenous agonist (CCK) depolarizes and evokes spiking of pyramidal cells (Gallopin et al., 2006). Consistent with this finding, CCK action through CCK-B receptors produce a long-lasting excitation of layer VI neocortical neurons via inhibition of a K^+^ leak current (Chung et al., 2009). Since layer VI pyramidal neurons densely innervate thalamic nuclei, these results imply that CCK modulates corticothalamic circuitry. Similar CCK-induced increases in pyramidal cell excitability have also been observed in hippocampal circuits (Dodd and Kelley 1981; Boden and Hill 1988). The capacity for CCK to regulate excitability of cells in cortical circuitry, may modify higher level processing and synaptic plasticity. For example, endogenous CCK is released in auditory cortex in response to high frequency stimulation and is necessary for long-term potentiation of excitatory transmission (Chen et al., 2019).

Interestingly, optogenetic inhibition of CCK positive interneurons in the PFC, impaired working memory retrieval in mice (Nguyen et al., 2020). However, it is unclear how the neuropeptide in this cell population may contribute to working memory. Notably, CCK expression and release may be modified by behavioral experiences. For instance, extracellular CCK levels as assessed by micro-dialysis are increased in the frontal cortex of rats in response to restraint stress, the anxiogenic drug yohimbine, and ether (Nevo et al., 1996). Further, rats exposed to a foot-shock stress paradigm show increased CCK-immunoreactivity in the PFC (Siegel et al., 1984). Arousal induced by saline injections is associated with a delayed increase in tissue CCK levels, an effect that is blocked by ketamine pretreatment. A CCK-releasing circuit from the entorhinal cortex to the auditory cortex is critical for associative aversive learning and experience-dependent plasticity (Chen et al., 2019), highlighting the importance of CCK transmission in shaping information processing within cortical circuits and associated behaviors. Additionally, activation of the CCK receptor B by endogenous CCK increases the time mice spend in the open arms of an Elevated Plus Maze behavioral paradigm (Ballaz et al., 2020). This study suggests that CCK and CCK-B receptor drive anxiolytic effects in mice, in addition to aversive learning. Collectively, these studies suggest that CCK, as a peptide transmitter in cortical circuits may be recruited during motivationally charged behaviors to impact circuit function and appropriate PFC-dependent behavior.

Despite the extensive research on CCK and the downstream effects of CCK-A and CCK-B receptor signaling, much remains unknown. It is unclear what the role of CCK originating from GABAergic interneurons versus excitatory neurons is in shaping information processing and behavior. It was previously established that CCK neurons in the amygdala play an important role in modulating fear and anxiety like behaviors, yet the mechanisms of action of CCK in frontal cortical circuits remains unclear (Truitt et al., 2009; Brown et al., 2014; Schmidt et al., 2014). Additionally, CCK has a high degree of homology to gastrin, which shares a common c-terminal sequence (Johnsen, 1998; Baldwin et al., 2010). Like CCK, gastrin signals via CCK-A and CCK-B receptors. In the nervous system, including the cortex, gastrin binds to CCK-B with high affinity (Johnsen, 1998). Immunohistochemistry studies note CCK- and gastrin-positive cells throughout the cortex (Vanderhaeghen et al., 1981), however, it is unclear if gastrin produces analogous effects to CCK, given its high affinity for the CCK-B receptor. Advancements in basic science will be pivotal for determining whether off-label use of CCK ligands in the pipeline for other indications may be considered for treatment of neuropsychiatric disorders. Modulation of CCK has shown anxiolytic effects, enhanced working memory, and influenced motivated behaviors. Thus, CCK serves not only as a promising therapeutic target for psychiatric disorders, but also gives rise to other areas of research.

### Somatostatin

The neuropeptide SST was first isolated from hypothalamus and identified as somatotropin-release inhibiting factor (SRIF) (Krulich et al., 1968; Brazeau et al., 1973). There are two active forms of SST derived from the pre-prosomatostatin peptide but differ in amino acid length: SST-14 and SST-28 (Brazeau et al., 1973; Esch et al., 1980; Pradayrol et al., 1980; Schally et al., 1980). Both SST peptides are expressed in the CNS, however the expression and cellular distribution of these two forms varies between cortical and subcortical brain regions (Epelbaum, 1986). SST is stored in large DCVs and released in a calcium-dependent manner (Tapia-Arancibia et al., 1989; Bonanno et al., 1991). SST release from cortical neurons driven by excitatory transmission (Song et al., 2021) and is potentiated by stimulation of other neuropeptides and neuromodulators, such as neurotensin, VIP, and dopamine (Robbins and Landon, 1985; Thal et al., 1986). SST release in in cortical slices has also been documented in response to optogenetic stimulation (Dao et al., 2019). SST release is under the inhibitory control of GABAB receptors, suggesting that SST release is gated by inhibitory neurons as well. Thus, SST release is under bi-directional control in cortical circuits, and is recruited in response to activity and neuromodulation.

Upon release from DCVs, SST binds to its cognate SST receptor (SSTR). Five SSTRs have been cloned and characterized: SSTR1–SSTR5. All SSTRs are G_*i/o*_-coupled GPCRs and bind both SST14 and SST-28 with high affinity. SSTR-1–4 exhibit higher binding affinity for SST-14 than SST-28, while SSTR-5 has greater selectivity for SST-28 (Reisine and Bell, 1995; Barnett, 2003). Expression of all five SSTRs has been demonstrated in the cortex, with SSTR1 and SSTR2 as the two most prominent SSTRs in the human and rat cerebral cortices (Dournaud et al., 1996; Bologna and Leroux, 2000). Immunohistochemical analysis of SSTR expression in the somatosensory cortex suggest that SSTRs are differentially localized to different layers (Lukomska et al., 2020). SST activation of SSTRs generally suppresses the release of hormone or neurotransmitter from target neurons by activating a G-protein signaling pathway that inhibits adenylate cyclase and calcium channels (Martel et al., 2012). Cortistatin (CST), a neuropeptide naturally expressed in the cortex, is another endogenous ligand that can bind to SSTR. CST has the same amino acid sequence at the receptor binding site as SST and studies show that CST can active all subtypes of SSTRs with nanomolar affinity to induce similar signaling consequences as SST (de Lecea, 2008; Song et al., 2021). Together, SST and CST act through SSTRs to enact G-protein signaling cascades that regulate neural function.

Somatostatin-expression has been identified in neurons throughout the mammalian brain. In the cerebral cortex, SST is expressed predominantly in a subgroup of GABAergic interneurons. SST-positive GABAergic neurons represent approximately 30% of the total cortical interneuron populations (Rudy et al., 2011; Urban-Ciecko and Barth, 2016). SST interneurons provide dendritic inhibition onto pyramidal neurons to regulate integration of excitatory inputs (Liguz-Lecznar et al., 2016). Further studies reveal SST neurons are heterogenous and show diverse properties in firing pattern, arborization, connectivity, and transcriptome profiles (Fishell and Rudy, 2011; Tasic et al., 2018; Naka et al., 2019) Using a unbiased, large-scale profiling method, Jiang et al. (2015) analyzed that cortical SST neurons consist of three subpopulations: low-threshold or irregular-spiking Martinotti neurons, fast spiking basket cells, and bitufted cells. Thus, multiple types of cells in the cortex have the potential capacity to release SST peptides in addition to GABA.

Electrophysiological studies have demonstrated that SST application results in heterogeneous effects on excitability cortical neurons, with excitation being the most prominent (Olpe et al., 1980; Delfs and Dichter, 1983). Increases in activity in response to SST have also been reported in the hippocampus (Mueller et al., 1986). IPSPs are also inhibited by SST application (Leresche et al., 2000). Recent work has started to dissect how SST may facilitate spiking. SST decreases excitatory synaptic transmission onto PV-expressing interneurons, but not pyramidal neurons, via a presynaptic mechanism (Song et al., 2020). Importantly, this effect was recapitulated by endogenous SST release evoked by optogenetic stimulation of SST neurons, providing a potential mechanism for principal neuron disinhibition. SST-immunoreactivity is observed near GABA release in presynaptic terminals that appose excitatory terminals (Kecskés et al., 2020; Song et al., 2020). This anatomical framework is consistent with a role of SST in regulating excitatory synaptic transmission via a presynaptic site of action. SST may also have direct post-synaptic actions on cortical pyramidal neurons. SST-14 and SST-28 enhance and decrease delayed-rectifier potassium currents in cultured cortical neurons, respectively (Wang et al., 1989). SST signaling via SSTR4 induces hyperpolarization of principal neurons has also been observed in the medial entorhinal cortex (Kecskés et al., 2020), an effect that is more robust in layer III/V than layer II neurons. This is consistent with a role of SSTR4 in retinal ganglion cells in inhibiting L-type Ca^2+^ channels, which enhance intrinsic excitability (Farrell et al., 2014). SST also hyperpolarizes principal neurons in the CA1 of the hippocampus (Mueller et al., 1986). Collectively, these data provide circuit-based mechanisms wherein SST release may regulate information processing in cortical circuits.

Since SST neuropeptide transmission has the potential to influence synaptic transmission and intrinsic excitability in cortical circuits, it is of no surprise that numerous studies have demonstrated that cortical SST interneurons play a critical role in sensory processing, motor control, cognition, and emotion. However, evidence linking SST peptides and their receptor system directly with these cortical functions is limited. Intraventricular (ICV) infusion of SST peptide in rats induced anxiolytic- and antidepressant-like effects in the EPM and forced swim test, respectively (Engin et al., 2008). The anxiolytic effect was recapitulated following ICV infusions of a selective SSTR2 receptor agonist, whereas the antidepressant-like effect was mimicked following infusions of either SSTR2 or SSTR3 agonists (Engin and Treit, 2009). Increasing evidence have shown that heightened brain SST level counteracts stress-induced ACTH, catecholamine, and CRF release, suggesting that SST suppresses stress-induced responses (Brown et al., 1984; Stengel and Tache, 2017). Similar roles of SST neurons in mediating working memory, fear conditioning, and enhancing circuit performance have also been described (Kim et al., 2016; Abbas et al., 2018), yet the role of the of SST neuropeptide in this process is unclear. In the olfactory system, infusion of a SSTR2 agonist into mouse main olfactory bulb (MOB) increases gamma oscillation, synaptic transmission, and enhances odor discrimination performances (Lepousez et al., 2010), suggesting that enhancement of circuit performance may be a feature of SST signaling. Recently, Song et al. (2020) found that SST application in the primary visual cortex (V1) improves visual discrimination in freely moving mice and enhances orientation selectivity of V1 neurons. Further, they demonstrated that SST improves visual perception by enhancing visual gain of V1 neurons via a reduction in excitatory synaptic transmission to PV fast-spiking interneurons but not to regular-spiking neurons (Song et al., 2020). Thus, SST peptide in cortical circuits regulates information processing to shape behaviors subserved by the cortex.

Somatostatin expression in the cortex is impacted in a plethora of neuropsychiatric disorders (Crowley and Kash, 2015; Song et al., 2021). A decrease in SST expression in the cortex is observed in neurodegenerative and psychiatric disorders such as Alzheimer’s disease (Davies et al., 1980; Kumar, 2005), Parkinson’s disease (Epelbaum, 1986; Iwasawa et al., 2019), Huntington’s disease (Rajput et al., 2011), major depressive disorder (Rubinow et al., 1985; Tripp et al., 2011; Lin and Sibille, 2015), bipolar disorder (Fung et al., 2014; Pantazopoulos et al., 2017), and schizophrenia (Reinikainen et al., 1990; Hoftman et al., 2015). Interestingly, SST neuron activity is necessary for the antidepressant effects of scopolamine (Wohleb et al., 2016), suggesting that decreased activity of SST neurons potentially drives depressive-like behavior. Given preclinical evidence that the brain SST system has profound anxiolytic and anti-depression effects (Crowley and Kash, 2015; Song et al., 2021), this system has therapeutic potential for treating neuropsychiatric disorders. Several synthetic SST analogs have been developed for clinical treatment of endocrine diseases, digestive diseases, and carcinogenic tumor (Gomes-Porras et al., 2020). However, no drugs targeting the SST system are approved or under investigation in clinical trials for the treatment of neurodegenerative and psychiatric disorders, mainly because current SST analogs cannot penetrate the blood–brain barrier (Lamberts et al., 1996). New drug packaging and delivery approaches, as well as gene therapy techniques, will facilitate the development of CNS-targeted SST drugs and genetic treatments.

Despite extensive research on the action of and therapeutic potential of SST and SSTRs, much remains unknown about the role of this system in shaping cortical circuits. Recently, activity dependent cortical SST release via optogenetic stimulation was used providing a potential platform to probe the influence of circuit manipulations and/or behavior on SST release (Dao et al., 2019). Cortical SST neurons are diverse in terms of their molecular profiles, anatomical features, and electrophysiological properties. Considering the distinct firing patterns of SST subpopulations, it is imperative to understand how SST release is fine-tuned by different forms of neuronal activity of diverse types of SST neurons. Furthermore, whether SST acts on different targets and cell compartments differently due to its layer-specific arborization is an unknown. Additionally, other neuron peptides, such as NPY and Dyn, are co-expressed with SST in subpopulations of interneurons (Sohn et al., 2014; Smith et al., 2019) but it is unknown whether SST can be co-released with other peptides from the same site, and how different neuropeptides released from the same neuron shapes cortical signaling processing. Further, CST is also predominantly expressed in cortical GABAergic neurons and often co-expressed with SST (Smith et al., 2019). Additional investigations are needed to understand whether CST and SST peptides that target the same receptors work synergistically, competitively, and/or in parallel in cortex. In summary, the cortical SST system influences cortical information processing. Activation of this system induces anxiolytic and anti-depression effects, and deficits in this system are observed in neurodegenerative and neuropsychiatric disorders. Understanding the nuanced mechanisms by which the cortical SST system shapes cortical information processing and control of behavior will significantly advance the development of new therapeutic treatments for neurological disorders.

### Neuropeptide Y

First isolated in 1982 from the porcine hypothalamus, NPY is one of the most widely expressed neuropeptides in the central and peripheral nervous systems (Tatemoto, 1982). NPY consists of 36-amino acid residues and belongs to the family of pancreatic hormone polypeptides (PP). NPY is derived from its 97-amino-acid precursor peptide, pre-pro NPY, into a mature 36 amino-acid NPY1-36, which is cleaved by the enzyme dipeptidyl peptidase 4 to produce NPY3-36 (Aizawa-Abe et al., 2000; Robich et al., 2010; Dvorakova et al., 2014). NPY has widespread cardiovascular, immune, metabolic, and reproductive functions in the peripheral nervous system and regulates neural function underlying feeding, stress-, and addiction-related behaviors (Heilig, 2004; Hirsch and Zukowska, 2012).

Expression of NPY is well conserved across species, including the rat, mouse, and humans across multiple brain regions, such as the hypothalamus, cortical areas, the septum, hippocampus, olfactory bulb, and striatum (Dumont et al., 1992). The widespread effects of NPY are mediated by a family of rhodopsin-like GPCRs: Y1, Y2, Y4, Y5, and Y6. The Y6 receptor subtype is functional in the mouse and rabbit but not in humans and other primates while being absent in the rat (Larhammar and Salaneck, 2004). Notably, there are mixed reports regarding the existence of a Y3 receptor subtype, given that attempts at cloning this receptor have not been successful (Gehlert, 1998; Lee and Miller, 1998; Pedragosa Badia et al., 2013). NPY receptors are predominantly located post-synaptically except for the Y2 receptor, which is pre-synaptically located (Decressac and Barker, 2012). Activating NPY receptors may initiate multiple signaling cascades associated with G_*i/o*_ proteins (McQuiston and Colmers, 1996; Sah and Geracioti, 2013). Altogether, these studies highlight the need for studying the neuromodulatory role of NPY in regulating cortical physiology and behavior.

Neuropeptide Y is found in layers I, II, V, and VI of the human cortex (Adrian et al., 1983; Chan-Palay et al., 1985; Van Reeth et al., 1987) and highly colocalized with GABA and SST (Sawchenko et al., 1985; Aoki and Pickel, 1989). Although expressed by both pyramidal cells and interneurons, NPY is predominately found in interneurons (Dawbarn et al., 1984; Hendry et al., 1984; Karagiannis et al., 2009) and can be categorized into three main classes: neuroglia form-like neurons, Martinotti-like, and parvalbumin-positive basket cells (Karagiannis et al., 2009). Together, NPY and cognate receptors modulate synaptic excitability and function in cortical areas (Dumont et al., 1992). Specifically, NPY has been shown to reduce the EPSCs and decrease AMPA receptor-mediated glutamatergic neurotransmission onto neocortical pyramidal neurons (McQuiston and Colmers, 1996; Bacci et al., 2002), an effect that has also been observed in other brain regions like the hippocampus (McQuiston and Colmers, 1996; Qian et al., 1997) but not the amygdala (Molosh et al., 2013). Furthermore, NPY also regulates GABAergic neurotransmission onto pyramidal neurons in the neocortex (Bacci et al., 2002), suprachiasmatic nucleus (Chen and Van Den Pol, 1996), thalamus (Sun et al., 2001), but not the hippocampus (Klapstein and Colmers, 1993). Specifically, Bacci et al. (2002) found that NPY produces delayed and long-lasting decreases in EPSCs and increases in IPSCs in pyramidal neocortical neurons, an effect likely due to Ca^2+^-dependent enhancement of presynaptic GABA release. Furthermore, it was found that NPY also decreased IPSCs in GABAergic interneurons, suggesting NPY may inhibit pyramidal neurons by disinhibition of GABAergic interneurons. Consistent with the observations that NPY decreases neuronal excitability, functionally, NPY is known to have antiepileptic effects (for a review, see Vezzani and Sperk, 2004) and to provide neurons with protection against cytotoxicity by decreasing microglial reactivity, the release of microglial bioactive factors, NMDA currents and excessive Ca^2+^ entry into neurons (Li et al., 2014). Altogether, these studies suggest that NPY decreases the excitability of cortical circuits.

Neuropeptide Y signaling in the frontal cortex has been linked to affect-related disorders, highlighting the importance of characterizing the neuromodulatory role of NPY for psychiatry. On one hand, reduced NPY activity in cortical circuits has been associated with a plethora psychiatric disorders including anxiety, depression, and PTSD in both preclinical and clinical studies (Zhou et al., 2008; Mickey et al., 2011; Cohen et al., 2012; Melas et al., 2012, 2013; Sah and Geracioti, 2013). Previous work has found low levels of NPY expression in cortical brain regions of humans with a history of depression and death by suicide (Widdowson et al., 1992). Consistent with clinical work, preclinical studies report decreased cortical NPY, and Y1 receptor gene and protein levels expression in animal models of depression (Husum et al., 2001; Jimenez-Vasquez et al., 2007). Indeed, NPY and Y1 receptor agonists have been shown to decrease anxiety-like behavior, fear-suppressed food reinforcement, contextual fear, and social avoidance in rodents (Heilig et al., 1989; Sajdyk et al., 1999; Comeras et al., 2021). However, other reports have shown that activation of the NPY system in the infralimbic cortex impairs the retrieval of extinction in rats (Vollmer et al., 2016). Therefore, NPY-mediated inhibition of cortical circuits may also underlie deficits in fear extinction. Consistent with these findings, a clinical report found a link between impaired recall of extinction memory and reduced vmPFC activation in patients with PTSD (Milad et al., 2009). Together, these studies suggest that NPY modulation of cortical function underlies the etiology affect-related disorders.

Despite a breadth of studies demonstrating that NPY influences alcohol use disorder a few have suggested a link between cortical NPY signaling and alcohol use disorder (Mayfield et al., 2002). Alcohol preferring rats have low levels of NPY in cortical structures (Ehlers et al., 1998) and alcohol withdrawal produces significant reductions in NPY protein in several brain regions, including layers IV and V of the frontal cortex in the rat brain (Roy and Pandey, 2002). Additionally, rats fed an ethanol diet show reduction in NPY-immunoreactivity in the cortex (Bison and Crews, 2003). Previous work suggests that the ability of NPY to decrease alcohol consumption may be in part due to its ability to relieve alcohol withdrawal-induced anxiety (Thorsell and Mathé, 2017). Together, these results suggest that NPY-mediated inhibition of cortical circuits may play a role in the symptomatology observed in mood-and alcohol-related disorders and highlight the potential of NPY-based therapeutics for mental illnesses. Furthermore, the clinical relevance of the NPY system is evident in the clinical interest of NPY-based pharmacotherapies for treating numerous conditions. For example, a recent study suggests that NPY may be an effective treatment for anxiety in patients who have PTSD (Sayed et al., 2018) and intranasally administered NPY may produce rapid antidepressant effects (Mathé et al., 2020). These studies highlight a rich potential in the neuromodulatory role of NPY for the development of pharmacotherapies for treating psychiatric conditions such as anxiety disorders and depression.

In summary, extensive work demonstrates that NPY is a highly conserved neuropeptide involved in disease pathophysiology in the brain. While previous work demonstrates the potential of NPY-based pharmacotherapies for treating psychiatric illnesses, further work is needed to understand the functional effects of NPY signaling and its implications for brain diseases. For example, it is presently known that NPY is expressed within diverse interneuron populations that subserve distinct cortical and behavioral functions (Karagiannis et al., 2009; Smith et al., 2019). However, the role of NPY release from these different interneuron populations on cortical information processing and behavior is unknown. Moreover, NPY is also expressed in glutamatergic neurons of the PFC and it is unclear whether these cell populations release this peptide locally or in distal regions to influence behavior. Lastly, it is unclear whether NPY originating from interneurons and excitatory neurons may cooperate to more efficiently activate all pools of NPY receptor or whether NPY originating from interneurons would bind to sets of NPY receptors that are not accessible to NPY released from excitatory neurons. Understanding how NPY shapes PFC activity will help understand the role of NPY the NPY system in driving behavior under normal and pathological conditions.

### Vasoactive Intestinal Peptide

Vasoactive intestinal peptide is a peptide and member of the glucagon/secretin superfamily that signals through VIP receptors 1 and 2 (VPACR 1 and 2). VIP receptors are GPCRs that signal via the G_*s*_ signaling pathway. The VIP precursor gene, pre-proVIP, encodes for VIP as well as the peptide histamine isoleucine (PHI) and its human form, peptide histidine methionine (PHM), that both have a lower binding affinity for both VPACRs (Rangon et al., 2005; Igarashi et al., 2011). Different concentrations of VIP may confer differential activation of VPACR 1 and 2. For instance, lower concentrations of VIP (approximately 1 nM) activate VPACR 1/2 and pituitary adenylate cyclase receptor (PAC1-R) in the hippocampus leading to increased NMDA excitatory postsynaptic current amplitudes of CA1 hippocampal neurons (Yang et al., 2009). In contrast, larger concentrations of VIP (100 nM) act solely on the PAC1-R in the hippocampus and are dependent on an increase in Ca^2+^ intracellular levels. Collectively, VIP can regulate cortical function via interactions with VPACRs within cortical circuits.

In the PFC, VIP-positive interneurons are predominately expressed in L1b GABAergic cells expressing the ionotropic serotonin receptor 5HT3a (5HT3aR), as well as cholinergic cells (Tremblay et al., 2016; Anastasiades et al., 2021). Of the 30% of interneurons in the PFC that have 5HT3aR, 40% contain VIP (Tremblay et al., 2016). There are two major subpopulations of VIP interneurons: bipolar and multipolar. Bipolar VIP interneurons are in cortical layers II–VI and heavily concentrated in L2/3 and multipolar VIP cells are populated in the borders of L1/2 and deeper layers (Tremblay et al., 2016). In the cerebral cortex, VIP is radially oriented in bipolar interneurons with approximately 30% co-localization with GABA and 80% colocalization with acetylcholine (Magistretti, 1990). The mediodorsal thalamus drives VIP-positive interneurons localized in L1b to mediate cortical disinhibition via inhibition of SST-positive cells in L2/3 (Anastasiades et al., 2021). Accordingly, VIP contributes to a disinhibitory microcircuits (Anastasiades et al., 2021). Interestingly, VIP enhances the GABA-mediated inhibition of somatosensory neuron activity (Sessler et al., 1991). It is currently unclear clear how VIP peptide transmission coordinates with GABA-mediated inhibition. Furthermore, although VIP interneurons operate locally in cortical columns within the primary sensory cortex, it is unknown whether this same columnar organization holds true for higher-order cortices, such as the PFC species as functional columnar structures have been delineated in primary sensory cortices and differ across species (Fox, 2018).

Cortical modulation of VIP release is regulated by a variety of ion channels and neurotransmitters. In the cortex, Martin and Magistretti (1989a) revealed that K^+^-stimulated VIP release is Ca^2+^-dependent. In a series of experiments, they discovered that Ni^2+^, but not Cd^2+^, nifedipine, diltiazem, or ω-conotoxin, inhibited K^+^-evoked release of VIP, implicating T-type, and not L- and N-type, Ca^2+^ channels (Martin and Magistretti, 1989a). These studies suggest that DCVs containing VIP may recruit different pools of voltage-gated calcium channels, and hence different calcium sources, to trigger exocytosis, relative to vesicles containing fast neurotransmitters. Consistent with localization of VIP-positive neurons to superficial layers of the cortex, *in vivo* electrical stimulation of cortical superficial layers evoke VIP release ipsilateral but not contralateral to the stimulation site (Wang et al., 1985). VIP release is under the control of excitation and inhibition. Glutamate and kainic acid, as well as disinhibition with GABA antagonism, increase VIP release in cortical slices (Wang et al., 1986; Magistretti, 1990). Furthermore, VIP release is also sensitive to other neuromodulators known to impact cortical circuit dynamics, including norepinephrine, which decreased basal rates of VIP release (Wang et al., 1986). Conversely, carbachol, a cholinergic agonist increased the spontaneous release of cortical VIP (Magistretti, 1990). This is consistent with studies demonstrating that VIP neurons are recruited by external sources of acetylcholine from regions such as the forebrain (Letzkus et al., 2011). Moreover, since VIP is co-expressed with choline acetyltransferase (ChAT) and non-VIP ChAT neurons are found in cortical layer I, acetylcholine derived for local cortical sources may also regulate release of VIP. The majority of VIP-positive/ChAT-positive interneurons in layer I co-release acetylcholine and GABA. However, in the mPFC, ChAT-containing, VIP-lacking cells also release acetylcholine in L1 (Granger et al., 2020). Contradictory results suggest that endogenous opioid systems may regulate VIP release as topical super fusion of MOR, DOR, and KOR agonists had no effect on the spontaneous release of cortical VIP. Interestingly, naloxone, a competitive opioid antagonist, increased VIP efflux. These results suggest that basal opioid tone may be maximally recruited, occluding agonist effects. Some caveats between the effects of *in vivo* versus *in vitro* administration of DADL, an opioid peptide, on cortical VIP-LI release has been explored. Wang et al., discovered that applying DADL suppressed K^+^-stimulated cortical VIP-LI release. In contrast, Micevychi et al. (1984), showed that *in vitro*, cortical application of DADL, had no effect on potassium-evoked VIP-LI release. Lastly, reticular formation stimulation evoked VIP release *in vivo*, while anesthesia suppresses it (Micevychi et al., 1984), suggesting that VIP release may be linked to arousal. Collectively, these studies provide a framework for future studies to dissect the molecular machinery that underlies the release of VIP.

Vasoactive intestinal peptide interneurons within the PFC modulate behavior either through disinhibition or excitation of cortical neurons. Lee et al. (2013), found the primary vibrissa motor cortex (vM1) strongly projects to VIP interneurons that suppress SST cells. This hyperpolarization of the SST cells by the VIP interneurons allows for voluntary whisking behavior (Lee et al., 2013). Further, PFC ChAT VIP interneurons play a role in maintaining attention as ChAT-VIP interneurons directly excite neurons within different layers of the PFC with fast, cholinergic synaptic transmission (Obermayer et al., 2019). VIP-mediated disinhibition that alleviates dendritic inhibition in cortical pyramidal cells and is also associated with fear learning (Letzkus et al., 2011), amplifies visual processing (Keller et al., 2020). It was discovered that VIP disinhibition of ventral hippocampal inputs to the mPFC cause a decrease in prefrontal responses that results in less open-arm avoidance in the elevated plus maze (Lee et al., 2019). Additionally, a decrease in GCaMP signaling during exploration predicted approaches to the open arms and concluded this circuit helped predict exploratory behavior rather than avoidance (Lee et al., 2019). Together, it is evident that VIP regulates various neural functions.

Although, information regarding the function of VIP interneurons in cortical processing and behavior has continued to accrue in recent years, there is still little known about the effects of VIP release in the PFC. There is limited understanding of the effects of VIP on synaptic transmission or intrinsic excitability of distinct cortical cell types. Given that VIP neurons have unique localization and connectivity patterns within cortical circuits, it is of interest to determine how peptidergic VIP transmission may impact inhibitory interneurons that VIP neurons target for disinhibition versus VIP-sensitive excitatory and inhibitory neurons. VIP has also been implicated in mediating glycogenolysis (Martin and Magistretti, 1989b). VIP’s action increases cAMP levels to convert glycogen to glucose 1-phosphate, shifting energy homeostasis through interactions with neurons and glia (Magistretti et al., 1986; Magistretti, 1990). Interestingly, PHI/M, have shown a similar effect of cortical glycogenesis, with lower potencies, implying a role for this mechanism within peptides that share significant similar VIP homology (Magistretti et al., 1986; Magistretti, 1990). Overall, these studies suggest VIP regulates energy substrate accessibility within cortical circuits. VIP’s contribution to energy homeostasis in the cortex may further be supported by its role in vasodilation. The role of VIP’s effects on cortical microvessels has yet to determined. Further, it is unclear if VIP arising cortically released pools or circulating cerebrospinal fluid near superficial layers where VIP neurons reside contribute to cortical vasodilation (Magistretti, 1990). A recent study demonstrated that optogenetic VIP interneuron stimulation resulted in vasodilation and this effect was not due to release of acetylcholine (Granger et al., 2016, 2020), which raises the possibility that VIP or another substance released from this interneuron population may influence vasodilation. Therefore, studies determining the role of VIP in vasodilation, glycogenolysis, and effects within different cortical layers will provide further knowledge about the neuromodulatory actions of this peptide.

## Novel Approaches to Study Neuropeptides

**FIGURE 2:**
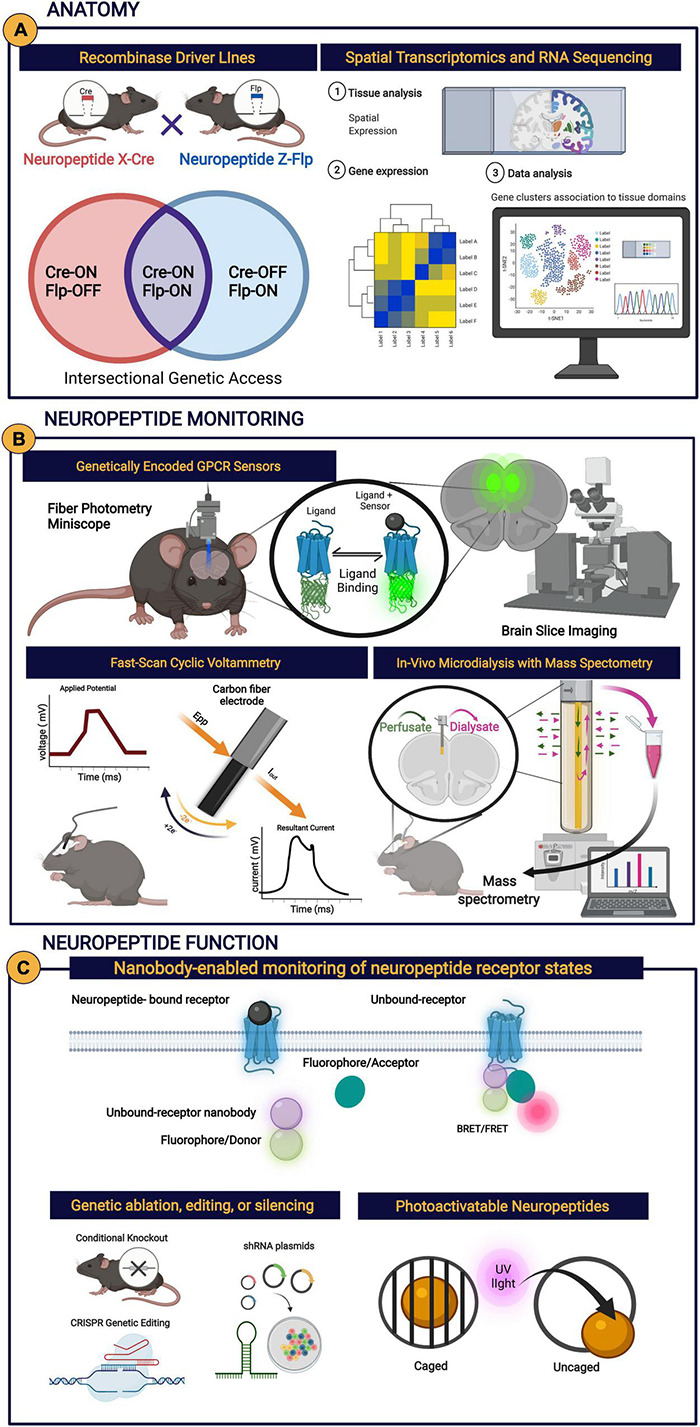
**(A)** Anatomical methods in the study of neuropeptides include recombinase driver lines used to gain genetic access to neuropeptide or receptor-expressing cells, spatial transcriptomics, and RNA sequencing. **(B)** Neuropeptide monitoring methods include genetically encoded GPCR sensors in conjunction with recording methods (e.g., fiber photometry and miniscope), fast-scan cyclic voltammetry (inspired by Roberts and Sombers, 2018), and *in vivo* microdialysis with mass spectrometry. **(C)** Methods to study neuropeptide function include nanobody enabled monitoring of neuropeptide receptor states (inspired by Che et al., 2020), genetic modifications (e.g., genetic ablation, editing, or slicing), and the use of photoactivatable neuropeptides. Any of the aforementioned novel approaches can be implemented with established methodologies, such as electrophysiology.

Although it is assumed that neuropeptide release modulates PFC processing, the cellular and circuit-based framework by which this occurs is in large part lacking. Many unknowns regarding the consequences of neuropeptide release and subsequent GPCR action in cortical circuits remain. Much of this gap in knowledge stems from limited tools available to dissect the function of neuropeptide transmission in behavior and cortical function. Fortunately, in recent years there has been a rapid expansion of approaches available to study neuropeptide transmission. Here, we address various novel tools that have been developed to facilitate addressing these unknowns (Figure 2).

### Dissecting Peptide and Receptor Anatomy

To obtain a deep understanding of the role of neuropeptidergic transmission in shaping cortical circuit function, it is imperative to understand how neuropeptide-expressing cells and the incoming inputs and local circuit cells sense this information via neuropeptide receptors. To investigate the anatomy of cells expressing various neuropeptides and cognate receptors, transgenic and viral approaches are increasingly becoming indispensable tools (Figure 2A). The ever-increasing availability of Cre-driver lines, that express Cre-recombinases in the presence of a selected neuropeptide or neuropeptide receptor, grant genetic access to cell types that release neuropeptides and contain neuropeptide receptors. Specifically, viruses expressing fluorophores in a Cre-dependent manner can be used in tandem with Cre-driver lines to visualize distribution of cells that putatively express specific neuropeptides or cognate receptors within cortical circuits, as well as their terminals throughout the brain. The advent of intersectional genetic strategies will permit researchers to gain genetic access to sub-populations of neuropeptide and/or GPCR-expressing cells (Fenno et al., 2014, 2020). This is of particular relevance given that neuropeptides and their cognate GPCRs are often expressed on different types of molecularly defined cells. In cases where validated antibodies or radiolabeled/fluorescent ligand exists it is possible to integrate these approaches with aforementioned viral and genetic approaches to dissect how neuropeptides or GPCRs embed themselves into local and long-range circuits. There are various important considerations and potential pitfalls when using genetic and viral approaches that must be addressed with appropriate controls and validation studies, including but not limited to, ectopic expression observed with germline or developmental expression of the recombinase that traces lineage cells that may transiently have neuropeptide or receptor promoter activity, incomplete genetic penetrance to cell types of interest, or Cre-independent transgene expression (Song and Palmiter, 2018; Botterill et al., 2021). Moreover, recombination of Cre or Flpo-dependent transgenes inherently is binary and does not reflect variations in neuromodulator expression levels. This is of importance when working with a new genetic line, virus, or even different brain regions. Additionally, the ever-expanding published single-cell RNA-seq datasets providing transcriptional profiles of individual cortical cell types and the neuropeptides and GPCRs found therein (Tasic et al., 2018; Smith et al., 2019; Krienen et al., 2020), will be useful resources for developing specific hypotheses relating to neuropeptide action in PFC circuits. Spatial transcriptomics including multiplexed *in situ* hybridization, *in situ* sequencing, and spatial barcoding will be able capture the spatial distribution of various cell populations expressing different neuropeptides and cognate receptors and provide an anatomical context to substantiate scRNA-seq studies (Longo et al., 2021). With increased capabilities to look at many neuropeptides and GPCRs, spatial transcriptomics will begin to unravel the “neuropeptidome” of cortical circuits (Smith et al., 2019). It should be noted that expression of mRNA does not confer functional neuropeptide or GPCR protein expression, highlighting caveats of these aforementioned approaches. Moreover, scRNA-seq experiments may also fail to identify neuropeptide or receptors whose mRNA is expressed in low abundance or localized away from the peri-nuclear space/soma, but is still of physiological relevance. Recently, mice expressing fluorescently labeled opioid receptors have been developed, which allows for mapping of receptors within sub-compartments or projections in defined cell types when coupled with anatomical tracing methods or when molecularly defined cells are tagged (Ehrlich et al., 2019; Chen C. et al., 2020). Careful characterization of novel lines with tagged neuropeptide receptors is necessary to ensure that expression or function of the targeted molecule is not impacted. For example, a thorough characterization of KOR-tdTomato fusion protein mice demonstrated that these mice have increase KOR mRNA expression and binding relative to controls (Chen C. et al., 2020), highlighting the importance of validation experiments and associated considerations as was done in the former study. Notwithstanding this approach will be useful for elucidating endogenous neuropeptide receptor localization and trafficking. Taken together, when used in tandem with physiological, biochemical, and functional anatomical approaches, these approaches provide valuable insight to neuropeptide mRNA expression in diverse cell types and anatomical architecture within tissues.

### Monitoring Neuropeptide Release

Historically, studies examining peptide release have been limited to acute brain slices or synaptosomal preparations where radioimmunoassays and ELISAs have been used to monitor peptide levels in the media. At best, microdialysis procedures have been used to measure fluctuations in extracellular neuropeptide levels in behaving animals. Although these techniques have led to significant advancements in the field, they have low temporal and spatial resolution or are restricted to *in vitro* conditions. To address these limitations, novel techniques with increased sampling or analytical sensitivity have arisen (Figure 2B). Specifically, fluorescent neuromodulator sensors (Wang et al., 2018; Patriarchi et al., 2019; Peng et al., 2020; Dong et al., 2021; Duffet et al., 2022) provide a means to detect fluctuations in neuropeptide release presence in real time in awake, behaving animals and map sub-cellular sites of neuropeptide release when coupled to high resolution live cell/acute brain slice image. A caveat of genetically encoded fluorescent sensors is that detection of events may be limited to large fluctuations in neuropeptide/receptor activity if the affinity of the neuropeptide for the sensor is not sufficiently high. Additionally, sensors with a very high affinity may display decreased dynamic range or buffer endogenous neuropeptides from acting on their cognate receptors. Lastly, since neuropeptide receptors may bind more than one neuropeptide with high affinity, including unidentified “off-target” actions, it is possible that fluorescent receptor sensor activity may not reflect activity of the neuropeptide of interest. Moreover, electrochemical sensors capitalizing on oxidation/reduction of Tyr in opioid peptides have been employed to monitor putative enkephalin (Roberts and Sombers, 2018). Appropriate controls demonstrating that signals can be detected from endogenously released neuropeptides and the absence of signal upon genetic ablation or inhibition of neuropeptide-expressing neurons will be essential in elucidating the substrates driving fluorescent or electrochemical sensor activity. Neuromodulator receptor-based fluorescent sensors will be useful not only for monitoring neuropeptide transmission in awake-behaving mice but will also be useful for understanding how neuropeptide transmission fits within nuanced architecture and layering of PFC circuits in acute slices and large fields of view *in vivo* with one or two photon imaging. Further, with increasing palettes of genetically encoded calcium and neurotransmitter sensors, future studies will be able to examine how neuropeptide dynamics fluctuate in relation to neuronal activity or other neurotransmitters. This will provide a comprehensive picture of how sensor activity relates to neuropeptide producing cells or receptor expressing cells. *In vivo* microdialysis coupled with mass spectrometry allow for simultaneous detection of various extracellular neuropeptides in addition to fast neurotransmitters and monoamines (DiFeliceantonio et al., 2012; Kennedy, 2013; Al-Hasani et al., 2018). As analytical approaches improve, detection of various species of neuropeptides and their metabolic byproducts will provide insight to the actual molecules present in the extra-cellular space and their metabolism. An added benefit is that microdialysis allows for local drug infusion at the sampling site permitting functional pharmacological or chemogenetic studies to be easily integrated. Ultimately, the development of novel techniques to monitor neuropeptide release will provide a platform to monitor cortical neuropeptide dynamics in freely moving behaving animals and closely link peptide dynamics to discrete aspects of behavior in well-designed studies.

### Mapping Receptor Function Within Circuits

Although the presence of GPCRs within cortical circuits could previously be identified with histological and *in situ* hybridization approaches, these approaches do not lend information to GPCR action and kinetics and physiological effects post neuropeptide binding. To identify how neuropeptide GPCRs regulate circuit function, photoactivatable neuropeptides, nanobodies, and advanced pharmacological and immunohistochemical tools are a few of the techniques used to date (Figure 2C). Advancements aimed at increasing specificity and efficacy of pharmacological tools, such as agonists and antagonists of neuropeptide targets, will facilitate work aimed at uncovering the role of neuropeptide action in the PFC. Cell-specific pharmacological approaches are rapidly developing that will aid in the dissection of neuropeptide control of cortical circuitry (Yang et al., 2015; Shields et al., 2017). These approaches provide a significant advantage over conventional pharmacological approaches, which do not discriminate between receptors in distinct circuit elements, in that they provide a means to concentrate or permit the selective gating of ligands to molecularly defined neurons or based on connectivity. Photoactivatable neuropeptides provide a means to determine the kinetics of neuropeptide and GPCR signaling as well as map sub-cellular distribution (Yang et al., 2015). When coupled to multi-photon or focal one-photon uncaging they also uncover spatiotemporally precise actions of GPCRs in distinct compartments of neurons. Genetic methods such as knock-in mouse lines expressing GPCRs fused with fluorescent markers reveal internalization of receptors following behavioral or pharmacological paradigms (Ehrlich et al., 2019; Chen C. et al., 2020). Furthermore, conditional knock-out of neuropeptide or receptor genes from cell populations using Cre-LoxP systems (Kim et al., 2018), short hairpin RNA (Moore et al., 2010), or CRISPR-Cas9 (Ran et al., 2013) will allow manipulation of peptide and receptor expression within cortical circuits. Given that neuropeptides are likely embedded into distinct cell types within PFC circuits that may differ based on molecular definition or connectivity, then expanding availability of recombinase driver lines or complementation of viral approaches will permit for dissection of neuropeptides and their receptors in specific cell types or pathways into or out of the PFC. Finally, changes in GPCR confirmational states in response to activation or deactivation can be studied with various techniques. For instance, nanobodies also can reveal GPCR confirmational states. For example, nanobodies that stabilize distinct ligand-delimited GPCR conformations can report real-time ligand-stabilized GPCR states (Che et al., 2020) and genetically encoded biosensors derived from specific nanobodies can provide precise spatial and temporal resolution of GPCR activation and deactivation (Stoeber et al., 2018). It has been shown that agonist binding induces KOR phosphorylation (Appleyard et al., 1997; Li et al., 2002) and immunohistochemistry can be used to report receptor phosphorylation as a measure of neuropeptide-mediated receptor activation (McLaughlin et al., 2004; Lemos et al., 2011; Abraham et al., 2021).

Collectively, there has been a rapid expansion of tools to dissect the function of neuropeptides. Ideal studies would use the most appropriate tools needed to address specific questions of how neuropeptides and their receptors relate to circuit function and/or behavior, keeping in mind strengths and caveats associated with each approach when designing studies and selecting tools to employ. In practice, dissection of the role of neuropeptides and their receptors in PFC circuits will require the use of multiple converging, complementary approaches, and inclusion of controls and validation studies, where appropriate. When coupled to cutting-edge *ex vivo* and *in vivo* electrophysiological, anatomical, *in vivo* imaging, and well-designed behavioral studies, these ensembles of emerging tools will be crucial for significant advances in our understanding the role of neuropeptides in intercellular communication in cortical circuits.

## Conclusion

In this review we provide a comprehensive examination and discussion of the literature on neuropeptide modulation of cortical circuitry, with an emphasis on the PFC. To date, knowledge of neuropeptide action is largely limited to subcortical brain structures. Expanding the field’s knowledge on neuropeptide action in the PFC and how neuropeptide systems regulate information processing in cortical circuits provides paths to develop therapeutic targets for the treatment of neuropsychiatric disorders. Further, increased knowledge PFC organization and function will provide a greater context for future findings on neuropeptide function. Together, investigating PFC networks and neuropeptide regulation of those circuits while capitalizing on novel approaches will help elucidate contributing factors in neuropsychiatric disorders and novel treatments for these disorders.

## Author Contributions

All authors wrote, edited, and approved the final manuscript.

## Conflict of Interest

The authors declare that the research was conducted in the absence of any commercial or financial relationships that could be construed as a potential conflict of interest.

## Publisher’s Note

All claims expressed in this article are solely those of the authors and do not necessarily represent those of their affiliated organizations, or those of the publisher, the editors and the reviewers. Any product that may be evaluated in this article, or claim that may be made by its manufacturer, is not guaranteed or endorsed by the publisher.

## References

[B1] Abate P. Reyes-Guzman A. C. Hernandez-Fonseca K. Mendez M. (2017). Prenatal ethanol exposure modifies locomotor activity and induces selective changes in Met-enk expression in adolescent rats. *Neuropeptides* 62 45–56. 10.1016/j.npep.2016.11.006 27889070

[B2] Abbas A. I. Sundiang M. J. M. Henoch B. Morton M. P. Bolkan S. S. Park A. J. (2018). Somatostatin interneurons facilitate hippocampal-prefrontal synchrony and prefrontal spatial encoding. *Neuron* 100 926.e3–939.e3. 10.1016/j.neuron.2018.09.029 30318409 PMC6262834

[B3] Abraham A. D. Casello S. M. Schattauer S. S. Wong B. A. Mizuno G. O. Mahe K. (2021). Release of endogenous dynorphin opioids in the prefrontal cortex disrupts cognition. *Neuropsychopharmacology* 46 2330–2339. 10.1038/s41386-021-01168-2 34545197 PMC8580977

[B4] Adesnik H. Naka A. (2018). Cracking the function of layers in the sensory cortex. *Neuron* 100 1028–1043. 10.1016/j.neuron.2018.10.032 30521778 PMC6342189

[B5] Adrian T. Allen J. Bloom S. Ghatei M. Rossor M. Roberts G. (1983). Neuropeptide Y distribution in human brain. *Nature* 306 584–586. 6358901 10.1038/306584a0

[B6] Aizawa-Abe M. Ogawa Y. Masuzaki H. Ebihara K. Satoh N. Iwai H. (2000). Pathophysiological role of leptin in obesity-related hypertension. *J. Clin. Investig.* 105 1243–1252. 10.1172/JCI8341 10791999 PMC315441

[B7] Al-Hasani R. Wong J. T. Mabrouk O. S. McCall J. G. Schmitz G. P. Porter-Stransky K. A. (2018). In vivo detection of optically-evoked opioid peptide release. *eLife* 7:e36520. 10.7554/eLife.36520 30175957 PMC6135606

[B8] Alvarez-Bolado G. Fairen A. Douglass J. Naranjo J. R. (1990). Expression of the prodynorphin gene in the developing and adult cerebral cortex of the rat: an in situ hybridization study. *J. Comp. Neurol.* 300 287–300. 10.1002/cne.903000302 2266188

[B9] Anastasiades P. G. Collins D. P. Carter A. G. (2021). Mediodorsal and ventromedial thalamus engage distinct L1 circuits in the prefrontal cortex. *Neuron* 109 314.e4–330.e4. 10.1016/j.neuron.2020.10.031 33188733 PMC7855187

[B10] Aoki C. Pickel V. M. (1989). Neuropeptide Y in the cerebral cortex and the caudate-putamen nuclei: ultrastructural basis for interactions with GABAergic and non-GABAergic neurons. *J. Neurosci.* 9 4333–4354. 10.1523/JNEUROSCI.09-12-04333.1989 2687439 PMC6569625

[B11] Appleyard S. M. Patterson T. A. Jin W. Chavkin C. (1997). Agonist-induced phosphorylation of the kappa-opioid receptor. *J. Neurochem.* 69 2405–2412. 10.1046/j.1471-4159.1997.69062405.x 9375672

[B12] Arnsten A. F. (2015). Stress weakens prefrontal networks: molecular insults to higher cognition. *Nat. Neurosci.* 18 1376–1385. 10.1038/nn.4087 26404712 PMC4816215

[B13] Augustine F. Rajendran S. Singer H. S. (2020). Cortical endogenous opioids and their role in facilitating repetitive behaviors in deer mice. *Behav. Brain Res.* 379:112317. 10.1016/j.bbr.2019.112317 31676208

[B14] Augusto E. Gambino F. (2019). Can NMDA spikes dictate computations of local networks and behavior? *Front. Mol. Neurosci.* 12:238. 10.3389/fnmol.2019.00238 31611774 PMC6777373

[B15] Bacci A. Huguenard J. R. Prince D. A. (2002). Differential modulation of synaptic transmission by neuropeptide Y in rat neocortical neurons. *Proc. Natl. Acad. Sci.* 99 17125–17130. 10.1073/pnas.012481899 12482942 PMC139280

[B16] Baldwin G. S. Patel O. Shulkes A. (2010). Evolution of gastrointestinal hormones: the cholecystokinin/gastrin family. *Curr. Opin. Endocrinol. Diabetes Obes.* 17 77–88. 10.1097/MED.0b013e328334e535 19952740

[B17] Ballaz S. J. Bourin M. Akil H. Watson S. J. (2020). Blockade of the cholecystokinin CCK-2 receptor prevents the normalization of anxiety levels in the rat. *Prog. Neuropsychopharmacol. Biol. Psychiatry* 96:109761. 10.1016/j.pnpbp.2019.109761 31526831 PMC6935156

[B18] Bals-Kubik R. Ableitner A. Herz A. Shippenberg T. S. (1993). Neuroanatomical sites mediating the motivational effects of opioids as mapped by the conditioned place preference paradigm in rats. *J. Pharmacol. Exp. Ther.* 264 489–495. 8093731

[B19] Baraban S. C. Tallent M. K. (2004). Interneuron diversity series: interneuronal neuropeptides–endogenous regulators of neuronal excitability. *Trends Neurosci.* 27 135–142. 10.1016/j.tins.2004.01.008 15036878

[B20] Barnett P. (2003). Somatostatin and somatostatin receptor physiology. *Endocrine* 20 255–264. 10.1385/endo:20:3:255 12721505

[B21] Bazov I. Kononenko O. Watanabe H. Kuntic V. Sarkisyan D. Taqi M. M. (2013). The endogenous opioid system in human alcoholics: molecular adaptations in brain areas involved in cognitive control of addiction. *Addict. Biol.* 18 161–169. 10.1111/j.1369-1600.2011.00366.x 21955155

[B22] Bazov I. Sarkisyan D. Kononenko O. Watanabe H. Taqi M. M. Stalhandske L. (2018a). Neuronal expression of opioid gene is controlled by dual epigenetic and transcriptional mechanism in human brain. *Cereb. Cortex* 28 3129–3142. 10.1093/cercor/bhx181 28968778 PMC6887740

[B23] Bazov I. Sarkisyan D. Kononenko O. Watanabe H. Yakovleva T. Hansson A. C. (2018b). Dynorphin and kappa-opioid receptor dysregulation in the dopaminergic reward system of human alcoholics. *Mol. Neurobiol.* 55 7049–7061. 10.1007/s12035-017-0844-4 29383684 PMC6061161

[B24] Bean A. J. Zhang X. Hokfelt T. (1994). Peptide secretion: what do we know? *FASEB J.* 8 630–638. 10.1096/fasebj.8.9.8005390 8005390

[B25] Beinfeld M. C. Meyer D. K. Eskay R. L. Jensen R. T. Brownstein M. J. (1981). The distribution of cholecystokinin immunoreactivity in the central nervous system of the rat as determined by radioimmunoassay. *Brain Res.* 212 51–57. 10.1016/0006-8993(81)90031-7 7225864

[B26] Binneman B. Feltner D. Kolluri S. Shi Y. Qiu R. Stiger T. (2008). A 6-week randomized, placebo-controlled trial of CP-316,311 (a selective CRH1 antagonist) in the treatment of major depression. *Am. J. Psychiatry* 165 617–620. 10.1176/appi.ajp.2008.07071199 18413705

[B27] Birdsong W. T. Jongbloets B. C. Engeln K. A. Wang D. Scherrer G. Mao T. (2019). Synapse-specific opioid modulation of thalamo-cortico-striatal circuits. *eLife* 8:e45146. 10.7554/eLife.45146 31099753 PMC6541437

[B28] Bison S. Crews F. (2003). Alcohol withdrawal increases neuropeptide Y immunoreactivity in rat brain. *Alcohol. Clin. Exp. Res.* 27 1173–1183. 10.1097/01.ALC.0000075827.74538.FE 12878925

[B29] Bittencourt J. C. Vaughan J. Arias C. Rissman R. A. Vale W. W. Sawchenko P. E. (1999). Urocortin expression in rat brain: evidence against a pervasive relationship of urocortin-containing projections with targets bearing type 2 CRF receptors. *J. Comp. Neurol.* 415 285–312. 10.1002/(sici)1096-9861(19991220)415:3<285::aid-cne1>3.0.co;2-0 10553117

[B30] Bologna E. Leroux P. (2000). Identification of multiple somatostatin receptors in the rat somatosensory cortex during development. *J. Comp. Neurol.* 420 466–480. 10.1002/(sici)1096-9861(20000515)420:4<466::aid-cne5>3.0.co;2-w 10805921

[B31] Bonanno G. Parodi B. Cafaggi S. Raiteri M. (1991). Somatostatin release from rat cerebral cortex synaptosomes. *J. Neurochem.* 57 1258–1264. 10.1111/j.1471-4159.1991.tb08287.x 1680160

[B32] Boutros N. Der-Avakian A. Semenova S. Lee S. Markou A. (2016). Risky choice and brain CRF after adolescent ethanol vapor exposure and social stress in adulthood. *Behav. Brain Res.* 311 160–166. 10.1016/j.bbr.2016.05.038 27217101 PMC5253236

[B33] Brar B. K. Chen A. Perrin M. H. Vale W. (2004). Specificity and regulation of extracellularly regulated kinase1/2 phosphorylation through corticotropin-releasing factor (CRF) receptors 1 and 2beta by the CRF/urocortin family of peptides. *Endocrinology* 145 1718–1729. 10.1210/en.2003-1023 14670995

[B34] Brazeau P. Vale W. Burgus R. Ling N. Butcher M. Rivier J. (1973). Hypothalamic polypeptide that inhibits the secretion of immunoreactive pituitary growth hormone. *Science* 179 77–79. 10.1126/science.179.4068.77 4682131

[B35] Brockway D. F. Crowley N. A. (2020). Turning the ’tides on neuropsychiatric diseases: the role of peptides in the prefrontal cortex. *Front. Behav. Neurosci.* 14:588400. 10.3389/fnbeh.2020.588400 33192369 PMC7606924

[B36] Broom D. C. Jutkiewicz E. M. Rice K. C. Traynor J. R. Woods J. H. (2002). Behavioral effects of delta-opioid receptor agonists: potential antidepressants? *Jpn. J. Pharmacol.* 90 1–6. 10.1254/jjp.90.1 12396021

[B37] Brown J. A. Horvath S. Garbett K. A. Schmidt M. J. Everheart M. Gellert L. (2014). The role of cannabinoid 1 receptor expressing interneurons in behavior. *Neurobiol. Dis.* 63 210–221. 10.1016/j.nbd.2013.11.001 24239560 PMC3946968

[B38] Brown M. R. Rivier C. Vale W. (1984). Central nervous system regulation of adrenocorticotropin secretion: role of somatostatins. *Endocrinology* 114 1546–1549. 10.1210/endo-114-5-1546 6143656

[B39] Browne C. A. Jacobson M. L. Lucki I. (2020). Novel targets to treat depression: opioid-based therapeutics. *Harv. Rev. Psychiatry* 28 40–59. 10.1097/HRP.0000000000000242 31913981

[B40] Bruchas M. R. Chavkin C. (2010). Kinase cascades and ligand-directed signaling at the kappa opioid receptor. *Psychopharmacology* 210 137–147. 10.1007/s00213-010-1806-y 20401607 PMC3671863

[B41] Burbach J. P. (2011). What are neuropeptides? *Methods Mol. Biol.* 789 1–36. 10.1007/978-1-61779-310-3_1 21922398

[B42] Butelman E. R. Yuferov V. Kreek M. J. (2012). kappa-opioid receptor/dynorphin system: genetic and pharmacotherapeutic implications for addiction. *Trends Neurosci.* 35 587–596. 10.1016/j.tins.2012.05.005 22709632 PMC3685470

[B43] Cahill E. Pascoli V. Trifilieff P. Savoldi D. Kappes V. Luscher C. (2014). D1R/GluN1 complexes in the striatum integrate dopamine and glutamate signalling to control synaptic plasticity and cocaine-induced responses. *Mol. Psychiatry* 19 1295–1304. 10.1038/mp.2014.73 25070539 PMC4255088

[B44] Carlen M. (2017). What constitutes the prefrontal cortex? *Science* 358 478–482. 10.1126/science.aan8868 29074767

[B45] Castillo P. E. Salin P. A. Weisskopf M. G. Nicoll R. A. (1996). Characterizing the site and mode of action of dynorphin at hippocampal mossy fiber synapses in the guinea pig. *J. Neurosci.* 16 5942–5950. 10.1523/JNEUROSCI.16-19-05942.1996 8815876 PMC6579175

[B46] Castro D. C. Bruchas M. R. (2019). A motivational and neuropeptidergic hub: anatomical and functional diversity within the nucleus accumbens shell. *Neuron* 102 529–552. 10.1016/j.neuron.2019.03.003 31071288 PMC6528838

[B47] Caudle R. M. Dubner R. (1998). Ifenprodil blocks the excitatory effects of the opioid peptide dynorphin 1-17 on NMDA receptor-mediated currents in the CA3 region of the guinea pig hippocampus. *Neuropeptides* 32 87–95. 10.1016/s0143-4179(98)90022-1 9571650

[B48] Chalifoux J. R. Carter A. G. (2010). GABAB receptors modulate NMDA receptor calcium signals in dendritic spines. *Neuron* 66 101–113. 10.1016/j.neuron.2010.03.012 20399732 PMC2861500

[B49] Chalmers D. T. Lovenberg T. W. Grigoriadis D. E. Behan D. P. De Souza E. B. (1996). Corticotrophin-releasing factor receptors: from molecular biology to drug design. *Trends Pharmacol. Sci.* 17 166–172. 10.1016/0165-6147(96)81594-x 8984745

[B50] Chan R. K. Vale W. W. Sawchenko P. E. (2000). Paradoxical activational effects of a corticotropin-releasing factor-binding protein “ligand inhibitor” in rat brain. *Neuroscience* 101 115–129. 10.1016/s0306-4522(00)00322-5 11068141

[B51] Chan-Palay V. Allen Y. Lang W. Haesler U. Polak J. (1985). I. Cytology and distribution in normal human cerebral cortex of neurons immunoreactive with antisera against neuropeptide Y. *J. Comp. Neurol.* 238 382–389. 10.1002/cne.902380403 2413087

[B52] Chavkin C. (2000). Dynorphins are endogenous opioid peptides released from granule cells to act neurohumorly and inhibit excitatory neurotransmission in the hippocampus. *Prog. Brain Res.* 125 363–367. 10.1016/S0079-6123(00)25025-5 11098672

[B53] Chavkin C. Bakhit C. Weber E. Bloom F. E. (1983). Relative contents and concomitant release of prodynorphin/neoendorphin-derived peptides in rat hippocampus. *Proc. Natl. Acad. Sci. U.S.A.* 80 7669–7673. 10.1073/pnas.80.24.7669 6143317 PMC534402

[B54] Chavkin C. Goldstein A. (1981). Specific receptor for the opioid peptide dynorphin: structure–activity relationships. *Proc. Natl. Acad. Sci. U.S.A.* 78 6543–6547. 10.1073/pnas.78.10.6543 6118865 PMC349077

[B55] Chavkin C. James I. F. Goldstein A. (1982). Dynorphin is a specific endogenous ligand of the kappa opioid receptor. *Science* 215 413–415. 10.1126/science.6120570 6120570

[B56] Che T. English J. Krumm B. E. Kim K. Pardon E. Olsen R. H. J. (2020). Nanobody-enabled monitoring of kappa opioid receptor states. *Nat. Commun.* 11:1145. 10.1038/s41467-020-14889-7 32123179 PMC7052193

[B57] Chen C. Willhouse A. H. Huang P. Ko N. Wang Y. Xu B. (2020). Characterization of a knock-in mouse line expressing a fusion protein of kappa opioid receptor conjugated with tdTomato: 3-dimensional brain imaging via CLARITY. *eNeuro* 7:ENEURO.0028-20.2020. 10.1523/ENEURO.0028-20.2020 32561573 PMC7385665

[B58] Chen G. Van Den Pol A. N. (1996). Multiple NPY receptors coexist in pre-and postsynaptic sites: inhibition of GABA release in isolated self-innervating SCN neurons. *J. Neurosci.* 16 7711–7724. 10.1523/JNEUROSCI.16-23-07711.1996 8922427 PMC6579101

[B59] Chen L. Gu Y. Huang L. Y. (1995). The mechanism of action for the block of NMDA receptor channels by the opioid peptide dynorphin. *J. Neurosci.* 15 4602–4611. 10.1523/JNEUROSCI.15-06-04602.1995 7540680 PMC6577699

[B60] Chen P. Lou S. Huang Z. H. Wang Z. Shan Q. H. Wang Y. (2020). Prefrontal cortex corticotropin-releasing factor neurons control behavioral style selection under challenging situations. *Neuron* 106 301.e7–315.e7. 10.1016/j.neuron.2020.01.033 32101698

[B61] Chini B. Verhage M. Grinevich V. (2017). The action radius of oxytocin release in the mammalian CNS: from single vesicles to behavior. *Trends Pharmacol. Sci.* 38 982–991. 10.1016/j.tips.2017.08.005 28899620

[B62] Christie M. P. Simerska P. Jen F. E. Hussein W. M. Rawi M. F. Hartley-Tassell L. E. (2014). A drug delivery strategy: binding enkephalin to asialoglycoprotein receptor by enzymatic galactosylation. *PLoS One* 9:e95024. 10.1371/journal.pone.0095024 24736570 PMC3988166

[B63] Coffield J. A. Miletic V. (1987). Immunoreactive enkephalin is contained within some trigeminal and spinal neurons projecting to the rat medial thalamus. *Brain Res.* 425 380–383. 10.1016/0006-8993(87)90525-7 2827847

[B64] Cohen H. Liu T. Kozlovsky N. Kaplan Z. Zohar J. Mathé A. A. (2012). The neuropeptide Y (NPY)-ergic system is associated with behavioral resilience to stress exposure in an animal model of post-traumatic stress disorder. *Neuropsychopharmacology* 37 350–363. 10.1038/npp.2011.230 21976046 PMC3242318

[B65] Comeras L. B. Hormer N. Mohan Bethuraj P. Tasan R. O. (2021). NPY released from GABA neurons of the dentate gyrus specially reduces contextual fear without affecting cued or trace fear. *Front. Synap. Neurosci.* 13:635726. 10.3389/fnsyn.2021.635726 34122036 PMC8187774

[B66] Cortright D. N. Nicoletti A. Seasholtz A. F. (1995). Molecular and biochemical characterization of the mouse brain corticotropin-releasing hormone-binding protein. *Mol. Cell Endocrinol.* 111 147–157. 10.1016/0303-7207(95)03558-o 7556876

[B67] Crowley N. A. Kash T. L. (2015). Kappa opioid receptor signaling in the brain: circuitry and implications for treatment. *Prog. Neuropsychopharmacol. Biol. Psychiatry* 62 51–60. 10.1016/j.pnpbp.2015.01.001 25592680 PMC4465498

[B68] Dao N. C. Brockway D. F. Crowley N. A. (2019). In vitro optogenetic characterization of neuropeptide release from prefrontal cortical somatostatin neurons. *Neuroscience* 419 1–4. 10.1016/j.neuroscience.2019.08.014 31487544

[B69] Dautzenberg F. M. Hauger R. L. (2002). The CRF peptide family and their receptors: yet more partners discovered. *Trends Pharmacol. Sci.* 23 71–77. 10.1016/s0165-6147(02)01946-6 11830263

[B70] Davies P. Katzman R. Terry R. D. (1980). Reduced somatostatin-like immunoreactivity in cerebral cortex from cases of Alzheimer disease and Alzheimer senile dementa. *Nature* 288 279–280. 10.1038/288279a0 6107862

[B71] Dawbarn D. Hunt S. Emson P. (1984). Neuropeptide Y: regional distribution chromatographic characterization and immunohistochemical demonstration in post-mortem human brain. *Brain Res.* 296 168–173. 10.1016/0006-8993(84)90526-2 6201235

[B72] Day R. Lazure C. Basak A. Boudreault A. Limperis P. Dong W. (1998). Prodynorphin processing by proprotein convertase 2. Cleavage at single basic residues and enhanced processing in the presence of carboxypeptidase activity. *J. Biol. Chem.* 273 829–836. 10.1074/jbc.273.2.829 9422738

[B73] de Lecea L. (2008). Cortistatin–functions in the central nervous system. *Mol. Cell Endocrinol.* 286 88–95. 10.1016/j.mce.2007.12.014 18374474

[B74] Decressac M. Barker R. A. (2012). Neuropeptide Y and its role in CNS disease and repair. *Exp. Neurol.* 238 265–272. 10.1016/j.expneurol.2012.09.004 23022456

[B75] Dedic N. Chen A. Deussing J. M. (2018). The CRF family of neuropeptides and their receptors - mediators of the central stress response. *Curr. Mol. Pharmacol.* 11 4–31. 10.2174/1874467210666170302104053 28260504 PMC5930453

[B76] Delfs J. R. Dichter M. A. (1983). Effects of somatostatin on mammalian cortical neurons in culture: physiological actions and unusual dose response characteristics. *J. Neurosci.* 3 1176–1188. 10.1523/JNEUROSCI.03-06-01176.1983 6133919 PMC6564616

[B77] DePaoli A. M. Hurley K. M. Yasada K. Reisine T. Bell G. (1994). Distribution of kappa opioid receptor mRNA in adult mouse brain: an in situ hybridization histochemistry study. *Mol. Cell Neurosci.* 5 327–335. 10.1006/mcne.1994.1039 7804602

[B78] Deussing J. M. Chen A. (2018). The corticotropin-releasing factor family: physiology of the stress response. *Physiol. Rev.* 98 2225–2286. 10.1152/physrev.00042.2017 30109816

[B79] Devi L. A. (2001). Heterodimerization of G-protein-coupled receptors: pharmacology, signaling and trafficking. *Trends Pharmacol. Sci.* 22 532–537. 10.1016/s0165-6147(00)01799-5 11583811

[B80] Dhawan B. N. Cesselin F. Raghubir R. Reisine T. Bradley P. B. Portoghese P. S. (1996). International Union of Pharmacology. XII. Classification of opioid receptors. *Pharmacol. Rev.* 48 567–592. 8981566

[B81] DiFeliceantonio A. G. Mabrouk O. S. Kennedy R. T. Berridge K. C. (2012). Enkephalin surges in dorsal neostriatum as a signal to eat. *Curr. Biol.* 22 1918–1924. 10.1016/j.cub.2012.08.014 23000149 PMC3482294

[B82] Dong A. He K. Dudok B. Farrell J. S. Guan W. Liput D. J. (2021). A fluorescent sensor for spatiotemporally resolved imaging of endocannabinoid dynamics in vivo. *Nat. Biotechnol.* [Epub ahead of print]. 10.1038/s41587-021-01074-4 34764491 PMC9091059

[B83] Dournaud P. Gu Y. Z. Schonbrunn A. Mazella J. Tannenbaum G. S. Beaudet A. (1996). Localization of the somatostatin receptor SST2A in rat brain using a specific anti-peptide antibody. *J. Neurosci.* 16 4468–4478. 10.1523/JNEUROSCI.16-14-04468.1996 8699257 PMC6578860

[B84] Drake C. T. Terman G. W. Simmons M. L. Milner T. A. Kunkel D. D. Schwartzkroin P. A. (1994). Dynorphin opioids present in dentate granule cells may function as retrograde inhibitory neurotransmitters. *J. Neurosci.* 14 3736–3750. 10.1523/JNEUROSCI.14-06-03736.1994 7911518 PMC6576943

[B85] D’Souza R. D. Burkhalter A. (2017). A laminar organization for selective cortico-cortical communication. *Front. Neuroanat.* 11:71. 10.3389/fnana.2017.00071 28878631 PMC5572236

[B86] Duffet L. Kosar S. Panniello M. Viberti B. Bracey E. Zych A. D. (2022). A genetically encoded sensor for in vivo imaging of orexin neuropeptides. *Nat. Methods* 19 231–241. 10.1038/s41592-021-01390-2 35145320 PMC8831244

[B87] Dumont Y. Martel J.-C. Fournier A. St-Pierre S. Quirion R. (1992). Neuropeptide Y and neuropeptide Y receptor subtypes in brain and peripheral tissues. *Prog. Neurobiol.* 38 125–167. 10.1016/0301-0082(92)90038-g 1312243

[B88] Dunlop B. W. Rothbaum B. O. Binder E. B. Duncan E. Harvey P. D. Jovanovic T. (2014). Evaluation of a corticotropin releasing hormone type 1 receptor antagonist in women with posttraumatic stress disorder: study protocol for a randomized controlled trial. *Trials* 15:240. 10.1186/1745-6215-15-240 24950747 PMC4082482

[B89] Duque-Diaz E. Diaz-Cabiale Z. Narvaez J. A. Covenas R. (2017). Mapping of enkephalins and adrenocorticotropic hormone in the squirrel monkey brainstem. *Anat. Sci. Int.* 92 275–292. 10.1007/s12565-016-0333-2 26897373

[B90] Dvorakova M. C. Kruzliak P. Rabkin S. W. (2014). Role of neuropeptides in cardiomyopathies. *Peptides* 61 1–6. 10.1016/j.peptides.2014.08.004 25149360

[B91] Eberly L. B. Dudley C. A. Moss R. L. (1983). Iontophoretic mapping of corticotropin-releasing factor (CRF) sensitive neurons in the rat forebrain. *Peptides* 4 837–841. 10.1016/0196-9781(83)90077-3 6369268

[B92] Ehlers C. L. Li T. K. Lurneng L. Hwang B. H. Somes C. Jimenez P. (1998). Neuropeptide Y levels in ethanol-naive alcohol-preferring and nonpreferring rats and in Wistar rats after ethanol exposure. *Alcohol. Clin. Exp. Res.* 22 1778–1782. 10.1111/j.1530-0277.1998.tb03979.x 9835294

[B93] Ehrlich A. T. Semache M. Gross F. Da Fonte D. F. Runtz L. Colley C. (2019). Biased signaling of the Mu opioid receptor revealed in native neurons. *iScience* 14 47–57. 10.1016/j.isci.2019.03.011 30925410 PMC6439305

[B94] Eiden L. E. Goosens K. A. Jacobson K. A. Leggio L. Zhang L. (2020). Peptide-liganded G protein-coupled receptors as neurotherapeutics. *ACS Pharmacol. Transl. Sci.* 3 190–202. 10.1021/acsptsci.0c00017 32296762 PMC7155190

[B95] Engin E. Stellbrink J. Treit D. Dickson C. T. (2008). Anxiolytic and antidepressant effects of intracerebroventricularly administered somatostatin: behavioral and neurophysiological evidence. *Neuroscience* 157 666–676. 10.1016/j.neuroscience.2008.09.037 18940236

[B96] Engin E. Treit D. (2009). Anxiolytic and antidepressant actions of somatostatin: the role of sst2 and sst3 receptors. *Psychopharmacology* 206 281–289. 10.1007/s00213-009-1605-5 19609508

[B97] Epelbaum J. (1986). Somatostatin in the central nervous system: physiology and pathological modifications. *Prog. Neurobiol.* 27 63–100. 10.1016/0301-0082(86)90012-2 2874591

[B98] Esch F. Bohlen P. Ling N. Benoit R. Brazeau P. Guillemin R. (1980). Primary structure of ovine hypothalamic somatostatin-28 and somatostatin-25. *Proc. Natl. Acad. Sci. U.S.A.* 77 6827–6831. 10.1073/pnas.77.11.6827 6109284 PMC350383

[B99] Faouzi A. Varga B. R. Majumdar S. (2020). Biased opioid ligands. *Molecules* 25:4257. 10.3390/molecules25184257 32948048 PMC7570672

[B100] Farrell S. R. Rankin D. R. Brecha N. C. Barnes S. (2014). Somatostatin receptor subtype 4 modulates L-type calcium channels via Gbetagamma and PKC signaling in rat retinal ganglion cells. *Channels* 8 519–527. 10.4161/19336950.2014.967623 25483286 PMC4594562

[B101] Fassini A. Scopinho A. A. Resstel L. B. Correa F. M. (2014). Opioid receptors in the prelimbic cortex modulate restraint stress-induced cardiovascular responses in the rat. *Neuropharmacology* 85 367–374. 10.1016/j.neuropharm.2014.04.019 24813527

[B102] Fassini A. Scopinho A. A. Resstel L. B. Correa F. M. (2015). kappa-opioid receptors in the infralimbic cortex modulate the cardiovascular responses to acute stress. *Exp. Physiol.* 100 377–387. 10.1113/expphysiol.2014.084020 25641629

[B103] Fenno L. E. Mattis J. Ramakrishnan C. Hyun M. Lee S. Y. He M. (2014). Targeting cells with single vectors using multiple-feature Boolean logic. *Nat. Methods* 11 763–772. 10.1038/nmeth.2996 24908100 PMC4085277

[B104] Fenno L. E. Ramakrishnan C. Kim Y. S. Evans K. E. Lo M. Vesuna S. (2020). Comprehensive dual- and triple-feature intersectional single-vector delivery of diverse functional payloads to cells of behaving mammals. *Neuron* 107 836.e11–853.e11. 10.1016/j.neuron.2020.06.003 32574559 PMC7687746

[B105] Ferguson B. R. Gao W. J. (2018). PV interneurons: critical regulators of E/I balance for prefrontal cortex-dependent behavior and psychiatric disorders. *Front. Neural Circ.* 12:37. 10.3389/fncir.2018.00037 29867371 PMC5964203

[B106] Feuerstein G. Faden A. I. (1984). Cardiovascular effects of dynorphin A-(1-8), dynorphin A-(1-13) and dynorphin A-(1-17) microinjected into the preoptic medialis nucleus of the rat. *Neuropeptides* 5 295–298. 10.1016/0143-4179(84)90086-6 6152328

[B107] Fischli W. Goldstein A. Hunkapiller M. W. Hood L. E. (1982). Isolation and amino acid sequence analysis of a 4,000-dalton dynorphin from porcine pituitary. *Proc. Natl. Acad. Sci. U.S.A.* 79 5435–5437. 10.1073/pnas.79.17.5435 6127674 PMC346912

[B108] Fishell G. Kepecs A. (2020). Interneuron types as attractors and controllers. *Annu. Rev. Neurosci.* 43 1–30. 10.1146/annurev-neuro-070918-050421 31299170 PMC7064158

[B109] Fishell G. Rudy B. (2011). Mechanisms of inhibition within the telencephalon: “where the wild things are”. *Annu. Rev. Neurosci.* 34 535–567. 10.1146/annurev-neuro-061010-113717 21469958 PMC3556485

[B110] Floresco S. (2013). Prefrontal dopamine and behavioral flexibility: shifting from an “inverted-U” toward a family of functions. *Front. Neurosci.* 7:62. 10.3389/fnins.2013.00062 23626521 PMC3630325

[B111] Fox K. (2018). Deconstructing the cortical column in the barrel cortex. *Neuroscience* 368 17–28. 10.1016/j.neuroscience.2017.07.034 28739527

[B112] Fricker L. D. Margolis E. B. Gomes I. Devi L. A. (2020). Five decades of research on opioid peptides: current knowledge and unanswered questions. *Mol. Pharmacol.* 98 96–108. 10.1124/mol.120.119388 32487735 PMC7330675

[B113] Fung S. J. Fillman S. G. Webster M. J. Shannon Weickert C. (2014). Schizophrenia and bipolar disorder show both common and distinct changes in cortical interneuron markers. *Schizophr. Res.* 155 26–30. 10.1016/j.schres.2014.02.021 24674775

[B114] Gallopin T. Geoffroy H. Rossier J. Lambolez B. (2006). Cortical sources of CRF, NKB, and CCK and their effects on pyramidal cells in the neocortex. *Cereb. Cortex* 16 1440–1452. 10.1093/cercor/bhj081 16339088

[B115] Gangarossa G. Castell L. Castro L. Tarot P. Veyrunes F. Vincent P. (2019). Contrasting patterns of ERK activation in the tail of the striatum in response to aversive and rewarding signals. *J. Neurochem.* 151 204–226. 10.1111/jnc.14804 31245856

[B116] Gehlert D. R. (1998). Multiple receptors for the pancreatic polypeptide (PP-fold) family: physiological implications. *Proc. Soc. Exp. Biol. Med.* 218 7–22. 10.3181/00379727-218-44263 9572148

[B117] Geracioti T. D. Jr. Strawn J. R. Ekhator N. N. Wortman M. Kasckow J. (2009). “Neuroregulatory peptides of central nervous system origin: from laboratory to clinic,” in *Hormones, Brain and Behavior*, 2nd Edn, eds Donald A. P. A. Pfaff W. Etgen A. M. Fahrbach S. E. Rubin R. T. (San Diego, CA: Academic Press), 2541–2596. 10.1016/b978-008088783-8.00082-6

[B118] Giacomini J. L. Geiduschek E. Selleck R. A. Sadeghian K. Baldo B. A. (2021). Dissociable control of mu-opioid-driven hyperphagia vs. food impulsivity across subregions of medial prefrontal, orbitofrontal, and insular cortex. *Neuropsychopharmacology* 46 1981–1989. 10.1038/s41386-021-01068-5 34226656 PMC8429588

[B119] Giraud P. Castanas E. Patey G. Oliver C. Rossier J. (1983). Regional distribution of methionine-enkephalin-Arg6-Phe7 in the rat brain: comparative study with the distribution of other opioid peptides. *J. Neurochem.* 41 154–160. 10.1111/j.1471-4159.1983.tb11827.x 6688089

[B120] Goldman-Rakic P. S. Muly E. C. III Williams G. V. (2000). D1 receptors in prefrontal cells and circuits. *Brain Res. Rev.* 31 295–301. 10.1016/s0165-0173(99)00045-4 10719156

[B121] Goldstein R. Z. Volkow N. D. (2011). Dysfunction of the prefrontal cortex in addiction: neuroimaging findings and clinical implications. *Nat. Rev. Neurosci.* 12 652–669. 10.1038/nrn3119 22011681 PMC3462342

[B122] Gomes-Porras M. Cardenas-Salas J. Alvarez-Escola C. (2020). Somatostatin analogs in clinical practice: a review. *Int. J. Mol. Sci.* 21:1682. 10.3390/ijms21051682 32121432 PMC7084228

[B123] Gozes I. Brenneman D. E. Geppetti P. Kastin A. J. Mains R. E. Moody T. W. (2001). Neuropeptides: brain messengers of many faces. *Trends Neurosci.* 24 687–690. 10.1016/s0166-2236(00)02001-4 11718856

[B124] Grammatopoulos D. K. Chrousos G. P. (2002). Functional characteristics of CRH receptors and potential clinical applications of CRH-receptor antagonists. *Trends Endocrinol. Metab.* 13 436–444. 10.1016/s1043-2760(02)00670-7 12431840

[B125] Granger A. J. Mulder N. Saunders A. Sabatini B. L. (2016). Cotransmission of acetylcholine and GABA. *Neuropharmacology* 100 40–46. 10.1016/j.neuropharm.2015.07.031 26220313 PMC4584188

[B126] Granger A. J. Wang W. Robertson K. El-Rifai M. Zanello A. Bistrong K. (2020). Cortical ChAT+ neurons co-transmit acetylcholine and GABA in a target- and brain-region 2 specific manner. *eLife* 9:e57749. 10.1101/2020.04.20.051276 PMC7360370 32613945

[B127] Grim T. W. Acevedo-Canabal A. Bohn L. M. (2020). Toward directing opioid receptor signaling to refine opioid therapeutics. *Biol. Psychiatry* 87 15–21. 10.1016/j.biopsych.2019.10.020 31806082 PMC6919561

[B128] Guo Q. Wang L. Yuan W. Li L. Zhang J. Hou W. (2020). Different effects of chronic social defeat on social behavior and the brain CRF system in adult male C57 mice with different susceptibilities. *Behav. Brain Res.* 384:112553. 10.1016/j.bbr.2020.112553 32057826

[B129] Hallberg M. (2015). Neuropeptides: metabolism to bioactive fragments and the pharmacology of their receptors. *Med. Res. Rev.* 35 464–519. 10.1002/med.21323 24894913

[B130] Hare B. D. Duman R. S. (2020). Prefrontal cortex circuits in depression and anxiety: contribution of discrete neuronal populations and target regions. *Mol. Psychiatry* 25 2742–2758. 10.1038/s41380-020-0685-9 32086434 PMC7442605

[B131] Harris K. D. Shepherd G. M. (2015). The neocortical circuit: themes and variations. *Nat. Neurosci.* 18 170–181. 10.1038/nn.3917 25622573 PMC4889215

[B132] Heilig M. (2004). The NPY system in stress, anxiety and depression. *Neuropeptides* 38 213–224. 10.1016/j.npep.2004.05.002 15337373

[B133] Heilig M. Söderpalm B. Engel J. A. Widerlöv E. (1989). Centrally administered neuropeptide Y (NPY) produces anxiolytic-like effects in animal anxiety models. *Psychopharmacology* 98 524–529. 10.1007/BF00441953 2570434

[B134] Heldin C. H. Lu B. Evans R. Gutkind J. S. (2016). Signals and receptors. *Cold Spring Harb. Perspect. Biol.* 8:a005900. 10.1101/cshperspect.a005900 27037414 PMC4817805

[B135] Hendry S. Jones E. DeFelipe J. Schmechel D. Brandon C. Emson P. (1984). Neuropeptide-containing neurons of the cerebral cortex are also GABAergic. *Proc. Natl. Acad. Sci. U.S.A.* 81 6526–6530. 10.1073/pnas.81.20.6526 6149547 PMC391957

[B136] Hendry S. H. Jones E. G. Beinfeld M. C. (1983). Cholecystokinin immunoreactive neurons in rat and monkey cerebral cortex make symmetric synapses and have intimate associations with blood vessels. *Proc. Natl. Acad. Sci. U.S.A.* 80 2400–2404. 10.1073/pnas.80.8.2400 6132387 PMC393828

[B137] Herbert J. (1993). Peptides in the limbic system: neurochemical codes for co-ordinated adaptive responses to behavioural and physiological demand. *Prog. Neurobiol.* 41 723–791. 10.1016/0301-0082(93)90033-o 7908139

[B138] Herkenham M. (1987). Mismatches between neurotransmitter and receptor localizations in brain: observations and implications. *Neuroscience* 23 1–38. 10.1016/0306-4522(87)90268-5 2891080

[B139] Hillhouse E. W. Grammatopoulos D. K. (2006). The molecular mechanisms underlying the regulation of the biological activity of corticotropin-releasing hormone receptors: implications for physiology and pathophysiology. *Endocr. Rev.* 27 260–286. 10.1210/er.2005-0034 16484629

[B140] Hirsch D. Zukowska Z. (2012). NPY and stress 30 years later: the peripheral view. *Cell. Mol. Neurobiol.* 32 645–659. 10.1007/s10571-011-9793-z 22271177 PMC3492947

[B141] Hoftman G. D. Volk D. W. Bazmi H. H. Li S. Sampson A. R. Lewis D. A. (2015). Altered cortical expression of GABA-related genes in schizophrenia: illness progression vs developmental disturbance. *Schizophr. Bull.* 41 180–191. 10.1093/schbul/sbt178 24361861 PMC4266281

[B142] Hokfelt T. Broberger C. Xu Z. Q. Sergeyev V. Ubink R. Diez M. (2000). Neuropeptides–an overview. *Neuropharmacology* 39 1337–1356. 10.1016/s0028-3908(00)00010-1 10818251

[B143] Holaday J. W. (1983). Cardiovascular effects of endogenous opiate systems. *Annu. Rev. Pharmacol. Toxicol.* 23 541–594. 10.1146/annurev.pa.23.040183.002545 6307129

[B144] Hook V. Funkelstein L. Lu D. Bark S. Wegrzyn J. Hwang S. R. (2008). Proteases for processing proneuropeptides into peptide neurotransmitters and hormones. *Annu. Rev. Pharmacol. Toxicol.* 48 393–423. 10.1146/annurev.pharmtox.48.113006.094812 18184105 PMC2731677

[B145] Hook V. Lietz C. B. Podvin S. Cajka T. Fiehn O. (2018). Diversity of neuropeptide cell-cell signaling molecules generated by proteolytic processing revealed by neuropeptidomics mass spectrometry. *J. Am. Soc. Mass. Spectrom.* 29 807–816. 10.1007/s13361-018-1914-1 29667161 PMC5946320

[B146] Hoyer D. Bartfai T. (2012). Neuropeptides and neuropeptide receptors: drug targets, and peptide and non-peptide ligands: a tribute to prof. dieter seebach. *Chem. Biodivers.* 9 2367–2387. 10.1002/cbdv.201200288 23161624

[B147] Hughes J. Smith T. W. Kosterlitz H. W. Fothergill L. A. Morgan B. A. Morris H. R. (1975). Identification of two related pentapeptides from the brain with potent opiate agonist activity. *Nature* 258 577–580. 10.1038/258577a0 1207728

[B148] Hupalo S. Berridge C. W. (2016). Working memory impairing actions of corticotropin-releasing factor (CRF) neurotransmission in the prefrontal cortex. *Neuropsychopharmacology* 41 2733–2740. 10.1038/npp.2016.85 27272767 PMC5026742

[B149] Hupalo S. Martin A. J. Green R. K. Devilbiss D. M. Berridge C. W. (2019). Prefrontal corticotropin-releasing factor (CRF) neurons act locally to modulate frontostriatal cognition and circuit function. *J. Neurosci.* 39 2080–2090. 10.1523/JNEUROSCI.2701-18.2019 30651328 PMC6507090

[B150] Hurd Y. L. (1996). Differential messenger RNA expression of prodynorphin and proenkephalin in the human brain. *Neuroscience* 72 767–783. 10.1016/0306-4522(96)00002-4 9157322

[B151] Husum H. Vasquez P. A. J. Mathé A. A. (2001). Changed concentrations of tachykinins and neuropeptide Y in brain of a rat model of depression: lithium treatment normalizes tachykinins. *Neuropsychopharmacology* 24 183–191. 10.1016/S0893-133X(00)00198-6 11120400

[B152] Hwa L. S. Neira S. Pina M. M. Pati D. Calloway R. Kash T. L. (2019). Predator odor increases avoidance and glutamatergic synaptic transmission in the prelimbic cortex via corticotropin-releasing factor receptor 1 signaling. *Neuropsychopharmacology* 44 766–775. 10.1038/s41386-018-0279-2 30470839 PMC6372588

[B153] Ibrahim L. A. Schuman B. Bandler R. Rudy B. Fishell G. (2020). Mining the jewels of the cortex’s crowning mystery. *Curr. Opin. Neurobiol.* 63 154–161. 10.1016/j.conb.2020.04.005 32480351 PMC8075042

[B154] Igarashi H. Fujimori N. Ito T. Nakamura T. Oono T. Nakamura K. (2011). Vasoactive intestinal peptide (VIP) and VIP receptors-elucidation of structure and function for therapeutic applications. *Int. J. Clin. Med.* 02 500–508. 10.4236/ijcm.2011.24084

[B155] Ingram S. M. Krause R. G. II Baldino F. Jr. Skeen L. C. Lewis M. E. (1989). Neuronal localization of cholecystokinin mRNA in the rat brain by using in situ hybridization histochemistry. *J. Comp. Neurol.* 287 260–272. 10.1002/cne.902870209 2794128

[B156] Iwasawa C. Kuzumaki N. Suda Y. Kagawa R. Oka Y. Hattori N. (2019). Reduced expression of somatostatin in GABAergic interneurons derived from induced pluripotent stem cells of patients with parkin mutations. *Mol. Brain* 12:5. 10.1186/s13041-019-0426-7 30658665 PMC6339354

[B157] Jaferi A. Bhatnagar S. (2007). Corticotropin-releasing hormone receptors in the medial prefrontal cortex regulate hypothalamic-pituitary-adrenal activity and anxiety-related behavior regardless of prior stress experience. *Brain Res.* 1186 212–223. 10.1016/j.brainres.2007.07.100 18001698 PMC2175080

[B158] James I. F. Fischli W. Goldstein A. (1984). Opioid receptor selectivity of dynorphin gene products. *J. Pharmacol. Exp. Ther.* 228 88–93. 6141278

[B159] Jiang S. Z. Sweat S. Dahlke S. P. Loane K. Drossel G. Xu W. (2021). Cocaine-dependent acquisition of locomotor sensitization and conditioned place preference requires d1 dopaminergic signaling through a cyclic AMP, NCS-Rapgef2, ERK, and Egr-1/Zif268 pathway. *J. Neurosci.* 41 711–725. 10.1523/JNEUROSCI.1497-20.2020 33268547 PMC7842751

[B160] Jiang X. Shen S. Cadwell C. R. Berens P. Sinz F. Ecker A. S. (2015). Principles of connectivity among morphologically defined cell types in adult neocortex. *Science* 350:aac9462. 10.1126/science.aac9462 26612957 PMC4809866

[B161] Jimenez-Vasquez P. A. Diaz-Cabiale Z. Caberlotto L. Bellido I. Overstreet D. Fuxe K. (2007). Electroconvulsive stimuli selectively affect behavior and neuropeptide Y (NPY) and NPY Y(1) receptor gene expressions in hippocampus and hypothalamus of Flinders Sensitive Line rat model of depression. *Eur. Neuropsychopharmacol.* 17 298–308. 10.1016/j.euroneuro.2006.06.011 16904299

[B162] Johnsen A. H. (1998). Phylogeny-of-the-cholecystokinin-gastrin. *Front. Neuroendocrinol.* 19:163. 10.1006/frne.1997.0163 9578981

[B163] Johnson S. W. North R. A. (1992). Opioids excite dopamine neurons by hyperpolarization of local interneurons. *J. Neurosci.* 12 483–488. 10.1523/JNEUROSCI.12-02-00483.1992 1346804 PMC6575608

[B164] Karagiannis A. Gallopin T. Dávid C. Battaglia D. Geoffroy H. Rossier J. (2009). Classification of NPY-expressing neocortical interneurons. *J. Neurosci.* 29 3642–3659. 10.1523/JNEUROSCI.0058-09.2009 19295167 PMC2750888

[B165] Kash T. L. Pleil K. E. Marcinkiewcz C. A. Lowery-Gionta E. G. Crowley N. Mazzone C. (2015). Neuropeptide regulation of signaling and behavior in the BNST. *Mol. Cells* 38 1–13. 10.14348/molcells.2015.2261 25475545 PMC4314126

[B166] Kecskés M. Henn-Mike N. Agócs-Laboda Á Szõcs S. Petykó Z. Varga C. (2020). Somatostatin expressing GABAergic interneurons in the medial entorhinal cortex preferentially inhibit layer III-V pyramidal cells. *Commun. Biol.* 3 1–13. 10.1038/s42003-020-01496-x 33303963 PMC7728756

[B167] Kehne J. De Lombaert S. (2002). Non-peptidic CRF1 receptor antagonists for the treatment of anxiety, depression and stress disorders. *Curr. Drug Targets CNS Neurol. Disord.* 1 467–493. 10.2174/1568007023339049 12769601

[B168] Keller A. J. Roth M. M. Scanziani M. (2020). Feedback generates a second receptive field in neurons of the visual cortex. *Nature* 582 545–549. 10.1038/s41586-020-2319-4 32499655 PMC7790439

[B169] Kennedy A. Asahina K. Hoopfer E. Inagaki H. Jung Y. Lee H. (2014). Internal states and behavioral decision-making: toward an integration of emotion and cognition. *Cold Spring Harb. Symp. Quant. Biol.* 79 199–210. 10.1101/sqb.2014.79.024984 25948637

[B170] Kennedy R. T. (2013). Emerging trends in in vivo neurochemical monitoring by microdialysis. *Curr. Opin. Chem. Biol.* 17 860–867. 10.1016/j.cbpa.2013.06.012 23856056 PMC3823805

[B171] Ketchesin K. D. Huang N. S. Seasholtz A. F. (2017). Cell type-specific expression of corticotropin-releasing hormone-binding protein in GABAergic interneurons in the prefrontal cortex. *Front. Neuroanat.* 11:90. 10.3389/fnana.2017.00090 29066956 PMC5641307

[B172] Khachaturian H. Lewis M. E. Haber S. N. Houghten R. A. Akil H. Watson S. J. (1985). Prodynorphin peptide immunocytochemistry in rhesus monkey brain. *Peptides* 6 (Suppl. 2) 155–166. 10.1016/0196-9781(85)90149-4 3909123

[B173] Kim H. Kim M. Im S. K. Fang S. (2018). Mouse Cre-LoxP system: general principles to determine tissue-specific roles of target genes. *Lab. Anim. Res.* 34 147–159. 10.5625/lar.2018.34.4.147 30671100 PMC6333611

[B174] Kim S. M. Su C. Y. Wang J. W. (2017). Neuromodulation of innate behaviors in *Drosophila*. *Annu. Rev. Neurosci.* 40 327–348. 10.1146/annurev-neuro-072116-031558 28441115

[B175] Kim T. Gondre-Lewis M. C. Arnaoutova I. Loh Y. P. (2006). Dense-core secretory granule biogenesis. *Physiology* 21 124–133. 10.1152/physiol.00043.2005 16565478

[B176] Kim Y. S. Anderson M. Park K. Zheng Q. Agarwal A. Gong C. (2016). Coupled activation of primary sensory neurons contributes to chronic pain. *Neuron* 91 1085–1096. 10.1016/j.neuron.2016.07.044 27568517 PMC5017920

[B177] Klapstein G. J. Colmers W. F. (1993). On the sites of presynaptic inhibition by neuropeptide Y in rat hippocampus in vitro. *Hippocampus* 3 103–111. 10.1002/hipo.450030111 8395947

[B178] Koenigs M. Grafman J. (2009). Posttraumatic stress disorder: the role of medial prefrontal cortex and amygdala. *Neuroscientist* 15 540–548. 10.1177/1073858409333072 19359671 PMC2771687

[B179] Koob G. F. (2010). The role of CRF and CRF-related peptides in the dark side of addiction. *Brain Res.* 1314 3–14. 10.1016/j.brainres.2009.11.008 19912996 PMC2819562

[B180] Krienen F. M. Goldman M. Zhang Q. del Rosario R. C. H. Florio M. Machold R. (2020). Innovations present in the primate interneuron repertoire. *Nature* 586 262–269. 10.1038/s41586-020-2781-z 32999462 PMC7957574

[B181] Kropotova E. S. Ivleva I. S. Karpenko M. N. Mosevitsky M. I. (2020). Design of enkephalin modifications protected from brain extracellular peptidases providing long-term analgesia. *Bioorg. Med. Chem.* 28:115184. 10.1016/j.bmc.2019.115184 31740204

[B182] Krulich L. Dhariwal A. P. McCann S. M. (1968). Stimulatory and inhibitory effects of purified hypothalamic extracts on growth hormone release from rat pituitary in vitro. *Endocrinology* 83 783–790. 10.1210/endo-83-4-783 4879544

[B183] Kumar U. (2005). Expression of somatostatin receptor subtypes (SSTR1-5) in Alzheimer’s disease brain: an immunohistochemical analysis. *Neuroscience* 134 525–538. 10.1016/j.neuroscience.2005.04.001 15961235

[B184] Lamberts S. W. van der Lely A. J. de Herder W. W. Hofland L. J. (1996). Octreotide. *N. Engl. J. Med.* 334 246–254. 10.1056/NEJM199601253340408 8532003

[B185] Larhammar D. Salaneck E. (2004). Molecular evolution of NPY receptor subtypes. *Neuropeptides* 38 141–151. 10.1016/j.npep.2004.06.002 15337367

[B186] Lau B. K. Ambrose B. P. Thomas C. S. Qiao M. Borgland S. L. (2020). Mu-opioids suppress GABAergic synaptic transmission onto orbitofrontal cortex pyramidal neurons with subregional selectivity. *J. Neurosci.* 40 5894–5907. 10.1523/JNEUROSCI.2049-19.2020 32601247 PMC7392508

[B187] Le Merre P. Ahrlund-Richter S. Carlen M. (2021). The mouse prefrontal cortex: unity in diversity. *Neuron* 109 1925–1944. 10.1016/j.neuron.2021.03.035 33894133

[B188] Lee A. T. Cunniff M. M. See J. Z. Wilke S. A. Luongo F. J. Ellwood I. T. (2019). VIP interneurons contribute to avoidance behavior by regulating information flow across hippocampal-prefrontal networks. *Neuron* 102 1223.e4–1234.e4. 10.1016/j.neuron.2019.04.001 31053407 PMC6800223

[B189] Lee C. C. Miller R. J. (1998). Is there really an NPY Y3 receptor? *Regul. Peptides* 75 71–78. 10.1016/s0167-0115(98)00054-8 9802395

[B190] Lee P. R. Fields R. D. (2021). Activity-dependent gene expression in neurons. *Neuroscientist* 27 355–366. 10.1177/1073858420943515 32727285 PMC8246373

[B191] Lemos J. C. Roth C. A. Chavkin C. (2011). Signaling events initiated by kappa opioid receptor activation: quantification and immunocolocalization using phospho-selective KOR, p38 MAPK, and K(IR) 3.1 antibodies. *Methods Mol. Biol.* 717 197–219. 10.1007/978-1-61779-024-9_11 21370032 PMC3727639

[B192] Lemos Duarte M. Devi L. A. (2020). Post-translational modifications of opioid receptors. *Trends Neurosci.* 43 417–432. 10.1016/j.tins.2020.03.011 32459993 PMC7323054

[B193] Lepousez G. Mouret A. Loudes C. Epelbaum J. Viollet C. (2010). Somatostatin contributes to in vivo gamma oscillation modulation and odor discrimination in the olfactory bulb. *J. Neurosci.* 30 870–875. 10.1523/JNEUROSCI.4958-09.2010 20089895 PMC6633099

[B194] Leresche N. Asprodini E. Emri Z. Cope D. Crunelli V. (2000). Somatostatin inhibits GABAergic transmission in the sensory thalamus via presynaptic receptors. *Neuroscience* 98 513–522. 10.1016/s0306-4522(00)00107-x 10869845

[B195] Leroy F. de Solis C. A. Boyle L. M. Bock T. Lofaro O. M. Buss E. W. (2021). Enkephalin release from VIP interneurons in the hippocampal CA2/3a region mediates heterosynaptic plasticity and social memory. *Mol. Psychiatry* [Epub ahead of print]. 10.1038/s41380-021-01124-y 33990774 PMC8590711

[B196] Letzkus J. J. Wolff S. B. Meyer E. M. Tovote P. Courtin J. Herry C. (2011). A disinhibitory microcircuit for associative fear learning in the auditory cortex. *Nature* 480 331–335. 10.1038/nature10674 22158104

[B197] Li C. Kim K. (2008). Neuropeptides. *WormBook* 25 1–36. 10.1895/wormbook.1.142.1 18819171 PMC2749236

[B198] Li J. Li J. G. Chen C. Zhang F. Liu-Chen L. Y. (2002). Molecular basis of differences in (-)(trans)-3,4-dichloro-N-methyl-N-[2-(1-pyrrolidiny)-cyclohexyl]benzeneacetamide -induced desensitization and phosphorylation between human and rat kappa-opioid receptors expressed in Chinese hamster ovary cells. *Mol. Pharmacol.* 61 73–84. 10.1124/mol.61.1.73 11752208

[B199] Li Q. Dong C. Li W. Bu W. Wu J. Zhao W. (2014). Neuropeptide Y protects cerebral cortical neurons by regulating microglial immune function. *Neural Regener. Res.* 9:959. 10.4103/1673-5374.133140 25206918 PMC4146213

[B200] Li W. Papilloud A. Lozano-Montes L. Zhao N. Ye X. Zhang X. (2018). Stress impacts the regulation neuropeptides in the rat hippocampus and prefrontal cortex. *Proteomics* 18:e1700408. 10.1002/pmic.201700408 29406625

[B201] Liberzon I. Taylor S. F. Phan K. L. Britton J. C. Fig L. M. Bueller J. A. (2007). Altered central micro-opioid receptor binding after psychological trauma. *Biol. Psychiatry* 61 1030–1038. 10.1016/j.biopsych.2006.06.021 16945349

[B202] Liguz-Lecznar M. Urban-Ciecko J. Kossut M. (2016). Somatostatin and somatostatin-containing neurons in shaping neuronal activity and plasticity. *Front. Neural Circ.* 10:48. 10.3389/fncir.2016.00048 27445703 PMC4927943

[B203] Lin L. C. Sibille E. (2015). Somatostatin, neuronal vulnerability and behavioral emotionality. *Mol. Psychiatry* 20 377–387. 10.1038/mp.2014.184 25600109 PMC4355106

[B204] Lin S. Boey D. Lee N. Schwarzer C. Sainsbury A. Herzog H. (2006). Distribution of prodynorphin mRNA and its interaction with the NPY system in the mouse brain. *Neuropeptides* 40 115–123. 10.1016/j.npep.2005.11.006 16439015

[B205] Liu J. J. Chiu Y. T. DiMattio K. M. Chen C. Huang P. Gentile T. A. (2019). Phosphoproteomic approach for agonist-specific signaling in mouse brains: mTOR pathway is involved in kappa opioid aversion. *Neuropsychopharmacology* 44 939–949. 10.1038/s41386-018-0155-0 30082888 PMC6462019

[B206] Liu R. J. Ota K. T. Dutheil S. Duman R. S. Aghajanian G. K. (2015). Ketamine strengthens CRF-activated amygdala inputs to basal dendrites in mPFC layer V pyramidal cells in the prelimbic but not infralimbic subregion, a key suppressor of stress responses. *Neuropsychopharmacology* 40 2066–2075. 10.1038/npp.2015.70 25759300 PMC4613616

[B207] Loh R. Collins S. Galvez R. (2017b). Neocortical prodynorphin expression is transiently increased with learning: implications for time- and learning-dependent neocortical kappa opioid receptor activation. *Behav. Brain Res.* 335 145–150. 10.1016/j.bbr.2017.08.015 28802836

[B208] Loh R. Chau L. Aijaz A. Wu K. Galvez R. (2017a). Antagonizing the different stages of kappa opioid receptor activation selectively and independently attenuates acquisition and consolidation of associative memories. *Behav. Brain Res.* 323 1–10. 10.1016/j.bbr.2017.01.032 28119127

[B209] Longo S. K. Guo M. G. Ji A. L. Khavari P. A. (2021). Integrating single-cell and spatial transcriptomics to elucidate intercellular tissue dynamics. *Nat. Rev. Genet.* 22 627–644. 10.1038/s41576-021-00370-8 34145435 PMC9888017

[B210] Lukomska A. Dobrzanski G. Liguz-Lecznar M. Kossut M. (2020). Somatostatin receptors (SSTR1-5) on inhibitory interneurons in the barrel cortex. *Brain Struct. Funct.* 225 387–401. 10.1007/s00429-019-02011-7 31873798 PMC6957562

[B211] Maarrawi J. Peyron R. Mertens P. Costes N. Magnin M. Sindou M. (2007a). Differential brain opioid receptor availability in central and peripheral neuropathic pain. *Pain* 127 183–194. 10.1016/j.pain.2006.10.013 17137714

[B212] Maarrawi J. Peyron R. Mertens P. Costes N. Magnin M. Sindou M. (2007b). Motor cortex stimulation for pain control induces changes in the endogenous opioid system. *Neurology* 69 827–834. 10.1212/01.wnl.0000269783.86997.37 17724284

[B213] Magalhaes A. C. Holmes K. D. Dale L. B. Comps-Agrar L. Lee D. Yadav P. N. (2010). CRF receptor 1 regulates anxiety behavior via sensitization of 5-HT2 receptor signaling. *Nat. Neurosci.* 13 622–629. 10.1038/nn.2529 20383137 PMC2862362

[B214] Magistretti P. J. (1990). VIP neurons in the cerebral cortex. *Trends Pharmacol. Sci.* 11 250–254. 10.1016/0165-6147(90)90253-5 2200184

[B215] Magistretti P. J. Hof P. R. Martin J. L. (1986). Adenosine stimulates glycogenolysis in mouse cerebral cortex: a possible coupling mechanism between neuronal activity and energy metabolism. *J. Neurosci.* 6 2558–2562. 10.1523/JNEUROSCI.06-09-02558.1986 3018195 PMC6568677

[B216] Margolis E. B. Lock H. Chefer V. I. Shippenberg T. S. Hjelmstad G. O. Fields H. L. (2006). Kappa opioids selectively control dopaminergic neurons projecting to the prefrontal cortex. *Proc. Natl. Acad. Sci. U.S.A.* 103 2938–2942. 10.1073/pnas.0511159103 16477003 PMC1413839

[B217] Martel G. Dutar P. Epelbaum J. Viollet C. (2012). Somatostatinergic systems: an update on brain functions in normal and pathological aging. *Front. Endocrinol.* 3:154. 10.3389/fendo.2012.00154 23230430 PMC3515867

[B218] Martin J. L. Magistretti P. J. (1989a). Pharmacological studies of voltage-sensitive Ca2+-channels involved in the release of vasoactive intestinal peptide evoked by K+ in mouse cerebral cortical slices. *Neuroscience* 30 423–431. 10.1016/0306-4522(89)90262-5 2546098

[B219] Martin J. L. Magistretti P. J. (1989b). Release of vasoactive intestinal peptide in mouse cerebral cortex: evidence for a role of arachidonic acid metabolites. *J. Neurosci.* 9 2536–2542. 10.1523/JNEUROSCI.09-07-02536.1989 2545840 PMC6569747

[B220] Martin T. F. (1994). The molecular machinery for fast and slow neurosecretion. *Curr. Opin. Neurobiol.* 4 626–632. 10.1016/0959-4388(94)90002-7 7849517

[B221] Mathé A. A. Michaneck M. Berg E. Charney D. S. Murrough J. W. (2020). A randomized controlled trial of intranasal neuropeptide y in patients with major depressive disorder. *Int. J. Neuropsychopharmacol.* 23 783–790. 10.1093/ijnp/pyaa054 33009815 PMC7770516

[B222] Matsui A. Williams J. T. (2011). Opioid-sensitive GABA inputs from rostromedial tegmental nucleus synapse onto midbrain dopamine neurons. *J. Neurosci.* 31 17729–17735. 10.1523/JNEUROSCI.4570-11.2011 22131433 PMC3617570

[B223] Mayfield R. D. Lewohl J. M. Dodd P. R. Herlihy A. Liu J. Harris R. A. (2002). Patterns of gene expression are altered in the frontal and motor cortices of human alcoholics. *J. Neurochem.* 81 802–813. 10.1046/j.1471-4159.2002.00860.x 12065639

[B224] McDonald J. K. Parnavelas J. G. Karamanlidis A. N. Rosenquist G. Brecha N. (1982). The morphology and distribution of peptide-containing neurons in the adult and developing visual cortex of the rat. III. Cholecystokinin. *J. Neurocytol.* 11, 881–895 10.1007/BF01148306 6759621

[B225] McGinty J. F. (1985). Prodynorphin immunoreactivity is located in different neurons than proenkephalin immunoreactivity in the cerebral cortex of rats. *Neuropeptides* 5 465–468. 10.1016/0143-4179(85)90055-1 3889694

[B226] McGinty J. F. van der Kooy D. Bloom F. E. (1984). The distribution and morphology of opioid peptide immunoreactive neurons in the cerebral cortex of rats. *J. Neurosci.* 4 1104–1117. 10.1523/JNEUROSCI.04-04-01104.1984 6143786 PMC6564782

[B227] McLaughlin J. P. Myers L. C. Zarek P. E. Caron M. G. Lefkowitz R. J. Czyzyk T. A. (2004). Prolonged kappa opioid receptor phosphorylation mediated by G-protein receptor kinase underlies sustained analgesic tolerance. *J. Biol. Chem.* 279 1810–1818. 10.1074/jbc.M305796200 14597630 PMC2131729

[B228] McQuiston A. R. Colmers W. F. (1996). Neuropeptide Y2 receptors inhibit the frequency of spontaneous but not miniature EPSCs in CA3 pyramidal cells of rat hippocampus. *J. Neurophysiol.* 76 3159–3168. 10.1152/jn.1996.76.5.3159 8930263

[B229] Melas P. Lennartsson A. Vakifahmetoglu-Norberg H. Wei Y. Åberg E. Werme M. (2013). Allele-specific programming of Npy and epigenetic effects of physical activity in a genetic model of depression. *Transl. Psychiatry* 3:e255. 10.1038/tp.2013.31 23652932 PMC3669918

[B230] Melas P. A. Mannervik M. Mathé A. A. Lavebratt C. (2012). Neuropeptide Y: identification of a novel rat mRNA splice-variant that is downregulated in the hippocampus and the prefrontal cortex of a depression-like model. *Peptides* 35 49–55. 10.1016/j.peptides.2012.02.020 22406386

[B231] Mena J. D. Sadeghian K. Baldo B. A. (2011). Induction of hyperphagia and carbohydrate intake by mu-opioid receptor stimulation in circumscribed regions of frontal cortex. *J. Neurosci.* 31 3249–3260. 10.1523/JNEUROSCI.2050-10.2011 21368037 PMC3131113

[B232] Mena J. D. Selleck R. A. Baldo B. A. (2013). Mu-opioid stimulation in rat prefrontal cortex engages hypothalamic orexin/hypocretin-containing neurons, and reveals dissociable roles of nucleus accumbens and hypothalamus in cortically driven feeding. *J. Neurosci.* 33 18540–18552. 10.1523/JNEUROSCI.3323-12.2013 24259576 PMC3834058

[B233] Meng F. Xie G. X. Thompson R. C. Mansour A. Goldstein A. Watson S. J. (1993). Cloning and pharmacological characterization of a rat kappa opioid receptor. *Proc. Natl. Acad. Sci. U.S.A.* 90 9954–9958. 10.1073/pnas.90.21.9954 8234341 PMC47691

[B234] Meng Q. Y. Chen X. N. Tong D. L. Zhou J. N. (2011). Stress and glucocorticoids regulated corticotropin releasing factor in rat prefrontal cortex. *Mol. Cell Endocrinol.* 342 54–63. 10.1016/j.mce.2011.05.035 21664419

[B235] Mentlein R. (2004). Cell-surface peptidases. *Int. Rev. Cytol.* 235 165–213. 10.1016/S0074-7696(04)35004-7 15219783 PMC7126636

[B236] Merchenthaler I. Maderdrut J. L. Cianchetta P. Shughrue P. Bronstein D. (1997). In situ hybridization histochemical localization of prodynorphin messenger RNA in the central nervous system of the rat. *J. Comp. Neurol.* 384 211–232. 10.1002/(sici)1096-9861(19970728)384:2<211::aid-cne4>3.0.co;2-4 9215719

[B237] Micevych P. E. Yaksh T. L. Go V. W. Finkelstein J. A. (1985). Effect of opiates on the release of cholecystokinin from in vitro hypothalamus and frontal cortex of Zucker lean (Fa/-) and obese (fa/fa) rats. *Brain Res.* 337 382–385. 10.1016/0006-8993(85)90080-0 3896389

[B238] Micevychi P. E. Go V. L. Yaksh T. L. (1984). Simultaneous measurement of cholecystokinin- and vasoactive intestinal polypeptide-like immunoreactivity from cat frontal cortex in vitro: effect of morphine and D-Ala2-D-Leu5-enkephalin. *Brain Res.* 291 55–62. 10.1016/0006-8993(84)90650-4 6697185

[B239] Mickey B. J. Zhou Z. Heitzeg M. M. Heinz E. Hodgkinson C. A. Hsu D. T. (2011). Emotion processing, major depression, and functional genetic variation of neuropeptide Y. *Arch. Gen. Psychiatry* 68 158–166. 10.1001/archgenpsychiatry.2010.197 21300944 PMC3091621

[B240] Milad M. R. Pitman R. K. Ellis C. B. Gold A. L. Shin L. M. Lasko N. B. (2009). Neurobiological basis of failure to recall extinction memory in posttraumatic stress disorder. *Biol. Psychiatry* 66 1075–1082. 10.1016/j.biopsych.2009.06.026 19748076 PMC2787650

[B241] Millan M. A. Samra A. B. Wynn P. C. Catt K. J. Aguilera G. (1987). Receptors and actions of corticotropin-releasing hormone in the primate pituitary gland. *J. Clin. Endocrinol. Metab.* 64 1036–1041. 10.1210/jcem-64-5-1036 3031117

[B242] Millman D. J. Ocker G. K. Caldejon S. Kato I. Larkin J. D. Lee E. K. (2020). VIP interneurons in mouse primary visual cortex selectively enhance responses to weak but specific stimuli. *eLife* 9:e55130. 10.7554/eLife.55130 33108272 PMC7591255

[B243] Minamino N. Kangawa K. Fukuda A. Matsuo H. Iagarashi M. (1980). A new opioid octapeptide related to dynorphin from porcine hypothalamus. *Biochem. Biophys. Res. Commun.* 95 1475–1481. 10.1016/s0006-291x(80)80063-5 7191256

[B244] Molineaux C. J. Cox B. M. (1982). Subcellular localization of immunoreactive dynorphin and vasopressin in rat pituitary and hypothalamus. *Life Sci.* 31 1765–1768. 10.1016/0024-3205(82)90205-3 6130435

[B245] Molosh A. I. Sajdyk T. J. Truitt W. A. Zhu W. Oxford G. S. Shekhar A. (2013). NPY Y1 receptors differentially modulate GABAA and NMDA receptors via divergent signal-transduction pathways to reduce excitability of amygdala neurons. *Neuropsychopharmacology* 38 1352–1364. 10.1038/npp.2013.33 23358240 PMC3656378

[B246] Moore C. B. Guthrie E. H. Huang M. T. Taxman D. J. (2010). Short hairpin RNA (shRNA): design, delivery, and assessment of gene knockdown. *Methods Mol. Biol.* 629 141–158. 10.1007/978-1-60761-657-3_10 20387148 PMC3679364

[B247] Morganstern I. Liang S. Ye Z. Karatayev O. Leibowitz S. F. (2012). Disturbances in behavior and cortical enkephalin gene expression during the anticipation of ethanol in rats characterized as high drinkers. *Alcohol* 46 559–568. 10.1016/j.alcohol.2012.05.003 22703995 PMC3571704

[B248] Morino P. Herrera-Marschitz M. Castel M. N. Ungerstedt U. Varro A. Dockray G. (1994). Cholecystokinin un cortico-striatal neurons in the rat immunohistochemical studies at the light and electron microscopal level. *Eur. J. Neurosci.* 6 681–692. 10.1111/j.1460-9568.1994.tb00980.x 7915604

[B249] Mucha R. F. Herz A. (1985). Motivational properties of kappa and mu opioid receptor agonists studied with place and taste preference conditioning. *Psychopharmacology* 86 274–280. 10.1007/BF00432213 2994144

[B250] Mueller A. L. Kunkel D. D. Schwartzkroin P. A. (1986). Electrophysiological actions of somatostatin (SRIF) in hippocampus: an in vitro study. *Cell. Mol. Neurobiol.* 6 363–379. 10.1007/BF00711406 2881622 PMC11567397

[B251] Murray E. A. Fellows L. K. (2021). Prefrontal cortex interactions with the amygdala in primates. *Neuropsychopharmacology* 47 163–179. 10.1038/s41386-021-01128-w 34446829 PMC8616954

[B252] Naka A. Veit J. Shababo B. Chance R. K. Risso D. Stafford D. (2019). Complementary networks of cortical somatostatin interneurons enforce layer specific control. *eLife* 8:e43696. 10.7554/eLife.43696 30883329 PMC6422636

[B253] Nassel D. R. (2009). Neuropeptide signaling near and far: how localized and timed is the action of neuropeptides in brain circuits? *Invert. Neurosci.* 9 57–75. 10.1007/s10158-009-0090-1 19756790

[B254] Nemeroff C. B. Widerlov E. Bissette G. Walleus H. Karlsson I. Eklund K. (1984). Elevated concentrations of CSF corticotropin-releasing factor-like immunoreactivity in depressed patients. *Science* 226 1342–1344. 10.1126/science.6334362 6334362

[B255] Nevo I. Becker C. Hamon M. Benoliel J. J. (1996). Stress- and yohimbine induced release of cholecystokinin in the frontal cortex of the freely moving rat prevention by diazepam but not ondansetron. *J. Neurochem.* 66 2041–2049. 10.1046/j.1471-4159.1996.66052041.x 8780034

[B256] Nguyen R. Venkatesan S. Binko M. Bang J. Y. Cajanding J. D. Briggs C. (2020). Cholecystokinin-expressing interneurons of the medial prefrontal cortex mediate working memory retrieval. *J. Neurosci.* 40 2314–2331. 10.1523/JNEUROSCI.1919-19.2020 32005764 PMC7083295

[B257] Nosova O. Bazov I. Karpyak V. Hallberg M. Bakalkin G. (2021). Epigenetic and transcriptional control of the opioid prodynorphine gene: in-depth analysis in the human brain. *Molecules* 26:3458. 10.3390/molecules26113458 34200173 PMC8201134

[B258] Nusbaum M. P. Blitz D. M. Marder E. (2017). Functional consequences of neuropeptide and small-molecule co-transmission. *Nat. Rev. Neurosci.* 18 389–403. 10.1038/nrn.2017.56 28592905 PMC5547741

[B259] Obermayer J. Luchicchi A. Heistek T. S. de Kloet S. F. Terra H. Bruinsma B. (2019). Prefrontal cortical ChAT-VIP interneurons provide local excitation by cholinergic synaptic transmission and control attention. *Nat. Commun.* 10:5280. 10.1038/s41467-019-13244-9 31754098 PMC6872593

[B260] Olpe H.-R. Balcar V. J. Bittiger H. Rink H. Sieber P. (1980). Central actions of somatostatin. *Eur. J. Pharmacol.* 63 127–133. 10.1016/0014-2999(80)90436-7 6103812

[B261] Palmer L. M. Shai A. S. Reeve J. E. Anderson H. L. Paulsen O. Larkum M. E. (2014). NMDA spikes enhance action potential generation during sensory input. *Nat. Neurosci.* 17 383–390. 10.1038/nn.3646 24487231

[B262] Pantazopoulos H. Wiseman J. T. Markota M. Ehrenfeld L. Berretta S. (2017). Decreased numbers of somatostatin-expressing neurons in the amygdala of subjects with bipolar disorder or schizophrenia: relationship to circadian rhythms. *Biol. Psychiatry* 81 536–547. 10.1016/j.biopsych.2016.04.006 27259817 PMC5065936

[B263] Park J. Moghaddam B. (2017). Impact of anxiety on prefrontal cortex encoding of cognitive flexibility. *Neuroscience* 345 193–202. 10.1016/j.neuroscience.2016.06.013 27316551 PMC5159328

[B264] Patriarchi T. Cho J. R. Merten K. Marley A. Broussard G. J. Liang R. (2019). Imaging neuromodulators with high spatiotemporal resolution using genetically encoded indicators. *Nat. Protoc.* 14 3471–3505. 10.1038/s41596-019-0239-2 31732722

[B265] Peckys D. Hurd Y. L. (2001). Prodynorphin and kappa opioid receptor mRNA expression in the cingulate and prefrontal cortices of subjects diagnosed with schizophrenia or affective disorders. *Brain Res. Bull.* 55 619–624. 10.1016/s0361-9230(01)00525-1 11576758

[B266] Pedragosa Badia X. Stichel J. Beck-Sickinger A. G. (2013). Neuropeptide Y receptors: how to get subtype selectivity. *Front. Endocrinol.* 4:5. 10.3389/fendo.2013.00005 23382728 PMC3563083

[B267] Peng W. Wu Z. Song K. Zhang S. Li Y. Xu M. (2020). Regulation of sleep homeostasis mediator adenosine by basal forebrain glutamatergic neurons. *Science* 369:eabb0556. 10.1126/science.abb0556 32883833

[B268] Pert C. B. Pert A. Chang J. K. Fong B. T. (1976). (D-Ala2)-Met-enkephalinamide: a potent, long-lasting synthetic pentapeptide analgesic. *Science* 194 330–332. 10.1126/science.968485 968485

[B269] Pfeiffer A. Braun S. Mann K. Meyer H. D. Brantl V. (1986). Anterior pituitary hormone responses to a kappa-opioid agonist in man. *J. Clin. Endocrinol. Metab.* 62 181–185. 10.1210/jcem-62-1-181 3079599

[B270] Pina M. M. Pati D. Hwa L. S. Wu S. Y. Mahoney A. A. Omenyi C. G. (2020). The kappa opioid receptor modulates GABA neuron excitability and synaptic transmission in midbrainprojections from the insular cortex. *Neuropharmacology* 165:107831. 10.1016/j.neuropharm.2019.107831 31870854 PMC7521158

[B271] Poras H. Bonnard E. Dange E. Fournie-Zaluski M. C. Roques B. P. (2014). New orally active dual enkephalinase inhibitors (DENKIs) for central and peripheral pain treatment. *J. Med. Chem.* 57 5748–5763. 10.1021/jm500602h 24927250

[B272] Poyner D. Cox H. Bushfield M. Treherne J. M. Demetrikopoulos M. K. (2000). Neuropeptides in drug research. *Prog. Drug Res.* 54 121–149. 10.1007/978-3-0348-8391-7_4 10857387

[B273] Pradayrol L. Jornvall H. Mutt V. Ribet A. (1980). N-terminally extended somatostatin: the primary structure of somatostatin-28. *FEBS Lett.* 109 55–58. 10.1016/0014-5793(80)81310-x 7353633

[B274] Qian J. Colmers W. F. Saggau P. (1997). Inhibition of synaptic transmission by neuropeptide Y in rat hippocampal area CA1: modulation of presynaptic Ca2+ entry. *J. Neurosci.* 17 8169–8177. 10.1523/JNEUROSCI.17-21-08169.1997 9334392 PMC6573734

[B275] Raadsheer F. C. van Heerikhuize J. J. Lucassen P. J. Hoogendijk W. J. Tilders F. J. Swaab D. F. (1995). Corticotropin-releasing hormone mRNA levels in the paraventricular nucleus of patients with Alzheimer’s disease and depression. *Am. J. Psychiatry* 152 1372–1376. 10.1176/ajp.152.9.1372 7653697

[B276] Radu D. Brodin E. Weber G. Lindefors N. (2001). Delayed stress-induced increase in tissue level of cholecystokinin in rat prefrontal cortex modulation by microdialysis probe implantation and systemic ketamine. *Brain Res.* 908 197–203. 10.1016/s0006-8993(01)02648-8 11454330

[B277] Rajput P. S. Kharmate G. Norman M. Liu S. H. Sastry B. R. Brunicardi C. F. (2011). Somatostatin receptor 1 and 5 double knockout mice mimic neurochemical changes of Huntington’s disease transgenic mice. *PLoS One* 6:e24467. 10.1371/journal.pone.0024467 21912697 PMC3166321

[B278] Ran F. A. Hsu P. D. Wright J. Agarwala V. Scott D. A. Zhang F. (2013). Genome engineering using the CRISPR-Cas9 system. *Nat. Protoc.* 8 2281–2308. 10.1038/nprot.2013.143 24157548 PMC3969860

[B279] Rangon C. M. Goursaud S. Medja F. Lelievre V. Mounien L. Husson I. (2005). VPAC2 receptors mediate vasoactive intestinal peptide-induced neuroprotection against neonatal excitotoxic brain lesions in mice. *J. Pharmacol. Exp. Ther.* 314 745–752. 10.1124/jpet.105.086405 15872042

[B280] Refojo D. Echenique C. Muller M. B. Reul J. M. Deussing J. M. Wurst W. (2005). Corticotropin-releasing hormone activates ERK1/2 MAPK in specific brain areas. *Proc. Natl. Acad. Sci. U.S.A.* 102 6183–6188. 10.1073/pnas.0502070102 15833812 PMC1087957

[B281] Reinikainen K. J. Koponen H. Jolkkonen J. Riekkinen P. J. (1990). Decreased somatostatin-like immunoreactivity in the cerebrospinal fluid of chronic schizophrenic patients with cognitive impairment. *Psychiatry Res.* 33 307–312. 10.1016/0165-1781(90)90047-9 2243905

[B282] Reisi P. Ghaedamini A. R. Golbidi M. Shabrang M. Arabpoor Z. Rashidi B. (2015). Effect of cholecystokinin on learning and memory, neuronal proliferation and apoptosis in the rat hippocampus. *Adv. Biomed. Res.* 4:227. 10.4103/2277-9175.166650 26623402 PMC4638054

[B283] Reisine T. Bell G. I. (1995). Molecular biology of somatostatin receptors. *Endocr. Rev.* 16 427–442. 10.1210/edrv-16-4-427 8521788

[B284] Robbins R. J. Landon R. M. (1985). The effects of neurotensin, vasoactive intestinal polypeptide and other neuropeptides on the secretion of somatostatin from cerebral cortical cells. *Brain Res.* 332 161–164. 10.1016/0006-8993(85)90400-7 2859907

[B285] Roberts J. G. Sombers L. A. (2018). Fast-scan cyclic voltammetry: chemical sensing in the brain and beyond. *Anal. Chem.* 90 490–504. 10.1021/acs.analchem.7b04732 29182309 PMC5750125

[B286] Robich M. P. Matyal R. Chu L. M. Feng J. Xu S.-H. Laham R. J. (2010). Effects of neuropeptide Y on collateral development in a swine model of chronic myocardial ischemia. *J. Mol. Cell. Cardiol.* 49 1022–1030. 10.1016/j.yjmcc.2010.08.022 20826160 PMC2975818

[B287] Rosenbaum D. M. Rasmussen S. G. Kobilka B. K. (2009). The structure and function of G-protein-coupled receptors. *Nature* 459 356–363. 10.1038/nature08144 19458711 PMC3967846

[B288] Roth B. L. (2019). Molecular pharmacology of metabotropic receptors targeted by neuropsychiatric drugs. *Nat. Struct. Mol. Biol.* 26 535–544. 10.1038/s41594-019-0252-8 31270468 PMC6613815

[B289] Roy A. Pandey S. C. (2002). The decreased cellular expression of neuropeptide Y protein in rat brain structures during ethanol withdrawal after chronic ethanol exposure. *Alcohol. Clin. Exp. Res.* 26 796–803. 10.1097/00000374-200206000-00008 12068247

[B290] Rubinow D. R. Gold P. W. Post R. M. Ballenger J. C. (1985). CSF somatostatin in affective illness and normal volunteers. *Prog. Neuropsychopharmacol. Biol. Psychiatry* 9 393–400. 10.1016/0278-5846(85)90192-7 2866561

[B291] Rudy B. Fishell G. Lee S. Hjerling-Leffler J. (2011). Three groups of interneurons account for nearly 100% of neocortical GABAergic neurons. *Dev. Neurobiol.* 71 45–61. 10.1002/dneu.20853 21154909 PMC3556905

[B292] Russo A. F. (2017). Overview of neuropeptides: awakening the senses? *Headache* 57 (Suppl. 2) 37–46. 10.1111/head.13084 28485842 PMC5424629

[B293] Sah R. Geracioti T. (2013). Neuropeptide Y and posttraumatic stress disorder. *Mol. Psychiatry* 18 646–655. 10.1038/mp.2012.101 22801411 PMC4749915

[B294] Sajdyk T. J. Vandergriff M. G. Gehlert D. R. (1999). Amygdalar neuropeptide Y Y1 receptors mediate the anxiolytic-like actions of neuropeptide Y in the social interaction test. *Eur. J. Pharmacol.* 368 143–147. 10.1016/s0014-2999(99)00018-7 10193650

[B295] Salio C. Lossi L. Ferrini F. Merighi A. (2006). Neuropeptides as synaptic transmitters. *Cell Tissue Res.* 326 583–598. 10.1007/s00441-006-0268-3 16847638

[B296] Savasta M. Palacios J. M. Mengod G. (1988). Regional localization of the mRNA coding for the neuropeptide cholecystokinin in the rat brain studied by in situ hybridization. *Neurosci. Lett.* 93 132–138. 10.1016/0304-3940(88)90070-5 3241637

[B297] Sawchenko P. Swanson L. Grzanna R. Howe P. Bloom S. Polak J. (1985). Colocalization of neuropeptide Y immunoreactivity in brainstem catecholaminergic neurons that project to the paraventricular nucleus of the hypothalamus. *J. Comp. Neurol.* 241 138–153. 10.1002/cne.902410203 3840810

[B298] Sayed S. Van Dam N. T. Horn S. R. Kautz M. M. Parides M. Costi S. (2018). A randomized dose-ranging study of neuropeptide Y in patients with posttraumatic stress disorder. *Int. J. Neuropsychopharmacol.* 21 3–11. 10.1093/ijnp/pyx109 29186416 PMC5795352

[B299] Schafer M. K.-H. Day R. Watson S. J. Akil H. (1991). “Distribution of opioids in brain and peripheral tissues,” in *Neurobiology of Opioids*, eds Almeida O. F. X. Shippenberg T. S. (Berlin: Springer), 53–71. 10.1007/978-3-642-46660-1_3

[B300] Schally A. V. Huang W. Y. Chang R. C. Arimura A. Redding T. W. Millar R. P. (1980). Isolation and structure of pro-somatostatin: a putative somatostatin precursor from pig hypothalamus. *Proc. Natl. Acad. Sci. U.S.A.* 77 4489–4493. 10.1073/pnas.77.8.4489 6107906 PMC349869

[B301] Schmidt M. J. Horvath S. Ebert P. Norris J. L. Seeley E. H. Brown J. (2014). Modulation of behavioral networks by selective interneuronal inactivation. *Mol. Psychiatry* 19 580–587. 10.1038/mp.2013.167 24322205 PMC4179403

[B302] Schwarzer C. (2009). 30 years of dynorphins–new insights on their functions in neuropsychiatric diseases. *Pharmacol. Ther.* 123 353–370. 10.1016/j.pharmthera.2009.05.006 19481570 PMC2872771

[B303] Selleck R. A. Lake C. Estrada V. Riederer J. Andrzejewski M. Sadeghian K. (2015). Endogenous opioid signaling in the medial prefrontal cortex is required for the expression of hunger-induced impulsive action. *Neuropsychopharmacology* 40 2464–2474. 10.1038/npp.2015.97 25865930 PMC4538362

[B304] Senese N. B. Kandasamy R. Kochan K. E. Traynor J. R. (2020). Regulator of G-protein signaling (RGS) protein modulation of opioid receptor signaling as a potential target for pain management. *Front. Mol. Neurosci.* 13:5. 10.3389/fnmol.2020.00005 32038168 PMC6992652

[B305] Sessler F. M. Grady S. M. Waterhouse B. D. Moises H. C. (1991). Electrophysiological actions of VIP in rat somatosensory cortex. *Peptides* 12 715–721. 10.1016/0196-9781(91)90124-8 1788134

[B306] Shields B. C. Kahuno E. Kim C. Apostolides P. F. Brown J. Lindo S. (2017). Deconstructing behavioral neuropharmacology with cellular specificity. *Science* 356:eaaj2161. 10.1126/science.aaj2161 28385956

[B307] Siegel R. A. Duker E. M. Fuchs E. Pahnke U. Wuttke W. (1984). Responsiveness of mesolimbic, mesocortical, septal and hippocampal cholecystokinin and substance P neuronal systems to stress, in the male rat. *Neurochem. Int.* 6 783–789. 10.1016/0197-0186(84)90011-1 20488108

[B308] Silberman Y. Bajo M. Chappell A. M. Christian D. T. Cruz M. Diaz M. R. (2009). Neurobiological mechanisms contributing to alcohol-stress-anxiety interactions. *Alcohol* 43 509–519. 10.1016/j.alcohol.2009.01.002 19913194 PMC2814297

[B309] Sirohi S. Walker B. M. (2015). Maturational alterations in constitutive activity of medial prefrontal cortex kappa-opioid receptors in Wistar rats. *J. Neurochem.* 135 659–665. 10.1111/jnc.13279 26257334 PMC4636924

[B310] Slater P. G. Cerda C. A. Pereira L. A. Andres M. E. Gysling K. (2016). CRF binding protein facilitates the presence of CRF type 2alpha receptor on the cell surface. *Proc. Natl. Acad. Sci. U.S.A.* 113 4075–4080. 10.1073/pnas.1523745113 27035969 PMC4839449

[B311] Smialowska M. Zieba B. Domin H. (2021). A role of noradrenergic receptors in anxiolytic-like effect of high CRF in the rat frontal cortex. *Neuropeptides* 88:102162. 10.1016/j.npep.2021.102162 34062382

[B312] Smith S. J. Sumbul U. Graybuck L. T. Collman F. Seshamani S. Gala R. (2019). Single-cell transcriptomic evidence for dense intracortical neuropeptide networks. *eLife* 8:e47889. 10.7554/eLife.47889 31710287 PMC6881117

[B313] Snyder S. H. Innis R. B. (1979). Peptide neurotransmitters. *Annu. Rev. Biochem.* 48 755–782. 10.1146/annurev.bi.48.070179.003543 38738

[B314] Sohn J. Hioki H. Okamoto S. Kaneko T. (2014). Preprodynorphin-expressing neurons constitute a large subgroup of somatostatin-expressing GABAergic interneurons in the mouse neocortex. *J. Comp. Neurol.* 522 1506–1526. 10.1002/cne.23477 24122731

[B315] Song Y. H. Hwang Y. S. Kim K. Lee H. R. Kim J. H. Maclachlan C. (2020). Somatostatin enhances visual processing and perception by suppressing excitatory inputs to parvalbumin-positive interneurons in V1. *Sci. Adv.* 6:eaaz0517. 10.1126/sciadv.aaz0517 32494634 PMC7176413

[B316] Song Y. H. Yoon J. Lee S. H. (2021). The role of neuropeptide somatostatin in the brain and its application in treating neurological disorders. *Exp. Mol. Med.* 53 328–338. 10.1038/s12276-021-00580-4 33742131 PMC8080805

[B317] Spetea M. Asim M. F. Wolber G. Schmidhammer H. (2013). The micro opioid receptor and ligands acting at the micro opioid receptor, as therapeutics and potential therapeutics. *Curr. Pharm. Des.* 19 7415–7434. 10.2174/13816128113199990362 23448479

[B318] Spierling S. R. Zorrilla E. P. (2017). Don’t stress about CRF: assessing the translational failures of CRF1antagonists. *Psychopharmacology* 234 1467–1481. 10.1007/s00213-017-4556-2 28265716 PMC5420464

[B319] Stengel A. Tache Y. F. (2017). Activation of brain somatostatin signaling suppresses CRF receptor-mediated stress response. *Front. Neurosci.* 11:231. 10.3389/fnins.2017.00231 28487631 PMC5403923

[B320] Stoeber M. Jullie D. Lobingier B. T. Laeremans T. Steyaert J. Schiller P. W. (2018). A genetically encoded biosensor reveals location bias of opioid drug action. *Neuron* 98 963.e5–976.e5. 10.1016/j.neuron.2018.04.021 29754753 PMC6481295

[B321] Sudhof T. C. (2012). Calcium control of neurotransmitter release. *Cold Spring Harb. Perspect. Biol.* 4:a011353. 10.1101/cshperspect.a011353 22068972 PMC3249630

[B322] Sun H. Luessen D. J. Kind K. O. Zhang K. Chen R. (2020). Cocaine self-administration regulates transcription of opioid peptide precursors and opioid receptors in rat caudate putamen and prefrontal cortex. *Neuroscience* 443 131–139. 10.1016/j.neuroscience.2020.07.035 32730947 PMC7484423

[B323] Sun Q. Q. Akk G. Huguenard J. R. Prince D. A. (2001). Differential regulation of GABA release and neuronal excitability mediated by neuropeptide Y1 and Y2 receptors in rat thalamic neurons. *J. Physiol.* 531 81–94. 10.1111/j.1469-7793.2001.0081j.x 11179393 PMC2278458

[B324] Svingos A. L. Colago E. E. (2002). Kappa-Opioid and NMDA glutamate receptors are differentially targeted within rat medial prefrontal cortex. *Brain Res.* 946 262–271. 10.1016/s0006-8993(02)02894-9 12137930

[B325] Svingos A. L. Colago E. E. Pickel V. M. (1999). Cellular sites for dynorphin activation of kappa-opioid receptors in the rat nucleus accumbens shell. *J. Neurosci.* 19 1804–1813. 10.1523/JNEUROSCI.19-05-01804.1999 10024364 PMC6782165

[B326] Takahashi A. (2016). “Subchapter 7A - enkephalin,” in *Handbook of Hormones*, eds Yoshio Takei H. A. Tsutsui K. (Cambridge, MA: Academic Press), 55–57.

[B327] Taki K. Kaneko T. Mizuno N. (2000). A group of cortical interneurons expressing mu-opioid receptor-like immunoreactivity: a double immunofluorescence study in the rat cerebral cortex. *Neuroscience* 98 221–231. 10.1016/s0306-4522(00)00124-x 10854753

[B328] Tallent M. K. (2008). Presynaptic inhibition of glutamate release by neuropeptides: use-dependent synaptic modification. *Results Probl. Cell Differ.* 44 177–200. 10.1007/400_2007_037 17554500

[B329] Tan H. Zhong P. Yan Z. (2004). Corticotropin-releasing factor and acute stress prolongs serotonergic regulation of GABA transmission in prefrontal cortical pyramidal neurons. *J. Neurosci.* 24 5000–5008. 10.1523/JNEUROSCI.0143-04.2004 15163692 PMC6729364

[B330] Tang J. Yang H. Y. Costa E. (1982). Distribution of met5-enkephalin-Arg6-Phe7 (MEAP) in various tissues of rats and guinea pigs. *Life Sci.* 31 2303–2306. 10.1016/0024-3205(82)90143-6 7162346

[B331] Tang Q. Lynch R. M. Porreca F. Lai J. (2000). Dynorphin A elicits an increase in intracellular calcium in cultured neurons via a non-opioid, non-NMDA mechanism. *J. Neurophysiol.* 83 2610–2615. 10.1152/jn.2000.83.5.2610 10805661

[B332] Tang X. L. Wang Y. Li D. L. Luo J. Liu M. Y. (2012). Orphan G protein-coupled receptors (GPCRs): biological functions and potential drug targets. *Acta Pharmacol. Sin.* 33 363–371. 10.1038/aps.2011.210 22367282 PMC4077139

[B333] Tapia-Arancibia L. Pares-Herbute N. Astier H. (1989). Calcium dependence of somatostatin (SRIF) release and cyclic AMP levels in cultured diencephalic neurons. *Neuroendocrinology* 49 555–560. 10.1159/000125167 2566943

[B334] Taqi M. M. Bazov I. Watanabe H. Sheedy D. Harper C. Alkass K. (2011). Prodynorphin CpG-SNPs associated with alcohol dependence: elevated methylation in the brain of human alcoholics. *Addict. Biol.* 16 499–509. 10.1111/j.1369-1600.2011.00323.x 21521424 PMC3391609

[B335] Tasic B. Yao Z. Graybuck L. T. Smith K. A. Nguyen T. N. Bertagnolli D. (2018). Shared and distinct transcriptomic cell types across neocortical areas. *Nature* 563 72–78. 10.1038/s41586-018-0654-5 30382198 PMC6456269

[B336] Tatemoto K. (1982). Neuropeptide Y: complete amino acid sequence of the brain peptide. *Proc. Natl. Acad. Sci. U.S.A.* 79 5485–5489. 10.1073/pnas.79.18.5485 6957876 PMC346928

[B337] Taussig R. Iniguez-Lluhi J. A. Gilman A. G. (1993). Inhibition of adenylyl cyclase by Gi alpha. *Science* 261 218–221. 10.1126/science.8327893 8327893

[B338] Tejeda H. A. Counotte D. S. Oh E. Ramamoorthy S. Schultz-Kuszak K. N. Backman C. M. (2013). Prefrontal cortical kappa-opioid receptor modulation of local neurotransmission and conditioned place aversion. *Neuropsychopharmacology* 38 1770–1779. 10.1038/npp.2013.76 23542927 PMC3717537

[B339] Tejeda H. A. Hanks A. N. Scott L. Mejias-Aponte C. Hughes Z. A. O’Donnell P. (2015). Prefrontal cortical kappa opioid receptors attenuate responses to amygdala inputs. *Neuropsychopharmacology* 40 2856–2864. 10.1038/npp.2015.138 25971593 PMC4864622

[B340] Tejeda H. A. Shippenberg T. S. Henriksson R. (2012). The dynorphin/kappa-opioid receptor system and its role in psychiatric disorders. *Cell Mol. Life Sci.* 69 857–896. 10.1007/s00018-011-0844-x 22002579 PMC11114766

[B341] Tejeda H. A. Wang H. Flores R. J. Yarur H. E. (2021). Dynorphin/kappa-opioid receptor system modulation of cortical circuitry. *Handb. Exp. Pharmacol.* 271 223–253. 10.1007/164_2021_440 33580392

[B342] Thal L. Laing K. Horowitz S. Makman M. (1986). Dopamine stimulates rat cortical somatostatin release. *Brain Res.* 372 205–209. 10.1016/0006-8993(86)91126-1 2871899

[B343] Thorsell A. Mathé A. A. (2017). Neuropeptide Y in alcohol addiction and affective disorders. *Front. Endocrinol.* 8:178. 10.3389/fendo.2017.00178 28824541 PMC5534438

[B344] Toll L. Berzetei-Gurske I. P. Polgar W. E. Brandt S. R. Adapa I. D. Rodriguez L. (1998). Standard binding and functional assays related to medications development division testing for potential cocaine and opiate narcotic treatment medications. *NIDA Res. Monogr.* 178 440–466. 9686407

[B345] Tremblay R. Lee S. Rudy B. (2016). GABAergic interneurons in the neocortex: from cellular properties to circuits. *Neuron* 91 260–292. 10.1016/j.neuron.2016.06.033 27477017 PMC4980915

[B346] Trieu B. H. Remmers B. C. Toddes C. Brandner D. D. Lefevre E. M. Kocharian A. (2022). Angiotensin-converting enzyme gates brain circuit-specific plasticity via an endogenous opioid. *Science* 375 1177–1182. 10.1126/science.abl5130 35201898 PMC9233526

[B347] Tripp A. Kota R. S. Lewis D. A. Sibille E. (2011). Reduced somatostatin in subgenual anterior cingulate cortex in major depression. *Neurobiol. Dis.* 42 116–124. 10.1016/j.nbd.2011.01.014 21232602 PMC3039077

[B348] Truitt W. A. Johnson P. L. Dietrich A. D. Fitz S. D. Shekhar A. (2009). Anxiety-like behavior is modulated by a discrete subpopulation of interneurons in the basolateral amygdala. *Neuroscience* 160 284–294. 10.1016/j.neuroscience.2009.01.083 19258024 PMC2682359

[B349] Ungless M. A. Singh V. Crowder T. L. Yaka R. Ron D. Bonci A. (2003). Corticotropin-releasing factor requires CRF binding protein to potentiate NMDA receptors via CRF receptor 2 in dopamine neurons. *Neuron* 39 401–407. 10.1016/s0896-6273(03)00461-6 12895416

[B350] Urban-Ciecko J. Barth A. L. (2016). Somatostatin-expressing neurons in cortical networks. *Nat. Rev. Neurosci.* 17 401–409. 10.1038/nrn.2016.53 27225074 PMC5635659

[B351] Uribe-Marino A. Gassen N. C. Wiesbeck M. F. Balsevich G. Santarelli S. Solfrank B. (2016). Prefrontal cortex corticotropin-releasing factor receptor 1 conveys acute stress-induced executive dysfunction. *Biol. Psychiatry* 80 743–753. 10.1016/j.biopsych.2016.03.2106 27318500

[B352] Vale W. Spiess J. Rivier C. Rivier J. (1981). Characterization of a 41-residue ovine hypothalamic peptide that stimulates secretion of corticotropin and beta-endorphin. *Science* 213 1394–1397. 10.1126/science.6267699 6267699

[B353] van den Pol A. N. (2012). Neuropeptide transmission in brain circuits. *Neuron* 76 98–115. 10.1016/j.neuron.2012.09.014 23040809 PMC3918222

[B354] Van Pett K. Viau V. Bittencourt J. C. Chan R. K. Li H. Y. Arias C. (2000). Distribution of mRNAs encoding CRF receptors in brain and pituitary of rat and mouse. *J. Comp. Neurol.* 428 191–212. 10.1002/1096-9861(20001211)428:2<191::aid-cne1>3.0.co;2-u 11064361

[B355] Van Reeth O. Goldman S. Schiffmann S. Verstappen A. Pelletier G. Vaudry H. (1987). Distribution of neuropeptide Y immunoreactivity in human visual cortex and underlying white matter. *Peptides* 8 1107–1117. 10.1016/0196-9781(87)90144-6 3441446

[B356] Vanderhaeghen J. J. Lotstra F. Vierendeels G. Gilles C. Deschepper C. Verbanck P. (1981). Cholecystokinins in the central nervous system and neurohypophysis. *Peptides* 2 (Suppl. 2) 81–88. 10.1016/0196-9781(81)90016-4 6283501

[B357] Vezzani A. Sperk G. (2004). Overexpression of NPY and Y2 receptors in epileptic brain tissue: an endogenous neuroprotective mechanism in temporal lobe epilepsy? *Neuropeptides* 38 245–252. 10.1016/j.npep.2004.05.004 15337376

[B358] Vollmer L. L. Schmeltzer S. Schurdak J. Ahlbrand R. Rush J. Dolgas C. M. (2016). Neuropeptide Y impairs retrieval of extinguished fear and modulates excitability of neurons in the infralimbic prefrontal cortex. *J. Neurosci.* 36 1306–1315. 10.1523/JNEUROSCI.4955-13.2016 26818517 PMC6604823

[B359] Wagner J. J. Evans C. J. Chavkin C. (1991). Focal stimulation of the mossy fibers releases endogenous dynorphins that bind kappa 1-opioid receptors in guinea pig hippocampus. *J. Neurochem.* 57 333–343. 10.1111/j.1471-4159.1991.tb02132.x 1675664

[B360] Wall P. M. Flinn J. Messier C. (2001). Infralimbic muscarinic M1 receptors modulate anxiety-like behaviour and spontaneous working memory in mice. *Psychopharmacology* 155 58–68. 10.1007/s002130000671 11374337

[B361] Wall P. M. Messier C. (2002). Infralimbic kappa opioid and muscarinic M1 receptor interactions in the concurrent modulation of anxiety and memory. *Psychopharmacology* 160 233–244. 10.1007/s00213-001-0979-9 11889492

[B362] Wang H. Jing M. Li Y. (2018). Lighting up the brain: genetically encoded fluorescent sensors for imaging neurotransmitters and neuromodulators. *Curr. Opin. Neurobiol.* 50 171–178. 10.1016/j.conb.2018.03.010 29627516 PMC5984720

[B363] Wang H. L. Bogen C. Reisine T. Dichter M. (1989). Somatostatin-14 and somatostatin-28 induce opposite effects on potassium currents in rat neocortical neurons. *Proc. Natl. Acad. Sci. U.S.A.* 86 9616–9620. 10.1073/pnas.86.23.9616 2574465 PMC298549

[B364] Wang J. Tian Y. Zeng L. H. Xu H. (2020). Prefrontal disinhibition in social fear: a vital action of somatostatin interneurons. *Front Cell Neurosci* 14:611732. 10.3389/fncel.2020.611732 33390908 PMC7773700

[B365] Wang J.-Y. Yaksh T. L. Go V. L. (1985). Studies on the in vivo release of vasoactive intestinal polypeptide (VIP) from the cerebral cortex: effects of cortical, brainstem and somatic stimuli. *Brain Res.* 326 317–334. 10.1016/0006-8993(85)90042-3 2982463

[B366] Wang J. Y. Yaksh T. L. Harty G. J. Go V. L. (1986). Neurotransmitter modulation of VIP release from cat cerebral cortex. *Am. J. Physiol.* 250(1 Pt 2), R104–R111. 10.1152/ajpregu.1986.250.1.R104 2867688

[B367] Weis W. I. Kobilka B. K. (2018). The molecular basis of G protein-coupled receptor activation. *Annu. Rev. Biochem.* 87 897–919. 10.1146/annurev-biochem-060614-033910 29925258 PMC6535337

[B368] Whitnall M. H. Gainer H. Cox B. M. Molineaux C. J. (1983). Dynorphin-A-(1-8) is contained within vasopressin neurosecretory vesicles in rat pituitary. *Science* 222 1137–1139. 10.1126/science.6648526 6648526

[B369] Widdowson P. S. Ordway G. A. Halaris A. E. (1992). Reduced neuropeptide Y concentrations in suicide brain. *J. Neurochem.* 59 73–80. 10.1111/j.1471-4159.1992.tb08877.x 1613514

[B370] Williams J. A. Sans M. D. Tashiro M. Schäfer C. Bragado M. J. Dabrowski A. (2002). Cholecystokinin activates a variety of intracellular signal transduction mechanisms in rodent pancreatic acinar cells. *Pharmacol Toxicol.* 91 297–303. 10.1034/j.1600-0773.2002.910606.x 12688372

[B371] Willoch F. Schindler F. Wester H. J. Empl M. Straube A. Schwaiger M. (2004). Central poststroke pain and reduced opioid receptor binding within pain processing circuitries: a [11C]diprenorphine PET study. *Pain* 108 213–220. 10.1016/j.pain.2003.08.014 15030940

[B372] Wingler L. M. Lefkowitz R. J. (2020). Conformational basis of G protein-coupled receptor signaling versatility. *Trends Cell Biol.* 30 736–747. 10.1016/j.tcb.2020.06.002 32622699 PMC7483927

[B373] Wohleb E. S. Franklin T. Iwata M. Duman R. S. (2016). Integrating neuroimmune systems in the neurobiology of depression. *Nat. Rev. Neurosci.* 17 497–511. 10.1038/nrn.2016.69 27277867

[B374] Wood D. E. Nusbaum M. P. (2002). Extracellular peptidase activity tunes motor pattern modulation. *J. Neurosci.* 22 4185–4195. 10.1523/JNEUROSCI.22-10-04185.2002 12019336 PMC6757626

[B375] Wootten D. Christopoulos A. Marti-Solano M. Babu M. M. Sexton P. M. (2018). Mechanisms of signalling and biased agonism in G protein-coupled receptors. *Nat. Rev. Mol. Cell Biol.* 19 638–653. 10.1038/s41580-018-0049-3 30104700

[B376] Yakovleva T. Bazov I. Cebers G. Marinova Z. Hara Y. Ahmed A. (2006). Prodynorphin storage and processing in axon terminals and dendrites. *FASEB J.* 20 2124–2126. 10.1096/fj.06-6174fje 16966485

[B377] Yan X. X. Baram T. Z. Gerth A. Schultz L. Ribak C. E. (1998). Co-localization of corticotropin-releasing hormone with glutamate decarboxylase and calcium-binding proteins in infant rat neocortical interneurons. *Exp. Brain Res.* 123 334–340. 10.1007/s002210050576 9860272 PMC3786772

[B378] Yang K. Trepanier C. H. Li H. Beazely M. A. Lerner E. A. Jackson M. F. (2009). Vasoactive intestinal peptide acts via multiple signal pathways to regulate hippocampal NMDA receptors and synaptic transmission. *Hippocampus* 19 779–789. 10.1002/hipo.20559 19173226 PMC2736340

[B379] Yang Y. Lee P. Sternson S. M. (2015). Cell type-specific pharmacology of NMDA receptors using masked MK801. *eLife* 4:10206. 10.7554/eLife.10206 26359633 PMC4594264

[B380] Yarur H. E. Gonzalez M. P. Verbel-Vergara D. Andres M. E. Gysling K. (2020a). Cross-talk between dopamine D1 and corticotropin releasing factor type 2 receptors leads to occlusion of their ERK1/2 signaling. *J. Neurochem.* 155 264–273. 10.1111/jnc.15016 32215915

[B381] Yarur H. E. Vega-Quiroga I. Gonzalez M. P. Noches V. Thomases D. R. Andres M. E. (2020b). Inhibitory control of basolateral amygdalar transmission to the prefrontal cortex by local corticotrophin type 2 receptor. *Int. J. Neuropsychopharmacol.* 23 108–116. 10.1093/ijnp/pyz065 31800046 PMC7094000

[B382] You Z. B. Herrera-Marschitz M. Brodin E. Meana J. J. Morino P. Hökfelt T. (1993). On the origin of striatal cholecystokinin (CCK) release studies with in vivo microdialysis. *J. Neurochem.* 62 76–85. 10.1046/j.1471-4159.1994.62010076.x 7903356

[B383] Yuferov V. Nielsen D. A. Levran O. Randesi M. Hamon S. Ho A. (2011). Tissue-specific DNA methylation of the human prodynorphin gene in post-mortem brain tissues and PBMCs. *Pharmacogenet. Genomics* 21 185–196. 10.1097/FPC.0b013e32833eecbc 20808262 PMC3017726

[B384] Zastrow M. V. Sorkin A. (2021). Mechanisms for regulating and organizing receptor signaling by endocytosis. *Annu. Rev. Biochem.* 90 709–737. 10.1146/annurev-biochem-081820-092427 33606955 PMC8608402

[B385] Zelikowsky M. Ding K. Anderson D. J. (2018). Neuropeptidergic control of an internal brain state produced by prolonged social isolation stress. *Cold Spring Harb. Symp. Quant. Biol.* 83 97–103. 10.1101/sqb.2018.83.038109 30948452

[B386] Zhang Z. Wu Y. Wang Z. Dunning F. M. Rehfuss J. Ramanan D. (2011). Release mode of large and small dense-core vesicles specified by different synaptotagmin isoforms in PC12 cells. *Mol. Biol. Cell* 22 2324–2336. 10.1091/mbc.E11-02-0159 21551071 PMC3128534

[B387] Zhou Z. Zhu G. Hariri A. R. Enoch M.-A. Scott D. Sinha R. (2008). Genetic variation in human NPY expression affects stress response and emotion. *Nature* 452 997–1001. 10.1038/nature06858 18385673 PMC2715959

[B388] Zieba B. Grzegorzewska M. Branski P. Domin H. Wieronska J. M. Hess G. (2008). The behavioural and electrophysiological effects of CRF in rat frontal cortex. *Neuropeptides* 42 513–523. 10.1016/j.npep.2008.05.004 18617263

[B389] Zobel A. W. Nickel T. Kunzel H. E. Ackl N. Sonntag A. Ising M. (2000). Effects of the high-affinity corticotropin-releasing hormone receptor 1 antagonist R121919 in major depression: the first 20 patients treated. *J. Psychiatr. Res.* 34 171–181. 10.1016/s0022-3956(00)00016-9 10867111

[B390] Zorrilla E. P. Logrip M. L. Koob G. F. (2014). Corticotropin releasing factor: a key role in the neurobiology of addiction. *Front. Neuroendocrinol.* 35 234–244. 10.1016/j.yfrne.2014.01.001 24456850 PMC4213066

