# Supplementary material for: Neuropeptide System Regulation of Prefrontal Cortex Circuitry: Implications for Neuropsychiatric Disorders
Source: Front Neural Circuits. 2022 Jun 21;16:796443. doi: 10.3389/fncir.2022.796443 (PMC9255232; doi:10.3389/fncir.2022.796443)
Supplement: Supplementary file 1 [file Presentation_1.PPTX]

## Slide 1
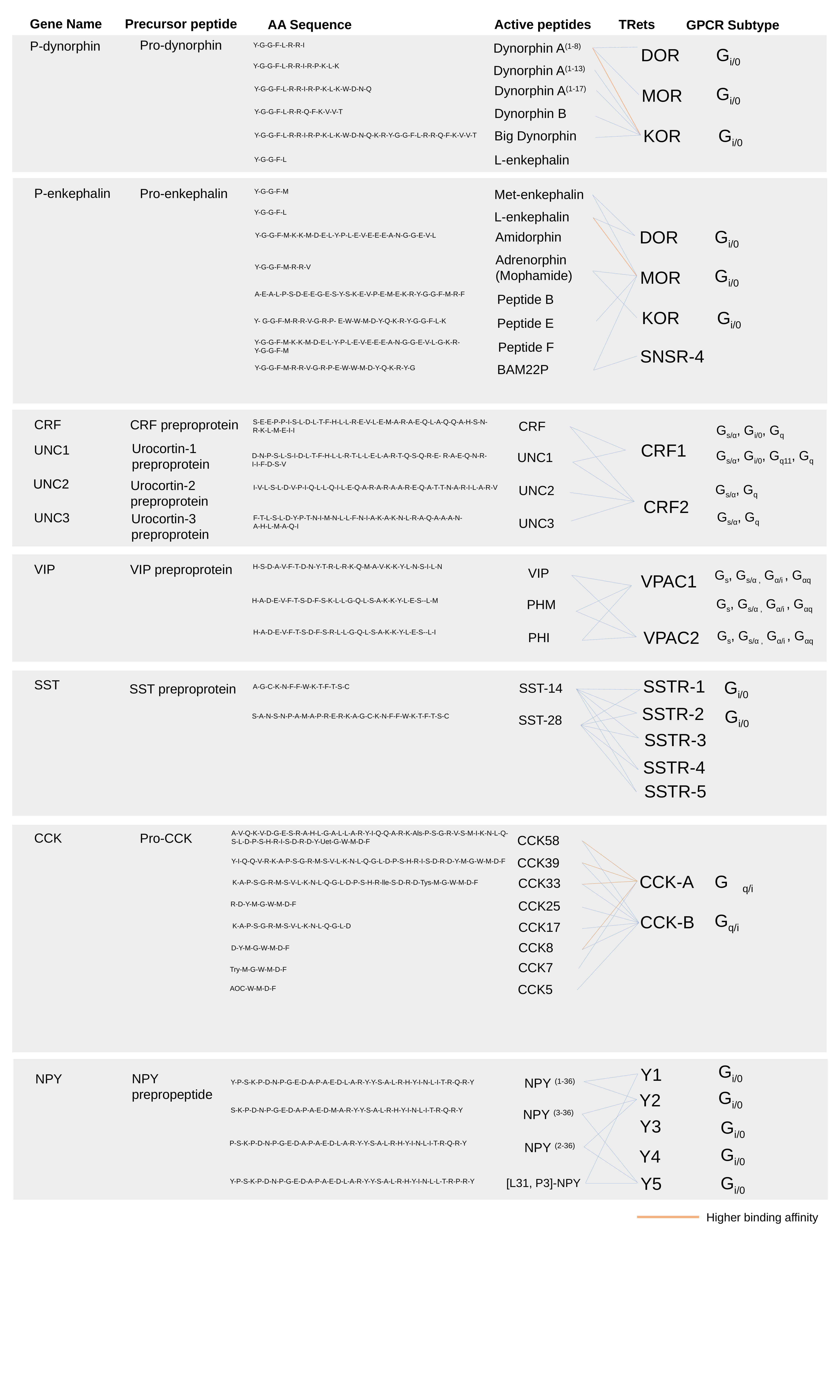

Precursor peptide
Gene Name
Active peptides
TRets
AA Sequence
GPCR Subtype
Pro-dynorphin
P-dynorphin
Dynorphin A(1-8)
Y-G-G-F-L-R-R-I
Gi/0
DOR
Y-G-G-F-L-R-R-I-R-P-K-L-K
Dynorphin A(1-13)
Dynorphin A(1-17)
Gi/0
MOR
Y-G-G-F-L-R-R-I-R-P-K-L-K-W-D-N-Q
Dynorphin B
Y-G-G-F-L-R-R-Q-F-K-V-V-T
Gi/0
KOR
Big Dynorphin
Y-G-G-F-L-R-R-I-R-P-K-L-K-W-D-N-Q-K-R-Y-G-G-F-L-R-R-Q-F-K-V-V-T
L-enkephalin
Y-G-G-F-L
P-enkephalin
Pro-enkephalin
Met-enkephalin
Y-G-G-F-M
Y-G-G-F-L
L-enkephalin
Gi/0
DOR
Amidorphin
Y-G-G-F-M-K-K-M-D-E-L-Y-P-L-E-V-E-E-E-A-N-G-G-E-V-L
Adrenorphin (Mophamide)
Y-G-G-F-M-R-R-V
Gi/0
MOR
A-E-A-L-P-S-D-E-E-G-E-S-Y-S-K-E-V-P-E-M-E-K-R-Y-G-G-F-M-R-F
Peptide B
Gi/0
KOR
Peptide E
Y- G-G-F-M-R-R-V-G-R-P- E-W-W-M-D-Y-Q-K-R-Y-G-G-F-L-K
Y-G-G-F-M-K-K-M-D-E-L-Y-P-L-E-V-E-E-E-A-N-G-G-E-V-L-G-K-R-Y-G-G-F-M
Peptide F
SNSR-4
BAM22P
Y-G-G-F-M-R-R-V-G-R-P-E-W-W-M-D-Y-Q-K-R-Y-G
CRF
CRF preproprotein
S-E-E-P-P-I-S-L-D-L-T-F-H-L-L-R-E-V-L-E-M-A-R-A-E-Q-L-A-Q-Q-A-H-S-N-R-K-L-M-E-I-I
CRF
Gs/α, Gi/0, Gq
CRF1
Urocortin-1 preproprotein
UNC1
Gs/α, Gi/0, Gq11, Gq
UNC1
D-N-P-S-L-S-I-D-L-T-F-H-L-L-R-T-L-L-E-L-A-R-T-Q-S-Q-R-E- R-A-E-Q-N-R-I-I-F-D-S-V
UNC2
UNC2
Urocortin-2 preproprotein
Gs/α, Gq
UNC2
I-V-L-S-L-D-V-P-I-Q-L-L-Q-I-L-E-Q-A-R-A-R-A-A-R-E-Q-A-T-T-N-A-R-I-L-A-R-V
CRF2
Gs/α, Gq
UNC3
Urocortin-3 preproprotein
F-T-L-S-L-D-Y-P-T-N-I-M-N-L-L-F-N-I-A-K-A-K-N-L-R-A-Q-A-A-A-N-A-H-L-M-A-Q-I
UNC3
VIP
VIP preproprotein
H-S-D-A-V-F-T-D-N-Y-T-R-L-R-K-Q-M-A-V-K-K-Y-L-N-S-I-L-N
VIP
Gs, Gs/α , Gα/i , Gαq
VPAC1
Gs, Gs/α , Gα/i , Gαq
H-A-D-E-V-F-T-S-D-F-S-K-L-L-G-Q-L-S-A-K-K-Y-L-E-S--L-M
PHM
VPAC2
Gs, Gs/α , Gα/i , Gαq
H-A-D-E-V-F-T-S-D-F-S-R-L-L-G-Q-L-S-A-K-K-Y-L-E-S--L-I
PHI
SSTR-1
SST
Gi/0
SST-14
SST preproprotein
A-G-C-K-N-F-F-W-K-T-F-T-S-C
SSTR-2
Gi/0
SST-28
S-A-N-S-N-P-A-M-A-P-R-E-R-K-A-G-C-K-N-F-F-W-K-T-F-T-S-C
SSTR-3
SSTR-4
SSTR-5
A-V-Q-K-V-D-G-E-S-R-A-H-L-G-A-L-L-A-R-Y-I-Q-Q-A-R-K-Als-P-S-G-R-V-S-M-I-K-N-L-Q-S-L-D-P-S-H-R-I-S-D-R-D-Y-Uet-G-W-M-D-F
CCK
Pro-CCK
CCK58
CCK39
Y-I-Q-Q-V-R-K-A-P-S-G-R-M-S-V-L-K-N-L-Q-G-L-D-P-S-H-R-I-S-D-R-D-Y-M-G-W-M-D-F
G	q/i
CCK-A
CCK33
K-A-P-S-G-R-M-S-V-L-K-N-L-Q-G-L-D-P-S-H-R-lle-S-D-R-D-Tys-M-G-W-M-D-F
CCK25
R-D-Y-M-G-W-M-D-F
Gq/i
CCK-B
CCK17
K-A-P-S-G-R-M-S-V-L-K-N-L-Q-G-L-D
CCK8
D-Y-M-G-W-M-D-F
CCK7
Try-M-G-W-M-D-F
CCK5
AOC-W-M-D-F
Gi/0
Y1
NPY prepropeptide
NPY
NPY (1-36)
Y-P-S-K-P-D-N-P-G-E-D-A-P-A-E-D-L-A-R-Y-Y-S-A-L-R-H-Y-I-N-L-I-T-R-Q-R-Y
Gi/0
Y2
S-K-P-D-N-P-G-E-D-A-P-A-E-D-M-A-R-Y-Y-S-A-L-R-H-Y-I-N-L-I-T-R-Q-R-Y
NPY (3-36)
Y3
Gi/0
P-S-K-P-D-N-P-G-E-D-A-P-A-E-D-L-A-R-Y-Y-S-A-L-R-H-Y-I-N-L-I-T-R-Q-R-Y
NPY (2-36)
Gi/0
Y4
Gi/0
Y5
[L31, P3]-NPY
Y-P-S-K-P-D-N-P-G-E-D-A-P-A-E-D-L-A-R-Y-Y-S-A-L-R-H-Y-I-N-L-L-T-R-P-R-Y
Higher binding affinity
